# Exploration of Structured Symmetric Cyclic Peptides as Ligands for Metal-Organic Frameworks

**DOI:** 10.1021/acs.chemmater.2c02597

**Published:** 2022-10-25

**Authors:** Meerit Y. Said, Christine S. Kang, Shunzhi Wang, William Sheffler, Patrick J. Salveson, Asim K. Bera, Alex Kang, Hannah Nguyen, Ryanne Ballard, Xinting Li, Hua Bai, Lance Stewart, Paul Levine, David Baker

**Affiliations:** † Institute for Protein Design, University of Washington, 4000 15th Avenue NE, Seattle, Washington 98195, United States; ‡ Department of Biochemistry, University of Washington, 4000 15th Avenue NE, Seattle, Washington 98195, United States; § Howard Hughes Medical Institute, University of Washington, Seattle, Washington 98195, United States

## Abstract

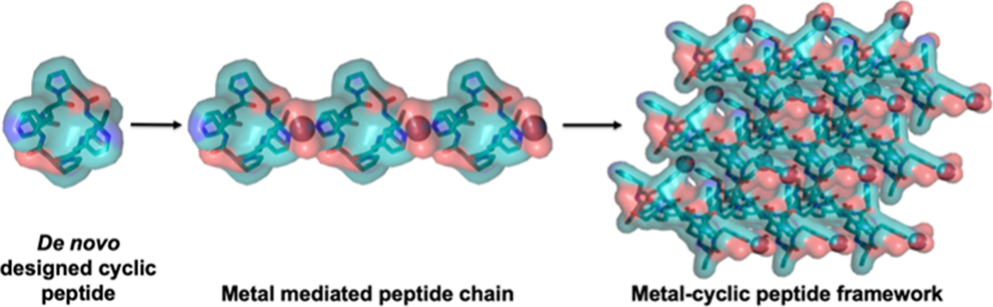

Despite remarkable advances in the assembly of highly structured coordination polymers and metal–organic frameworks, the rational design of such materials using more conformationally flexible organic ligands such as peptides remains challenging. In an effort to make the design of such materials fully programmable, we first developed a computational design method for generating metal-mediated 3D frameworks using rigid and symmetric peptide macrocycles with metal-coordinating sidechains. We solved the structures of six crystalline networks involving conformationally constrained 6 to 12 residue cyclic peptides with C2, C3, and S2 internal symmetry and three different types of metals (Zn^2+^, Co^2+^, or Cu^2+^) by single-crystal X-ray diffraction, which reveals how the peptide sequences, backbone symmetries, and metal coordination preferences drive the assembly of the resulting structures. In contrast to smaller ligands, these peptides associate through peptide–peptide interactions without full coordination of the metals, contrary to one of the assumptions underlying our computational design method. The cyclic peptides are the largest peptidic ligands reported to form crystalline coordination polymers with transition metals to date, and while more work is required to develop methods for fully programming their crystal structures, the combination of high chemical diversity with synthetic accessibility makes them attractive building blocks for engineering a broader set of new crystalline materials for use in applications such as sensing, asymmetric catalysis, and chiral separation.

## Introduction

Guided by a set of topological and chemical principles, combinations of organic ligands and metals have been used to engineer crystals formed from a wide variety of coordination polymers, including metal–organic frameworks (MOFs).^1−6^ Recently developed chemically tailorable organic linkers enable the modification of the pore geometry and internal surface chemistry of open-framework materials for applications such as adsorption, separation, and catalysis.^7−13^ The most commonly explored MOF ligands utilize rigid conjugated aromatic linkers,^14,15^ while the development of such materials using larger flexible ligands remains more limited. Peptidic ligands have recently emerged as an attractive class of MOF building blocks due to their intrinsic chirality, structural modularity, and biocompatibility.^16−20^ However, the majority of reported metal-peptide frameworks to date involve short linear peptides (e.g., di- and tripeptides) identified through the large-scale experimental screening.^21−24^ Use of longer peptides as organic linkers in this way has been challenging because of their greater conformational flexibility.

We previously demonstrated that Rosetta computational design methods could be used to design cyclic peptides with internally symmetric sequences and structures.^25^ The crystal structures of nine C2, C3, and S2 peptides were very close to the computational design models. Here, we set out to explore the design of MOFs using these symmetric cyclic peptides with well-defined backbone structures as metal ligands. These compounds have potential advantages over previous peptide ligands as they are more rigid and have internal symmetry axes that can be aligned with crystal lattice symmetry axes, and hence we reasoned that materials generated using them should be more programmable. We aimed to design specific MOF lattices using geometrically compatible symmetric peptides and metal sites; the combination of two symmetry elements in defined orientations generates regularly repeating lattices.^26^ We reasoned that by combining the internal symmetries of the cyclic peptide backbones and metal coordination centers through defined rotations, translations, and dihedral angles associated with the peptide sidechains coordinating the metal, a wide variety of crystal lattices could be generated. Experimental characterization of a series of symmetric cyclic peptide-metal systems, which involve some of the largest peptidic ligands reported to form crystalline coordination polymers with transition metals to date, reveals limitations in our starting assumptions and highlights a set of principles underlying the structures of such assemblies.

## Experimental Section

### Symmetric Backbone Generation

Backbone structures were generated for C3 and S2 symmetric macrocycles by sampling the backbone dihedral angles of the asymmetric unit (3 residues for a nine-residue C3 peptide) using kinematic closure to drive chain closure with internal symmetry as described in Mulligan et al.^25^ C2 symmetric macrocycles were generated by systematically sampling the space of conformations for the asymmetric unit, computing the rigid body transformation associated with these, and selecting those for which duplication generates a closed structure (i.e., those for which the angle of rotation around the symmetry axis is 180°). The energy of the designed peptide conformation was calculated using AIMNet.^27^

### Metal-Mediated Crystal Lattice Design

Peptide backbones generated as described above were placed into metal-mediated lattices by choosing a set of metal binding sidechains and metal coordination geometries, and then, for each choice, placing a metal binding sidechain at each position in the asymmetric unit and sampling the chi angles in 1-degree steps and analytically computing rotation around the sidechain-metal bond that produces the correct angle between the peptide and metal symmetry axes, resulting in a macrocycle with 2 (C2 and S2) or 3 (C3) metal coordinating residues. The crystal lattice is finally generated through the placement of additional copies of the macrocycle to fill out each metal coordination sphere. Lattices containing clashes between neighboring macrocycle backbones were removed, and the amino acids not involved in the metal coordination designed using Rosetta (Listing S7) to favor the internal geometry of the macrocycle, the packing interactions between macrocycles, and the positioning of the metal coordinating residues. The resulting designed crystal lattices were filtered based on density (calculated using the script in Listing S9).

This design approach is similar in principle to that of King et al. and Hsia et al.,^28,29^ wherein distinct symmetry elements are placed so they propagate into the desired assembly. A top-down approach was used by King et al., placing proteins with cyclic symmetry along the axes of the target cage symmetry, for example, C4 and C3 at the faces and corners of a cube, then sampling the rotations and translations along these axes that preserve symmetry.^28^ A bottom-up approach was used in Hsia et al., fusing proteins with cyclic symmetry through helical repeat linker elements and searching for fusions which place the symmetry elements relative to each other to form a target symmetry, for example, forming a cube with C4 and C3 elements 54.7° apart such that the axes intersect.^29^ The bottom-up approach we use here to design crystal lattices starting with symmetric peptides and searching for possible binding geometries that attach a symmetric metal coordination site goes beyond the previous approaches in several ways. First, here the relationship between symmetry elements is defined by rotamer and metal binding geometry rather than protein–protein interactions or backbone–backbone fusion. Second, we design three-dimensional crystal assemblies requiring more complex geometric criteria, precision, and careful alignment to the unit cell. Third, we employ small peptide scaffolds with d- and l-amino acids rather than large all-L proteins. Fourth, we considered D2 symmetry elements as well as cyclic elements. Consideration of d-amino acids and D2 symmetry elements expands the space of possible symmetric assemblies and metal binding geometries but is otherwise straightforward. Placement of symmetry elements to form 3D crystals requires higher precision than in other symmetric design tasks, as small errors can propagate much further in the assembly before self-reinforcement. For example, C4 elements on the faces of a cube require only three steps to come back on itself, while a *P*2_1_3 crystal requires 10 steps.

To calculate the void volume in each crystal structure, water was removed from the structures (Figure S3) and then the percent void in a unit cell was calculated using Mercury’s default settings as described in Macrae et al.^30^

### Peptide Synthesis and Purification

All peptides were purchased from WuXi Apptec or synthesized in-house on a CEM Liberty Blue microwave synthesizer. All l- and d-amino acids were purchased from P3 Biosystems. Oxyma Pure was purchased from CEM, DIC was purchased from Oakwood Chemical, diisopropyl ethylamine (DIEA) and piperidine were purchased from Sigma-Aldrich. Dimethylformamide (DMF) was purchased from Fisher Scientific and treated with an Aldraamine trapping pack prior to use. Synthesis was done on a 0.1 mmol scale on CEM Cl-TCP(Cl) resin. Five equivalents of each amino acid were activated using 0.1 M Oxyma with 2% (v/v) DIEA in DMF, 15.4% (v/v) DIC, and coupled on resin for 4 min with double coupling if needed. This was followed by deprotection using 5 mL of 20% piperidine in DMF for 2 min at 95 °C. Completed linear peptides were removed from resin while maintaining side chain protecting groups by 5 times 5 min incubations of the resin in 1% TFA in dichloromethane (DCM). The DCM was removed in vacuo and the protected peptides were subjected to lyophilization in a 1:1 water/acetonitrile (ACN) mixture. The protected peptides were resuspended in 70 mL of DCM in a 100 mL round bottom flask, treated with 1.1 equivalents of (7-azabenzotriazol-1-yloxy)tripyrrolidinophosphonium hexafluorophosphate (PyAOP), and stirred for 30 min before adding 0.2% (v/v) DIEA dropwise. The cyclization reaction proceeded for 16 h before removing DCM in vacuo and subjecting the peptide to a total deprotection solution consisting of TFA/H2O/DODT (3,6-dioxa-1,8-octanedithiol)/triisopropylsilane (92.5:2.5:2.5:2.5) for 3 h. This deprotection mixture was precipitated in 30 mL of ice-cold ethyl ether, centrifuged and decanted, then washed twice more with fresh ether and dried under nitrogen to yield crude peptide for high pressure liquid chromatography (HPLC) purification.

The crude peptide was dried and dissolved in a mixture of ACN and water where the entire crude is soluble. This solution was purified on a C18 column in an Agilent HPLC instrument. A linear gradient of increasing ACN with 0.1% TFA was used to purify the samples. UV signal was monitored at 214 nm and all peaks were collected. Peaks were checked using ESI mass spectroscopy for the correct peptide mass. The purified peptide was then lyophilized for further use. All UPLC and mass spectra are included in the Supporting Information.

### Crystal Screening

Peptides were screened using 96 well plates using the conditions shown in Supporting Information Tables 1 and 2. Stocks of the peptides were made in water, methanol, acetonitrile, or DMF so that 1.25–5 mM are added to each well. The peptide samples were left to dry on the plate overnight, then 5 μL of the appropriate solvent was added to each well. Completed plates were incubated at 4°C overnight and then checked using a light microscope for crystal formation. If no crystals form, the plates would be placed in a convection oven at 80 °C. Conditions that grow crystalline material are optimized in polymerase chain reaction tubes through varying peptide concentration and solvent conditions. Once crystals formed, diffraction data were collected from a single crystal at synchrotron (on APS 24ID-C) and at 100 K. Unit cell refinement and data reduction were performed using the XDS and CCP4 suites.^31,32^ The structure was identified by direct methods and refined by full-matrix least-squares on F2 with anisotropic displacement parameters for the non-H atoms using SHELXL-2018/3.^33,34^ Structure analysis was aided by using Coot/Shelxle.^35,36^ The hydrogen atoms on heavy atoms were calculated in ideal positions with isotropic displacement parameters set to 1.2× Ueq of the attached atoms. Crystallographic structures were deposited into the Cambridge Structural Database (CSD), under deposition numbers 2160569 (C2-1), 2160570 (C2-2a), 2160571 (C2-2b), 2160572 (C3-1), 2160573 (C3-2), 2160589 (S2-1), and 2160766 (S2-2).

## Results

We previously described a symmetric cyclic peptide design method that generates peptide sequences predicted to have single low-energy states with internal symmetry.^25,37^ The method starts by generating large numbers of cyclic peptide backbones with internal symmetry, searches for low energy sequences for these backbones, and then checks by folding simulations that the lowest energy conformation matches the designed conformation. In our previous work, we designed large numbers of such compounds in silico. We were able to solve the crystal structures of 12 of these and found that they were very close to the design models, including one peptide designed to switch from one conformation into another in the presence of zinc (both conformations were confirmed crystallographically).^25^ To generate coordination polymers using these rigid symmetric structures as building blocks, we incorporated metal liganding amino acid side chains into the structures, confirming by in silico energy landscape mapping that the lowest energy predicted states were not affected by the amino acid substitutions (Figure S1).

**Figure 1:**
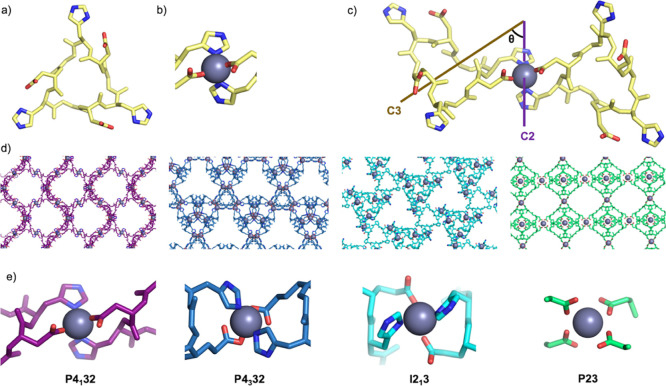
Computational method for designing metal-mediated 3D lattices from rigid symmetric peptide building blocks. (a) Rotamers of metal coordinating residues such as histidines are sampled on symmetric peptide backbones. (b) Symmetric metal-mediated interactions between pairs of peptides are sampled according to standard coordination geometry. (c) View down the dihedral axis between the axis of symmetry of the peptide and the axis of symmetry of the metal compatible with ideal lattice geometry for particular space groups are selected. (d) Examples of modeled lattices in space groups *P*4_3_32, *P*4_1_32, *P*23, and *I*2_1_3. (e) Close up view of the metal coordination for each lattice.

We developed a computational method for docking and designing such symmetric cyclic peptides into crystal lattices with metal-mediated interfaces based on three simplifying assumptions. First, that the internal structures of the peptides would be maintained in the metal mediated crystal lattices; second, that the peptides would fully coordinate metals with preferred tetrahedral geometry such as Zn^2+^ ions; and third that all metal coordinating residues would be involved in the metal coordination (e.g., that peptides with one histidine and one aspartate residue would coordinate the metal in a two-His, two-Asp configuration, Figure 1a,b). We took a bottom-up approach, starting with symmetric peptides and searching through possible interaction geometries through symmetric metal coordination sites. The peptide and metal symmetry elements are placed relative to each other based on the coordinating residue position and rotamer, as well as the metal-residue bond (for more detail, see Experimental Section). To form a 3D crystal, the axes of the component symmetry elements must be placed at precise dihedral angles (Figure 1c). In cases where the peptide to metal connection is a single residue, the metal-residue bond can be rotated to form the correct dihedral angle between the axis of symmetry of the peptide and the axis of symmetry of the metal site. In other cases, the correct dihedral angle must be screened for (Figure 1c). We also considered a two-residue bidentate ASP-HIS binding motif, forming an overall C2 symmetric metal site around a tetrahedral metal center (Figure 1e). ASP-HIS pairs were precomputed and indexed, then superimposed on the peptide scaffolds. In this case, there is no rotatable metal-peptide bond, so not all structures have the appropriate dihedral angle between symmetry elements, and many must be discarded. To generate a crystal lattice in a specific crystal space group, in addition to component symmetry elements forming the correct dihedral angle between their axes, they must be placed at specific locations within the crystal unit cell, and there must not be clashes between symmetrically related copies; evaluating these properties is lattice-dependent. In the case of a C3 peptide and a C3 metal center, a *P*2_1_3 crystal can be formed with one C3 axis along [1,1,1] and intersecting the origin, and the other along [1,1,-1] and intersecting the [0,1,0] axis. The cell dimension is, in this case, defined by the distance from the origin to the [0,1,0] intersection. In the case of a C3 peptide and a bidentate C2 binding site, an *I*2_1_3 crystal can be formed in a similar manner. In the case of a C3 peptide and a tetrahedral metal site, the fully coordinated site has D2 local symmetry and can form a *P*23 crystal by placing the D2 element axis-aligned with the center along the [1,1,0] axis. The C3 element is aligned to [1,1,1], and the system scales such that the C3 axis intersects the [2̅,1,1] axis. In this third case, the placement of the D2 and C3 elements each imply a unit cell dimension, and only systems where these cell dimensions agree are valid. This pipeline produced models in the *P*4_3_32, *P*4_1_32, *P*23, and *I*2_1_3 space groups (Figure 1d,e), which were designed using Rosetta (Supporting Information methods). Designed lattices with very low energies (as computed by Rosetta), cell dimensions less than 50 Å, and an approximate solvent fraction of less than 0.8 were selected for further analysis.^38,39^

To increase the diversity of structures that could be generated, in addition to the cyclic peptides whose structures were previously determined crystallographically (in the absence of metals), we included as potential building blocks the larger in silico set of designs predicted to adopt low-energy symmetric states. We selected 48 C3 peptide crystals generated from these compounds in the *I*2_1_3, *P*23, *P*4_1_32, and *P*4_3_32 space groups with Zn^2+^ as a metal-ligand for crystal assembly.^40^ The cyclic peptide ligands were synthesized in-house using the previously described methods or obtained from WuXi AppTec.^25^ To sample a wide condition space for crystallization and reduce the mass of peptide required for each individual reaction, we performed high-throughput screening experiments in 5 μL of volume using 96-well plates. In a typical experiment, 1 to 2.5 mM peptide was mixed with a metal source [e.g., Zn(NO_3_)_2_, Fe(NO_3_)_3_, Cu(NO_3_)_2_, or Co(NO_3_)_2_] at various molar ratios, in the presence of aqueous buffer solution (HEPES pH 7.0–8.5 or MES pH 5.0–7.0), or mixtures of organic solvents (DMF, DEF, MeOH, EtOH, and/or ACN) (Tables S1 and S2). The reaction mixtures were sealed and reacted for 24–48 h at either room temperature or at an elevated temperature (e.g., 65 or 80 °C) in a convection oven.

**Figure 2:**
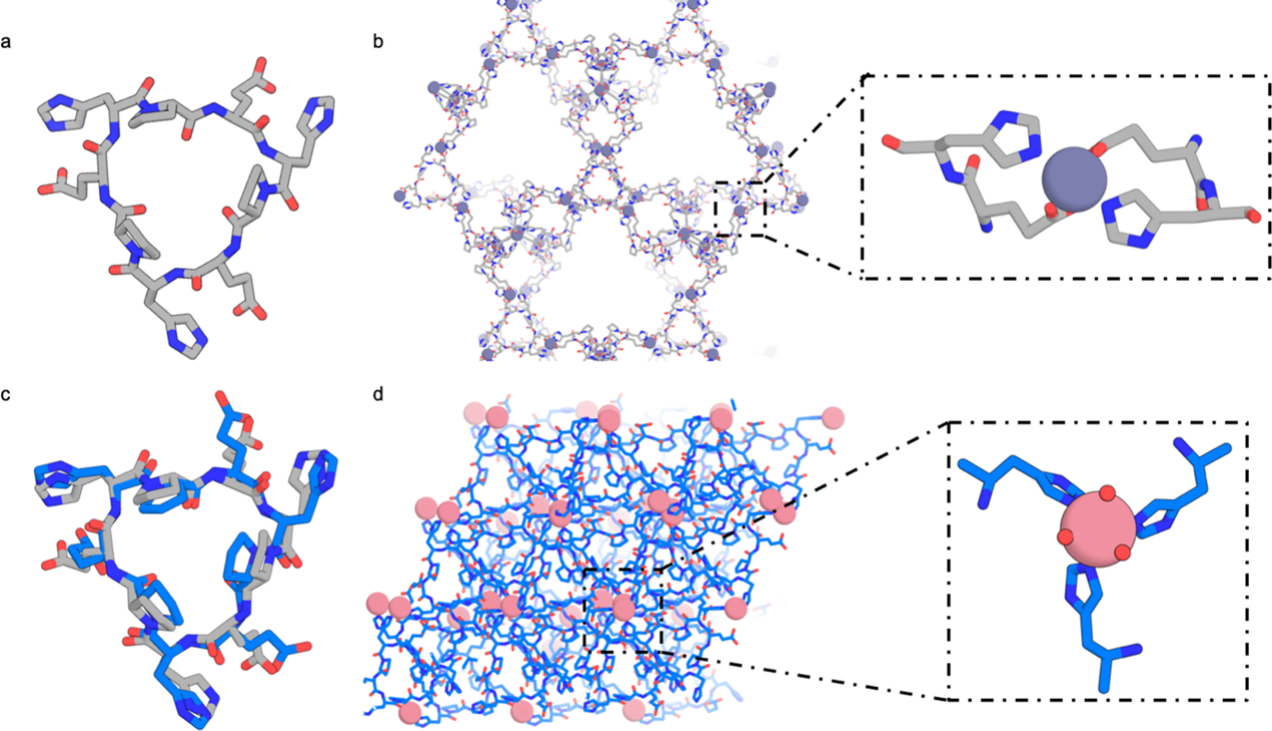
Structure of the C3-1 (EhPEhPEhP)–Co^2+^ crystal. (a) Monomer computational design model. (b) *P*4_3_32 lattice design model. The inset shows the tetrahedral metal coordination in the design model. (c) Crystal structure of the C3-1 ligand (blue) aligned with the design model (gray) over the monomer, with a 0.59 Å Cα RMSD. (d) Crystal structure of C3-1 in *P*65 space group. The inset shows the three histidines and three waters coordination of C3-1 in the crystal structure.

Crystallization studies reveal that many of the designed peptides formed aggregates in the presence of metals. However,two peptides crystallized, but the structures could not be solved due to their low resolution (Table S3 and Figure S4). We were able to solve the structure of one peptide C3-1 (EhPEhPEhP, Figure 2a), which in the designed crystal lattice (*P*4_3_32 space group) was intended to coordinate tetrahedral metals such as zinc with histidines and glutamates (Figure 2b). We were unable to crystallize the peptide using Zn(OAc)_2_, Zn(NO_3_)_2_, or ZnCl_2_, but in the presence of Co(NO_3_)_2_ in HEPES pH 8.2, crystals grew in the *P*65 space group over 4 weeks at room temperature (Figure 2d), and we were able to solve the structure at 0.86 Å resolution. The peptide backbone conformation matches the design with a Cα root mean square deviation (RMSD) of 0.59 Å (Figure 2c). However, in the design model, the metal ion is fully coordinated by the glutamic acids and histidines, while in the crystal structure each Co^2+^ cation is octahedrally coordinated to three water molecules and three histidines from different peptides in a planar fashion (Figure 2b), and the glutamates do not participate in coordination but fill the crystal pores. This coordination geometry leads to the formation of 2D planes with 3-fold symmetry (Figure S6a), which stack at a 60-degree offset angle (Figure S6b) along the c-axis to form a 6-layer repeat unit (Figure S6b, teal dashed lines). The 3D lattice is stabilized by dispersion interactions and hydrogen bonding between the peptide planes and is more dense than the design model (void volume of 40% compared to 91%, see Experimental Section for void calculations).^30^ Thus, while the internal conformation of the peptide matches the design model, the interactions between peptides are quite different than in the design model, with favorable peptide–peptide interactions outweighing the energetic gain from full metal coordination. These results suggest that our assumption that the lowest energy states would involve full metal coordination may not hold generally.

To gain further insight into the balance between peptide–peptide and peptide-metal interactions in determining MOF structures, we carried out a bottom-up exploration of peptides with variable symmetries (C2, C3, and S2), incorporated non-canonical metal coordinating residues [3-(4-pyridyl)-alanine, DOPA, or 4-carboxy-phenylalanine], and generated five additional structures, which we describe in the following sections.

**Figure 3:**
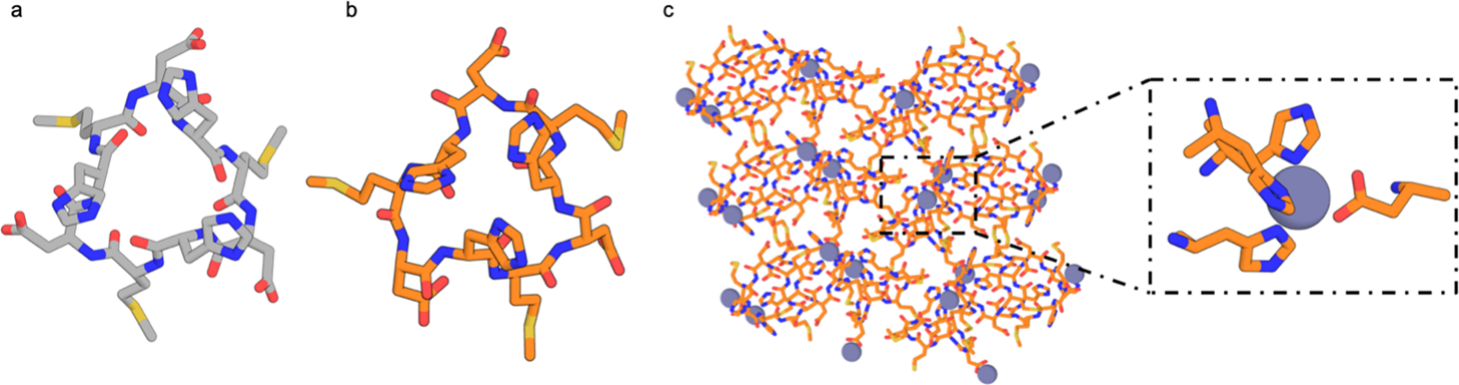
Structure of the C3-2 (DhmDhmDhm): Zn^2+^ crystal. (a) Design model of peptide C3-2. (b) Crystal structure of peptide C3-2. (c) Crystal structure of the peptide in the *P*4_1_2_1_2 space group. Inset view shows tetrahedrally coordinated zinc in the crystal structure with three histidines from one peptide and one aspartate from an adjacent peptide.

A nine-residue peptide (DhmDhmDhm, C3-2, Figure 3a), crystallized in the *P*4_1_2_1_2 space group in the presence of 1 equivalent of Zn(NO_3_)_2_ in MES pH 6, at 80 °C for 24 h (Figure 3c). In contrast to the C3-1 crystal, in which the peptide conformation was nearly identical to the design model, the C3-2 peptide conformation in the metal-mediated crystal is different from the original design model. This is due to a change in the torsional angle of the coordinating histidine (Figure 3b); such metal-induced changes have been observed previously.^25,41^ The backbone conformation is still C3 symmetric, but the side chain rotamers are not symmetric. The zinc ion is internally coordinated with three histidines from one peptide and aspartic acid from an adjacent peptide. The crystal is composed of 1D metal-mediated peptide chains that intercross to form a dense 3D lattice (18% calculated void volume). In the crystal, two peptide-metal chains are intertwined via dispersion interactions (Figure S7a), and the other uncoordinated aspartic acid side chains form polar interactions with the peptide backbones (Figure S7a, purple dashed circle). Such interactions are also observed with the poly-proline-containing peptide-metal frameworks synthesized by Schnitzer and colleagues.^17^

**Figure 4:**
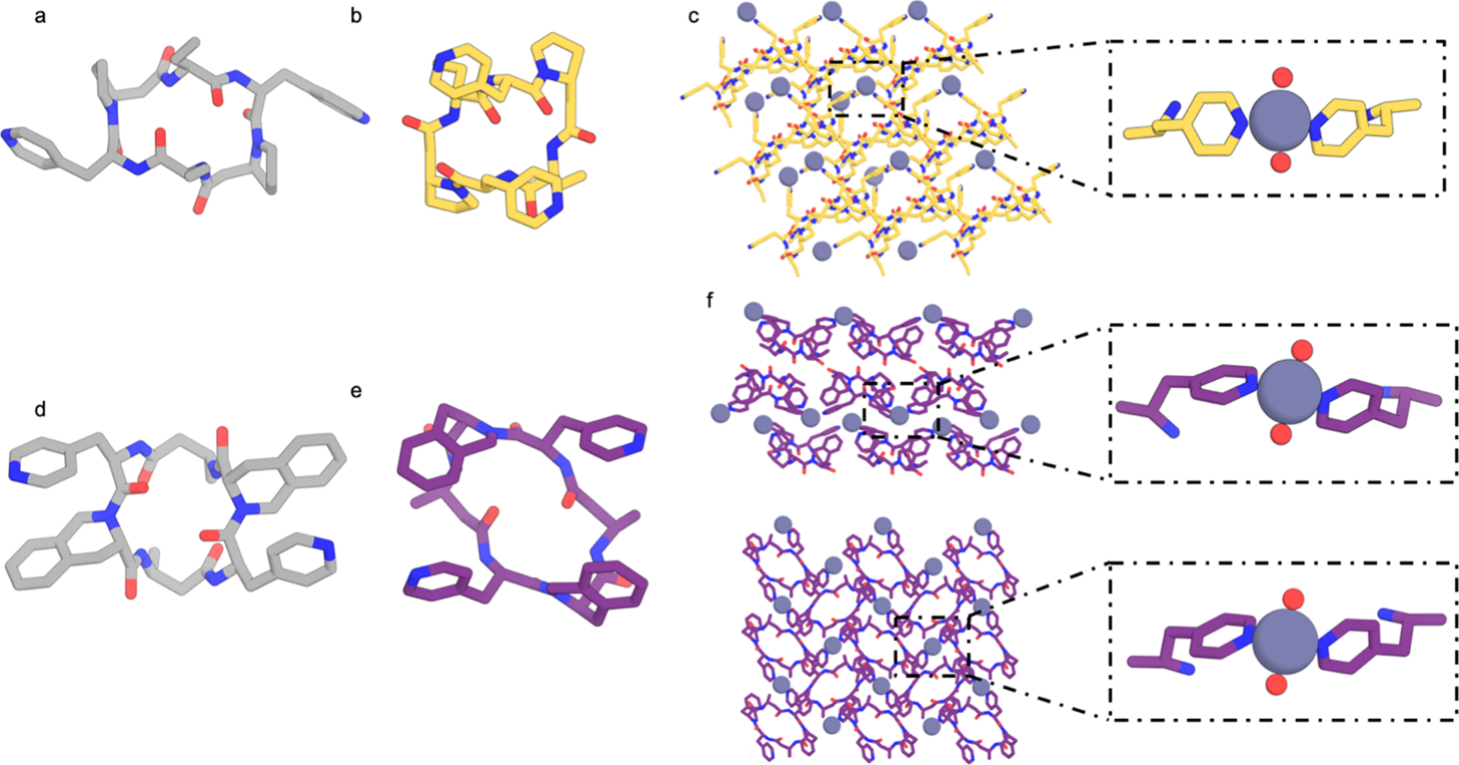
Structures of lattices formed by C2 pyridine-containing peptides. (a) Computational model of C2-1 [3-(4-pyridyl)-alanine−β-homoproline−α-aminobutyric acid–3-(4-pyridyl)-alanine−β-homoproline−α-aminobutyric acid] ligand. (b) Crystal structure of the C2-1 ligand. (c) Crystal structure of the peptide in the *P*1 space group. Inset view of the Zn^2+^-C2-1 1D tetrahedral coordination using two pyridines and two waters. (d) Computational model of C2-2 [3-(4-pyridyl)-alanine–1,2,3,4-tetrahydroisoquinoline-3-carboxylic acid–3-aminobutanoic acid–3-(4-pyridyl)-alanine–1,2,3,4-tetrahydroisoquinoline-3-carboxylic acid–3-aminobutanoic acid] ligand. (e) Crystal structure of the C2-2 ligand. (f) Crystal structure of C2-2 in the *P*12_1_1 space group (top). Inset shows a zoomed view of the Zn^2+^-C2-1 tetrahedral coordination. The crystal structure of peptide C2-2 in the *C*121 space group is shown on the bottom. Inset is a zoomed view of the Zn^2+^-C2-1 coordination.

To reduce the chance of backbone conformational changes and to explore a broader range of geometries and metal coordination ligands, we used a geometric hashing approach to design two pyridine-containing 6-mer peptides with AIMNet ground states having C2 symmetry (Figure S1a,b) and were able to obtain crystals with metal in multiple conditions after heating at 80 °C for 2 days. The structures of the crystals formed with 1 equivalent Zn(NO_3_)_2_ are shown in Figure 4. C2-1 (Figure 4a) formed crystals in the presence of HEPES pH 7.5 in the *P*1 (Figure 4c) space group, and C2-2 (Figure 4d) formed crystals in both the *C*121/*P*12_1_1 space groups (Figure 4f). In both cases, crystallization was driven by Zn-pyridine interactions, which formed 1D metal-peptide chains (Figure S8a,b) that hierarchically thread into 3D crystals.

In the lattice formed by the C2-1 ligand [3-(4-pyridyl)-alanine−β-homoproline−α-aminobutyric acid–3-(4-pyridyl)-alanine−β-homoproline−α-aminobutyric acid], each zinc ion is linked to two peptides through pyridine coordination, while two water molecules fill the other positions for full tetrahedral coordination (Figure 4c). The resulting peptide chains form 3D crystals through peptide stacking that is mediated by dispersion interactions and hydrogen bonding with participating water molecules. The 1D metal-peptide coordination chains grow along two different directions and intersect with each other, tiling the ab plane; non-covalent interactions mediate the stacking of these layers into 3D crystals. Water-filled pores between the coordination chains make up 25% of the calculated unit cell volume. The internal hydrogen bonds in the peptide design model (Figure 4a) are broken in the crystal (Figure 4b); AIMNet calculates the crystal conformation to be 4.7 kcal/mol higher in energy than the designed conformation, suggesting that the lattice stabilizes the higher energy state (Figure S1; we cannot exclude the posibility that the AIMNet calculations are incorrect, but given the large energy difference, and the low expected error (1.1 kcal/mol)^27^, crystal packing interactions are likely to distort the monomer ground state).

The crystal structure of peptide C2-2 (3-(4-pyridyl)-alanine–1,2,3,4-tetrahydroisoquinoline-3-carboxylic acid–3-aminobutanoic acid–3-(4-pyridyl)-alanine–1,2,3,4-tetrahydroisoquinoline-3-carboxylic acid–3-aminobutanoic acid) in the absence of metal in methanol matches that of the design model (Figure S2). However, crystal structures in the presence of Zn^2+^ reveal a different peptide conformation 3.4 kcal/mol higher than the designed conformation according to AIMNet (Figure 4d,e). The first crystal (*P*12_1_1 space group, Figure 4f-top) formed in HEPES pH 8.0 and 2% PEG2000 has a void volume of 35.3%. The second crystal (*C*121 space group, Figure 4f-bottom) formed in HEPES pH 8.0 and 2% PEP and has a void volume of 16%. As in the C2-1 case, in both crystals, the Zn^2+^ ions are tetrahedrally coordinated with two pyridine ligands and two water molecules and form 1D chains (Figure 4f), but the packing is slightly different. For the *C*121 crystal, 1D chains first arrange in a parallel fashion into bilayer planes, which then stack to form the 3D crystal lattice. The *P*12_1_1 crystal shares an identical peptide-zinc coordination configuration, but the 1D chains stack at different angles between the adjacent 2D planes. Thus, for this peptide, metal mediated interactions generate 1D chains, and higher order structures such as 2D planes and 3D crystals are stabilized by dispersion interactions through extensive interchain peptide–peptide packing.

We next explored metal-mediated crystals built from achiral S2 symmetric peptides. These peptides have a two-fold improper rotation across their axis of symmetry, allowing access to centrosymmetric space groups, which increases the likelihood of crystallization.^42^ Crystal structures determined in the absence of metal are very close to the design models.^25^

**Figure 5:**
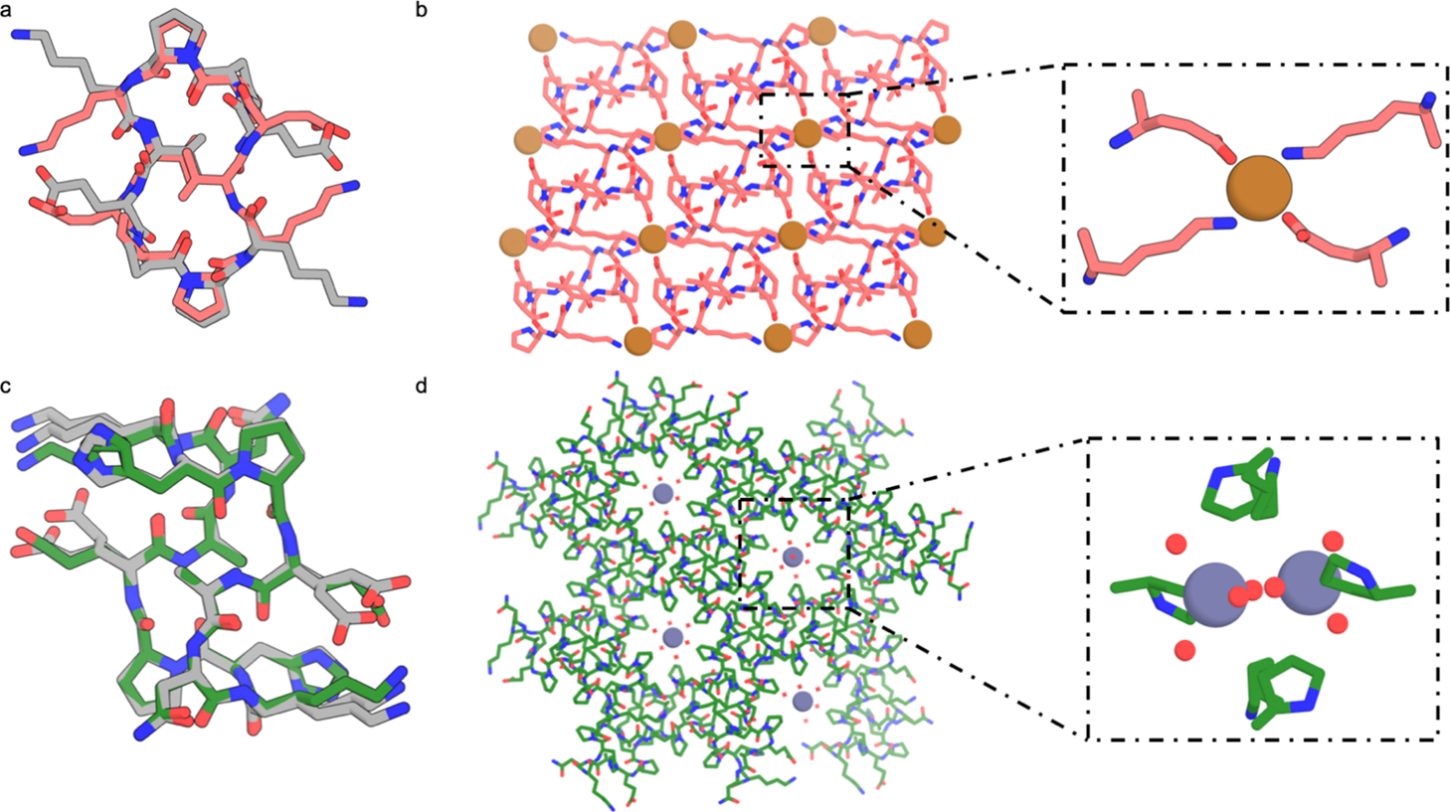
Lattices formed by cyclic peptides with S2 symmetry. (a) Superposition of S2-1 (ppKvEPPkVe) peptide monomer structure in the original apo crystal (gray) and in metal coordinating crystal (pink); the two are very similar to each other with a 0.53 Å Cα RMSD, and are also similar to the monomer computational design model^25^. (b) Crystal lattice of peptide S2-1 in *P*1̅ space group. Inset shows a single Cu^2+^ ion coordinated with two peptides via two lysines and two glutamates in a square planar geometry. (c) Superposition of S2-2 (aNkhPeAnKHpE) peptide monomer structure in the apo crystal (gray) and in metal-containing crystal (green); the two are again very similar to each other with a 0.43 Å Cα RMSD and are similar to the computational design model.^25^ (d) Crystal structure of peptide S2-2 in the *R*3 space group. Inset shows two zinc atoms coordinated by water molecules.

S2-1(ppKvEPPkVe), is a 10 residue S2 symmetric cyclic peptide containing one lysine and one glutamate per asymmetric unit (Figure 5a). The apo structure matches the design to 0.53 Å RMSD. S2-1 formed crystals upon heating at 80 °C for 24 h in DMF with one equivalent of Cu(NO_3_)_2_ in the *P*1̅ space group (Figure 5b) with very small pores making up 7% of the unit cell volume. A single Cu^2+^ is coordinated between two peptides via two lysines and two glutamates in a square planar geometry (Figure 5b). Each peptide forms a bidentate interaction with two Cu^2+^ ions that assemble into a crystal through peptide backbone hydrogen bonding. Despite the copper coordination, the peptide backbone conformation matches that of the design and the apo structure. The crystal lattice also matches that of the apo crystal with an expansion of a and b axis by 1 Å each to allow for metal incorporation into the structure.^25^

The 12 residue S2-2 peptide (aNkhPeAnKHpE, Figure 5c) contains one lysine, one histidine, and one glutamic acid per asymmetric unit available for metal coordination. The apo structure of this peptide matches the design to 0.43 Å RMSD.^25^ In the presence of 1 equivalent of ZnCl_2_, S2-2 crystallizes in isopropanol at room temperature in the *R*3 space group (Figure 5d). In the crystal structure, peptide–peptide interactions mediate crystal packing, while the large open channels along the c axis make up 40% of the unit cell volume (Figure S3f), which are filled with water-coordinated Zn^2+^ ions (Figure 5d). A comparison of the structure of S2-2 obtained in the absence and presence of metals indicates that the addition of Zn^2+^ did not change the overall crystal packing, since the Zn^2+^ ions occupied empty open channels in the crystal and are not coordinated to any of the aforementioned metal-binding residues. The peptide–peptide interactions in this crystal lattice are evidently more favorable than the metal coordination in the crystal conditions screened.

## Discussion

Computational design provides a stringent test of the understanding of a physical or biological system: one formulates a set of hypotheses, implements a computational method based on these hypotheses, uses the method to design new molecular structures, and determines whether the experimental structures match the computational designs. Discrepancies between the computations and experimental data can then guide increases in understanding of the systems. What can we learn from the discrepancies between the designs and the experiment observed here? While we designed and sought to crystallize 48 designs in the *I*2_1_3, *P*23, *P*4_1_32, and *P*4_3_32 space groups, only one crystallized and was in a different space group from the design. We cannot exclude the possibility that we did not find the appropriate crystallization conditions for the designs. But the simplest explanation, supported by the set of crystal structures we were able to obtain in our subsequent broad exploration of cyclic peptide ligands, is that the assumptions underlying our MOF design approach do not generally hold. First, we assumed that the designed peptide backbone conformation, in many cases supported by previous metal-free crystal structures, would be maintained in the metal-mediated crystals. While this was true for some peptides, many adopted likely higher energy conformations stabilized by metal and dispersion crystal packing interactions. Second, we assumed that, as with smaller MOF ligands, the peptides would fully coordinate the metals with amino acid sidechains and that this coordination would drive the assembly into the crystal. Instead, we observed consistent partial coordination of the metals with water and direct peptide–peptide mediated crystal packing interactions. This likely occurs because our ligands are much larger than the aromatic small molecules commonly used in MOF synthesis and can pack against each other using multiple dispersion and hydrogen bonding interactions, which can outweigh purely metal-mediated interactions. Full metal coordination by peptide groups, while on its own more favorable than water coordination of the metals, is out-competed by dispersion and hydrogen bonding interactions between these large peptides in the crystal lattice. These observations suggest that for the successful design of cyclic peptide-mediated MOFs, it will be necessary to relax the fixed backbone assumption and allow backbone sampling along with rigid body and sidechain sampling, perhaps using approaches similar to the RIF docking approach used for designing protein–protein interactions.^43^ It will also be necessary to sample a wider range of different crystal packing arrangements, both those involving peptide-metal coordination and those in which the primary crystal interactions are between the peptides, to more accurately determine whether the designed MOF is indeed the thermodynamically favorable structure. Development of improved computational design methods along these lines should enable a much more accurate design of macrocyclic-based MOFs, which could have a wide variety of applications.

## Conclusions

While MOFs have been previously described using short linear peptides, larger peptide ligands with internal symmetry have not to our knowledge, been previously explored. We report the first structures of symmetric cyclic 6 to 12 residue peptide MOFs with both proper and improper symmetries (C2, C3, and S2), employing metal-chelation histidine, cysteine, aspartate, glutamate, and noncanonical amino acids containing pyridine and DOPA side chains. Our crystal structures of six peptide MOFs with different metals (Zn^2+^, Co^2+^, and Cu^2+^) and space groups (*P*1, *P*65, *C*121, *P*12_1_1, *R*3, *P*4_1_2_1_2, and *P*1̅) contain a rich variety of 1D and 2D metal-mediated structures with pore shapes and sizes ranging from 7 to 40% void volume (some of these features have been observed in previous peptide-metal crystal structures, e.g., six residue poly-proline peptides can assemble into strings mediated by zinc and form dense frameworks through proline–proline packing).^17^ The up to 12 residue cyclic peptide ligands studied here are to our knowledge, the largest peptidic ligands reported that form crystalline coordination polymers to date. An essentially unlimited number of rigid symmetric cyclic peptides can be designed using the methods described in Mulligan et al.,^25^ and hence the crystal lattices described here are the first representatives of a very large class of new metal–organic crystals that could provide new peptide materials for biocompatible, chiral, and catalytic applications. The large surface area and pore sizes of these peptide-metal lattices make them particularly interesting for downstream applications such as catalysis and sensing, and the wide variety of both natural and unnatural sidechains available allows facile customization of the chemistry lining the pores and other structural features of the crystals. The lattices frequently contain open metal coordination sites (Figure S5), around which substrate binding pockets could be built by further computational design, providing access to a new class of catalytic materials combining the features of MOFs and enzymes.

## Supporting Information Available

Additional computational and experimental protocols including computational scripts, experimental observations, as well as analytical HPLC and LC–MS spectra for all compounds (PDF)
