# Supplementary material for: Exploration of Structured Symmetric Cyclic Peptides as Ligands for Metal-Organic Frameworks
Source: Chem Mater. 2022 Oct 25;34(21):9736–44. doi: 10.1021/acs.chemmater.2c02597 (PMC9648172; doi:10.1021/acs.chemmater.2c02597)

Supplementary Information for:

Exploration of Structured Symmetric Cyclic Peptides as ligands for Metal Organic Frameworks

Meerit Y. Said[1,2‡], Christine S. Kang[1,2‡], Shunzhi Wang[1,2‡], William Sheffler[1,2], Patrick J. Salveson[1,2], Asim K. Bera[1,2], Alex Kang[1,2], Hannah Nguyen[1,2], Ryanne Ballard[1,2], Xinting Li[1,2], Hua Bai[1,2], Lance Stewart[1,2], Paul Levine[1,2], David Baker[1,2,3]*

Table of Contents

Supplementary figures 2

Supplementary tables 5

Computational methods 11

Supplementary data 54

Supplementary figures

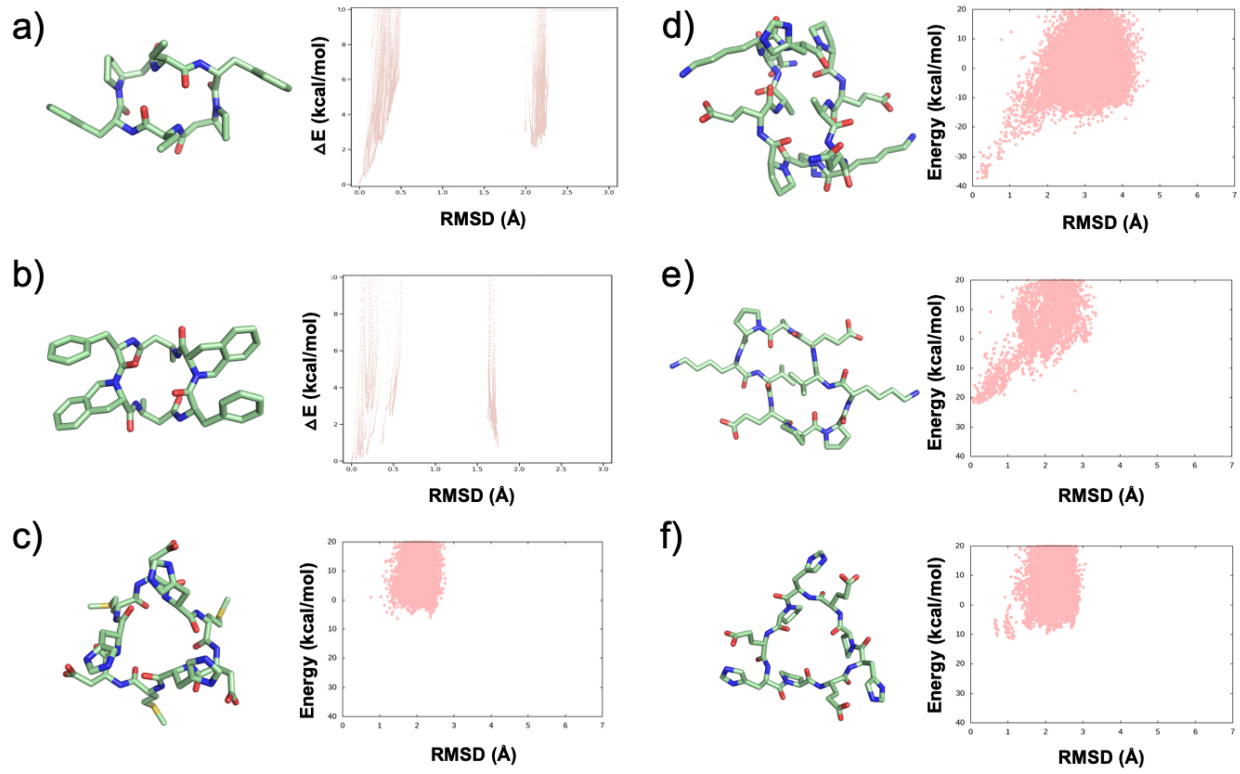

Figure S1. Predicted energy landscape for each designed peptide. The first column shows the designed conformation, while the second column shows the energy landscape calculated using either AIMNet (a-b) or Rosetta (c-f). Backbone RMSD is calculated to the designed conformation on the left. (a) AIMNet energy landscape for C2-1. (b) AIMNet energy landscape for C2-2. (c) Rosetta energy landscape for C3-2. (d) Rosetta energy landscape for S2-1. (e) Rosetta energy landscape for S2-2. (f) Rosetta energy landscape for C3-1.

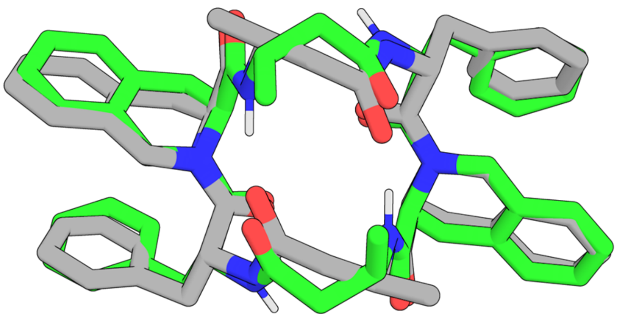

Figure S2. Alignment of C2-2 apo design (green) to crystal structure (gray) with 0.5 Å Cα RMSD.

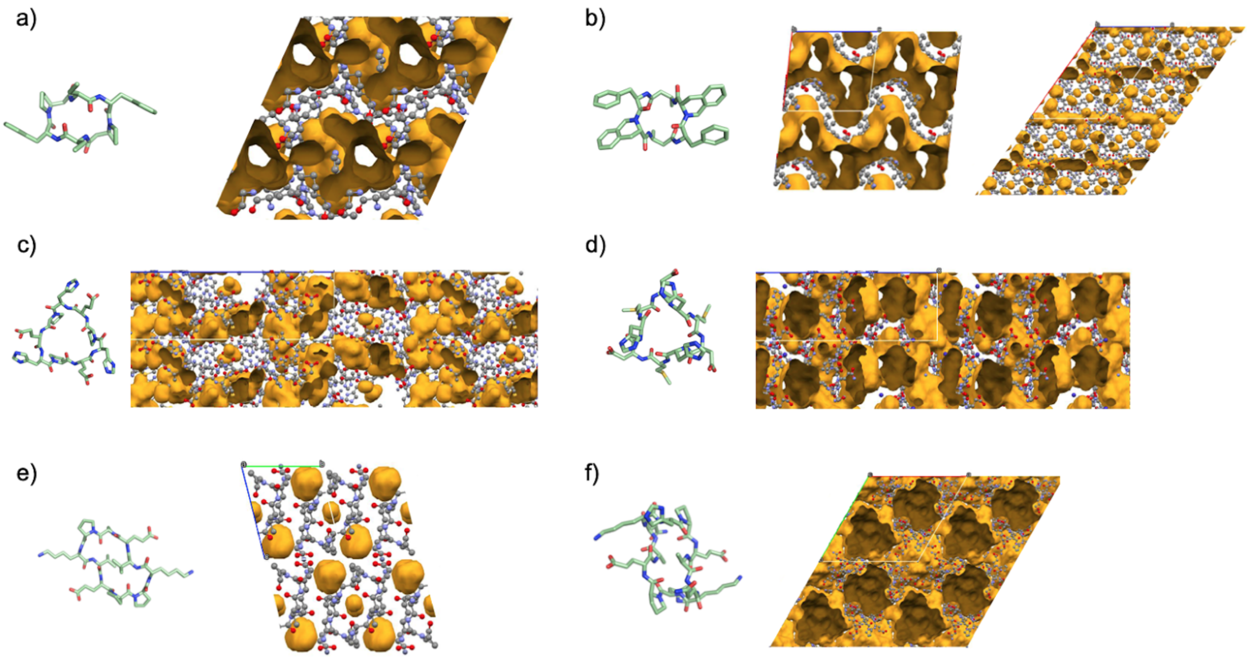

Figure S3. Mercury calculated void volume shown in yellow surface for each peptide crystal. (a) C2-1 crystal shown along the c axis. (b) C2-2 crystal 1 (left) and crystal 2 (right) shown along the b axis. (c) C3-1 crystal shown along the b axis. (d) C3-2 crystal shown along the b axis. (e) S2-1 crystal shown along the a axis. (f) S2-2 crystal shown along the c axis.

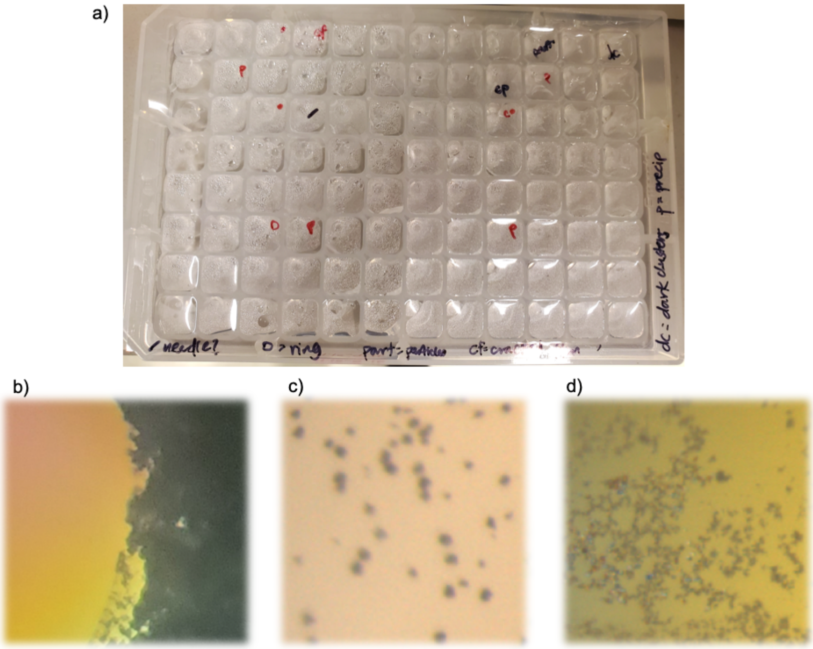

Figure S4. (a) Example of a 96 well plate used to screen one peptide. Wells that form precipitate are labeled with a 'P', dark particles are 'dc', crystalline materials are 'c'. (b) Example of a precipitated peptide. (c) Example of particles. (d) Example of crystalline peptide.

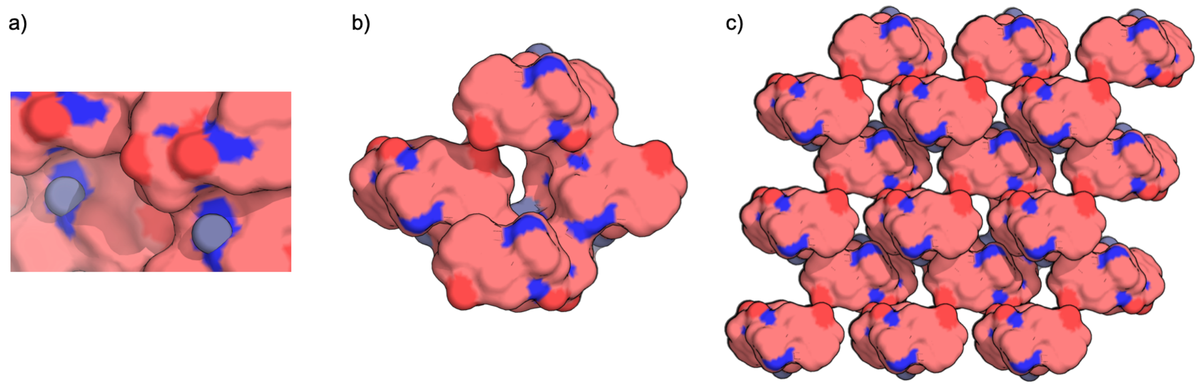

Figure S5. View along the b axis of C2-2 crystal. (a) Zoomed in view of the solvent accessible cavity with partially coordinated zinc ions (purple). (b) View of one pore going through the crystal lattice. (c) Multiple space units of C2-2 in the $P12_11$ space group.

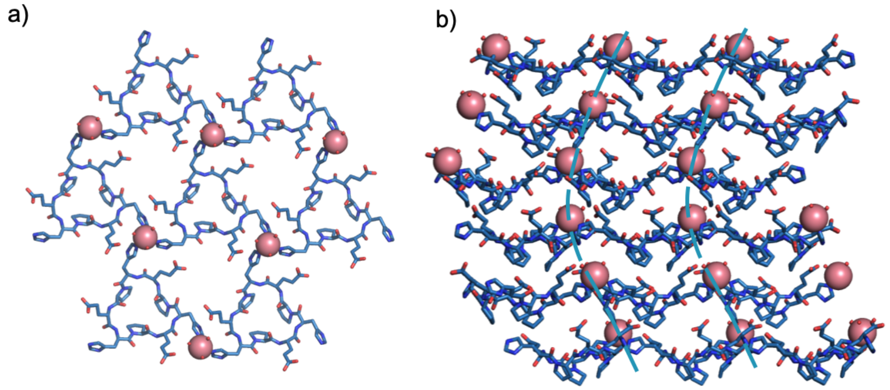

Figure S6. (a) View of a single layer of Co^{2+}-C3-1 2D sheet with C3 symmetry. (b) View along the *b* axis showing six layers of 2D planes stacked in a twisted way (teal dashed curves).

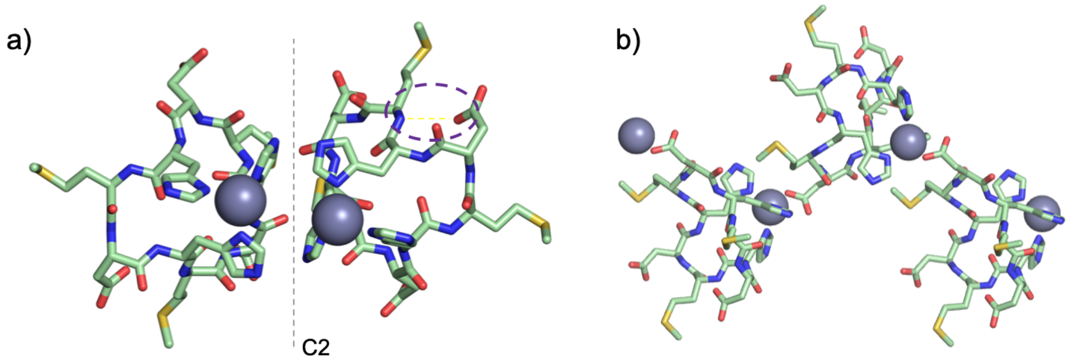

Figure S7. (a) Adjacent Zn-C3-2 1D chains interact via dispersion interactions. (b) 1D Metal coordinating peptide chain of C3-2.

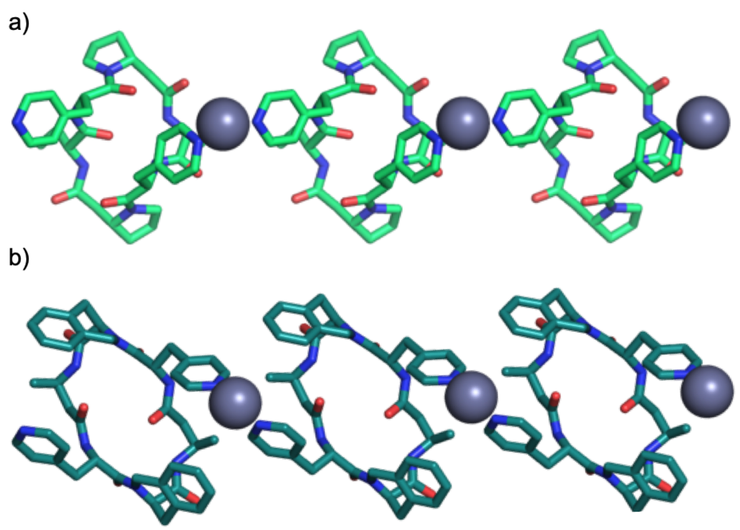

Figure S8. (a) view of the Zn^{2+}-C2-1 1D chain (two coordinated water molecules are omitted). (b) view of the Zn^{2+}-C2-2 1D chain (two coordinated water molecules are omitted).

Supplementary tables

Table S1: Organic conditions to screen for crystal formation using a 96 well plate.

	5mM $Zn(NO_3)_2$	5mM $Cu(NO_3)_2$	5mM $Zn(OAc)_2$	5mM $Cu(OAc)_2$	5mM $ZnCl_2$	5mM $CuCl_2$	5mM $Zn(NO_3)_2$	5mM $Cu(NO_3)_2$	5mM $Zn(OAc)_2$	5mM $Cu(OAc)_2$	5mM $ZnCl_2$	5mM $CuCl_2$
	1	2	3	4	5	6	7	8	9	10	11	12
A	DMF	DMF	DMF	DMF	DMF	DMF	MeOH	MeOH	MeOH	MeOH	MeOH	MeOH
B	1:1 water:DMF	1:1 water:DMF	1:1 water:DMF	1:1 water:DMF	1:1 water:DMF	1:1 water:DMF	1:1 water:MeOH	1:1 water:MeOH	1:1 water:MeOH	1:1 water:MeOH	1:1 water:MeOH	1:1 water:MeOH
C	2:1:1 DMF:water: ACN	2:1:1 DMF:water: ACN	2:1:1 DMF:water: ACN	2:1:1 DMF:water: ACN	2:1:1 DMF:water: ACN	2:1:1 DMF:water: ACN	2:1:1 MeOH:water :ACN	2:1:1 MeOH:water: ACN	2:1:1 MeOH:water: ACN	2:1:1 MeOH:water :ACN	2:1:1 MeOH:water :ACN	2:1:1 MeOH:water: ACN
D	1:2:1 DMF:water: ACN	1:2:1 DMF:water: ACN	1:2:1 DMF:water: ACN	1:2:1 DMF:water: ACN	1:2:1 DMF:water: ACN	1:2:1 DMF:water: ACN	1:2:1 MeOH:water :ACN	1:2:1 MeOH:water: ACN	1:2:1 MeOH:water: ACN	1:2:1 MeOH:water :ACN	1:2:1 MeOH:water :ACN	1:2:1 MeOH:water: ACN
E	DEF	DEF	DEF	DEF	DEF	DEF	EtOH	EtOH	EtOH	EtOH	EtOH	EtOH
F	2:1 DEF:water	2:1 DEF:water	2:1 DEF:water	2:1 DEF:water	2:1 DEF:water	2:1 DEF:water	1:1 water:EtOH	1:1 water:EtOH	1:1 water:EtOH	1:1 water:EtOH	1:1 water:EtOH	1:1 water:EtOH
G	1:1 water:DEF	1:1 water:DEF	1:1 water:DEF	1:1 water:DEF	1:1 water:DEF	1:1 water:DEF	IPA	IPA	IPA	IPA	IPA	IPA
H	1:2 DEF:water	1:2 DEF:water	1:2 DEF:water	1:2 DEF:water	1:2 DEF:water	1:2 DEF:water	1:1 water:IPA	1:1 water:IPA	1:1 water:IPA	1:1 water:IPA	1:1 water:IPA	1:1 water:IPA

Table S2: Aqueous conditions to screen for crystal formation using a 96 well plate.

	5mM $Zn(NO_3)_2$						5mM $Fe(NO_3)_3$					
	25mM MES pH 5.5	25 mM MES pH 6	25 mM MES pH 6.5	25 mM HEPES pH 7	25 mM HEPES pH 7.5	25 mM HEPES pH 8	25mM MES pH 5.5	25 mM MES pH 6	25 mM MES pH 6.5	25 mM HEPES pH 7	25 mM HEPES pH 7.5	25 mM HEPES pH 8
	1	2	3	4	5	6	7	8	9	10	11	12
A	-	-	-	-	-	-	-	-	-	-	-	-
B	PEG300 2%	PEG300 2%	PEG300 2%	PEG300 2%	PEG300 2%	PEG300 2%	PEG300 2%	PEG300 2%	PEG300 2%	PEG300 2%	PEG300 2%	PEG300 2%
C	PEG1000 2%	PEG1000 2%	PEG1000 2%	PEG1000 2%	PEG1000 2%	PEG1000 2%	PEG1000 2%	PEG1000 2%	PEG1000 2%	PEG1000 2%	PEG1000 2%	PEG1000 2%
D	PEG2000 2%	PEG2000 2%	PEG2000 2%	PEG2000 2%	PEG2000 2%	PEG2000 2%	PEG2000 2%	PEG2000 2%	PEG2000 2%	PEG2000 2%	PEG2000 2%	PEG2000 2%
E	PEG4000 2%	PEG4000 2%	PEG4000 2%	PEG4000 2%	PEG4000 2%	PEG4000 2%	PEG4000 2%	PEG4000 2%	PEG4000 2%	PEG4000 2%	PEG4000 2%	PEG4000 2%
F	PEP 2%	PEP 2%	PEP 2%	PEP 2%	PEP 2%	PEP 2%	PEP 2%	PEP 2%	PEP 2%	PEP 2%	PEP 2%	PEP 2%
G	EtOH 25%	EtOH 25%	EtOH 25%	EtOH 25%	EtOH 25%	EtOH 25%	EtOH 25%	EtOH 25%	EtOH 25%	EtOH 25%	EtOH 25%	EtOH 25%
H	MeOH 25%	MeOH 25%	MeOH 25%	MeOH 25%	MeOH 25%	MeOH 25%	MeOH 25%	MeOH 25%	MeOH 25%	MeOH 25%	MeOH 25%	MeOH 25%

Table S3: Observational notes for screened peptides.

peptide	sequence	precipitate	particles	crystalline
6.3_123_cell029	CYS DHIS PRO<br>CYS DHIS PRO<br>CYS DHIS PRO	DMF ($Zn(NO_3)_2$), MeOH ($Zn(NO_3)_2$), 1:4 water:DMF (($Zn(NO_3)_2$)),		
11.4_1240_cell028	GLU DHIS SER<br>GLU DHIS SER<br>GLU DHIS SER	DMF ($Zn(NO_3)_2$), MeOH ($Zn(NO_3)_2$), 1:4 water:DMF ($Zn(NO_3)_2$), H2O ($Zn(OAc)_2$), 100mM HEPES ($Cu(OAc)_2$)		
6.10_1223_cell028	CYS DHIS MET<br>CYS DHIS MET<br>CYS DHIS MET	1:1 water:MeOH ($Zn(NO_3)_2$, DMF ($Zn(OAc)_2$, $ZnCl_2$), 1:1 water:DMF ($Cu(OAc)_2$), DEF ($Zn(NO_3)_2$, $ZnCl_2$), 2:1 DEF:water ($Zn(NO_3)_2$), 1:1 water:DEF ($Cu(NO_3)_2$, $Cu(OAc)_2$), 1:2 DEF:water ($Cu(NO_3)_2$, $Cu(OAc)_2$)		
7.1_192_cell026	DCYS MET HIS<br>DCYS MET HIS<br>DCYS MET HIS	DMF ($Zn(NO_3)_2$, $Cu(NO_3)_2$), 1:4 water:DMF ($Zn(NO_3)_2$, $Cu(NO_3)_2$), 1:1 water:MeOH ($Zn(NO_3)_2$, $Cu(NO_3)_2$) DMF ($ZnCl_2$, $CuCl_2$), MeOH ($Zn(NO_3)_2$, $Cu(NO_3)_2$), 1:1 water:DMF ($ZnCl_2$, $CuCl_2$), DEF ($Zn(NO_3)_2$, $CuCl_2$), 2:1 DEF:water ($CuCl_2$), 1:1 water:DEF ($Cu(NO_3)_2$, $CuCl_2$), 1:2 DEF:water ($CuCl_2$)		
7.10_194_cell034	CYS PRO HIS<br>CYS PRO HIS<br>CYS PRO HIS	DMF ($Zn(NO_3)_2$, $Cu(NO_3)_2$), 1:4 water:DMF ($Zn(NO_3)_2$, $Cu(NO_3)_2$), MeOH ($Zn(OAc)_2$, $ZnCl_2$, $CuCl_2$), 1:1 water:MeOH ($ZnCl_2$), 2:1:1 DMF:water:ACN ($ZnCl_2$), 2:1:1 MeOH:water:ACN ($Cu(NO_3)_2$, $CuCl_2$), 1:2:1 DMF:water:ACN ($ZnCl_2$), 1:2:1 MeOH:water:ACN ($ZnCl_2$), EtOH ($Cu(NO_3)_2$, $Zn(OAc)_2$, $Cu(OAc)_2$, $ZnCl_2$, $CuCl_2$), 1:1 water:etOH ($ZnCl_2$, $CuCl_2$), 1:1 water:DEF ($Cu(OAc)_2$), IPA ($Zn(OAc)_2$, $Cu(OAc)_2$, $ZnCl_2$), 1:2 DEF:water ($Cu(OAc)_2$), 1:1 water:IPA ($Cu(NO_3)_2$, $Zn(OAc)_2$)		
1.3_176_cell037	ASP DHIS DMET<br>ASP DHIS DMET<br>ASP DHIS DMET	DMF ($Zn(NO_3)_2$, $Cu(NO_3)_2$, $ZnCl_2$), MeOH ($Zn(NO_3)_2$, $Cu(NO_3)_2$, $Zn(OAc)_2$), 1:1 water:DMF ($ZnCl_2$), 1:1 water:MeOH ($Zn(NO_3)_2$, $Cu(NO_3)_2$, $Zn(OAc)_2$), 2:1:1	1:1 water:DEF (Cu(NO3)$_2$, $Cu(OAc)_2$, $ZnCl_2$), IPA ($CuCl_2$), 1:2 DEF:water	DEF ($Zn(NO_3)_2$), 10mM MES pH 6 ($Zn(NO_3)_2$), 20mM HEPES pH 7

		DMF:water:ACN ($Cu(NO_3)_2$, $Zn(OAc)_2$, $ZnCl_2$, $CuCl_2$), 2:1:1 MeOH:water:ACN ($Zn(NO_3)_2$, $Cu(NO_3)_2$, $Zn(OAc)_2$), 1:2:1 DMF:water:ACN ($Cu(NO_3)_2$, $ZnCl_2$, $CuCl_2$), 1:2:1 MeOH:water:ACN ($Zn(NO_3)_2$, $Cu(NO_3)_2$, $Zn(OAc)_2$), DEF ($Zn(OAc)_2$, $CuCl_2$), EtOH ($Zn(NO_3)_2$, $Cu(NO_3)_2$, $CuCl_2$), 2:1 DEF:water ($Zn(OAc)_2$, $ZnCl_2$), 1:1 water:EtOH ($Zn(OAC)_2$, $Cu(NO_3)_2$, $Zn(NO_3)_2$), IPA ($Cu(OAc)_2$, $ZnCl_2$, $CuCl_2$), 1:2 DEF:water ($CuCl_2$), 1:1 water:IPA ($Zn(NO_3)_2$, $Cu(NO_3)_2$)	($Cu(NO3)_2$, $Cu(OAc)_2$, $ZnCl_2$)	($Zn(NO_3)_2$)
1.3_70_cell037	MET DHIS DASP<br>MET DHIS DASP<br>MET DHIS DASP	DMF($Zn(NO_3)_2$), 1:4 water:DMF ($Zn(NO_3)_2$), 1:1 water:MeOH ($Zn(NO_3)_2$), DMF ($ZnCl_2$), MeOH ($Zn(OAc)_2$), 1:1 water:DMF ($Zn(NO_3)_2$, $Cu(NO_3)_2$, $Zn(OAc)_2$), 2:1:1 DMF:water:ACN ($Zn(NO_3)_2$), DEF ($Zn(NO_3)_2$, $Zn(OAc)_2$, $Cu(OAc)_2$, $ZnCl_2$), 2:1 DEF:water ($Zn(NO_3)_2$, $Cu(NO_3)_2$, $Cu(OAc)_2$), 1:1 water:DEF ($CuCl_2$), IPA ($Cu(NO_3)_2$, $Zn(OAc)_2$, $Cu(OAc)_2$, $ZnCl_2$)		
12.6_637_cell047	GLU DHIS PRO<br>GLU DHIS PRO<br>GLU DHIS PRO	1:1 water:DMF ($Zn(OAc)_2$, $Cu(OAc)_2$, $Cu(NO_3)_2$), DMF ($Zn(OAc)_2$,		DMF ($Zn(NO_3)_2$), 20mM HEPES pH 7 ($Cu(NO_3)_2$, $Co(NO_3)_2$)), 20mM HEPES pH 8.2 ($Co(NO_3)_2$)
34.8_0_cell028	dHIS ALA ASP<br>SER dHIS ALA<br>ASP SER dHIS<br>ALA ASP SER	DMF ($Zn(NO_3)_2$, $Cu(OAc)_2$), 1:1 water:DMF ($Zn(NO_3)_2$, $Cu(OAc)_2$), DEF ($CU(OAc)_2$, $CuCl_2$), EtOH ($Cu(NO_3)_2$, $Cu(OAc)_2$), 2:1 DEF:water ($Zn(NO_3)_2$, $Cu(NO_3)_2$, $Zn(OAc)_2$, $Cu(OAc)_2$, IPA ($Cu(OAc)_2$)	1:1 water:DEF ($Zn(OAc)_2$)	1:1 water:DMF ($Zn(OAc)_2$)
25.8_19_cell031	MET DHIS PRO<br>DASP MET DHIS<br>PRO DASP MET<br>DHIS PRO DASP	DMF($Zn(OAc)_2$, $Cu(OAc)_2$), 1:1 water:DMF ($Zn(OAc)_2$, $Cu(OAc)_2$), 1:1 water:MeOH ($Zn(OAc)_2$, $Cu(OAc)_2$, 20mM HEPES pH 7 ($ZN(OAc)_2$, $Zn(NO_3)_2$, $Cu(NO_3)_2$, $Cu(OAc)_2$), 20mM HEPES pH 8 ($Zn(OAc)_2$, $Zn(NO_3)_2$, $Cu(OAc)_2$)		
7.7_120_cell034	DASP HIS MET<br>DASP HIS MET<br>DASP HIS MET	DMF ($Zn(OAc)_2$, $Cu(OAc)_2$, $ZnCl_2$), 1:1 water:DMF ($Zn(OAc)_2$), 1:1 water:MeOH ($Zn(NO_3)_2$), 2:1:1 DMF:water:ACN ($Zn(OAc)_2$), 2:1:1 MeOH:water:ACN ($Zn(NO_3)_2$), 1:2:1 DMF:water:ACN ($Zn(OAc)_2$), 1:2:1 MeOH:water:ACN ($Zn(NO_3)_2$), DEF ($Zn(NO_3)_2$, $ZnCl_2$), 2:1 DEF:water ($Zn(NO_3)_2$, $Zn(OAc)_2$), 1:1 water:EtOH ($Zn(NO_3)_2$, $Cu(NO_3)_2$), 1:1 water:DEF ($Zn(NO_3)_2$), IPA ($Cu(NO_3)_2$, $Cu(OAc)_2$, $CuCl_2$), 1:2 DEF:water ($Zn(OAc)_2$), 1:1 water:IPA ($Zn(NO_3)_2$, $Zn(OAc)_2$, $CuCl_2$)	2:1 DEF:water ($ZnCl_2$), DEF($Cu(OAc)_2$), 1:1 water:DEF ($Cu(OAc)_2$, $ZnCl_2$), 1:2 DEF:water ($Cu(OAc)_2$, $ZnCl_2$)	DMF ($Zn(NO_3)_2$, DEF ($Cu(NO_3)_2$)
3.4_196_cell030	DASP DGLN HIS<br>DASP DGLN HIS<br>DASP DGLN HIS	DMF ($Zn(NO_3)_2$, $CU(NO_3)_2$), MeOH ($Zn(NO_3)_2$, $Zn(OAc)_2$, $Cu(OAc)_2$, $ZnCl_2$, $CuCl_2$), 1:1 water:DMF ($Zn(NO_3)_2$, $Cu(NO_3)_2$, $Zn(OAc)_2$, $ZnCl_2$), 1:1 water:MeOH ($Zn(NO_3)_2$, $Zn(OAc)_2$, $Cu(NO_3)_2$, $Cu(OAc)_2$, $ZnCl_2$, $CuCl_2$), 2:1:1 DMF:water:ACN ($Zn(NO_3)_2$, $Zn(OAc)_2$, $Cu(NO_3)_2$, $Cu(OAc)_2$, $ZnCl_2$, $CuCl_2$), 2:1:1 MeOH:water:ACN ($Zn(NO_3)_2$, $Zn(OAc)_2$, $Cu(NO_3)_2$, $Cu(OAc)_2$, $ZnCl_2$, $CuCl_2$), 1:2:1 DMF:water:ACN ($Zn(NO_3)_2$, $Zn(OAc)_2$, $Cu(NO_3)_2$, $Cu(OAc)_2$), 1:2:1 MeOH:water:ACN ($Zn(OAc)_2$, $Cu(OAc)_2$, $ZnCl_2$, $CuCl_2$), DEF ($Zn(OAc)_2$, $Cu(NO_3)_2$, $Cu(OAc)_2$), DMF ($Zn(NO_3)_2$, $Zn(OAc)_2$, $Cu(OAc)_2$, $CuCl_2$), EtOH ($Zn(NO_3)_2$, $Zn(OAc)_2$, $Cu(NO_3)_2$, $Cu(OAc)_2$, $ZnCl_2$, $CuCl_2$), 2:1 DEF:water ($Zn(NO_3)_2$, $Zn(OAc)_2$, $Cu(NO_3)_2$, $Cu(OAc)_2$, $ZnCl_2$), 1:1 water:etOH ($Zn(NO_3)_2$, $Zn(OAc)_2$, $Cu(NO_3)_2$, $Cu(OAc)_2$, $ZnCl_2$), 1:1 water:DEF ($Zn(NO_3)_2$, $Zn(OAc)_2$, $Cu(NO_3)_2$, $Cu(OAc)_2$, $ZnCl_2$), IPA ($Zn(NO_3)_2$, $Zn(OAc)_2$, $Cu(OAc)_2$, $ZnCl_2$, $CuCl_2$), 1:2 DEF:water ($Zn(NO_3)_2$, $Zn(OAc)_2$, $Cu(NO_3)_2$, $Cu(OAc)_2$, $ZnCl_2$), 20mM HEPES pH7 ($Zn(OAc)_2$, $Zn(NO_3)_2$, $Cu(OAc)_2$, $Cu(NO_3)_2$), 20mM HEPES pH 8 ($Zn(OAc)_2$, $Zn(NO_3)_2$)	DMF ($Cu(NO_3)_2$), 1:1 MeOH:water ($Zn(NO_3)_{2)}$	
3.5_187_cell031	DASP DTRP HIS	DMF ($Zn(NO_3)_2$, $Zn(NO_3)_2$), MeOH ($Cu(NO_3)_2$), 1:1	MeOH ($Zn(NO_3)_2$, 1:1	

	DASP DTRP HIS DASP DTRP HIS	water:MeOH ($Cu(NO_3)_2$), 2:1:1 DMF:water:ACN ($CuCl_2$), 2:1:1 MeOH:water:ACN ($Cu(NO_3)_2$, $ZnCl_2$), 1:2:1 DMF:water:ACN ($Zn(NO_3)_2$, $Cu(NO_3)_2$, $Zn(OAc)_2$, $Cu(OAc)_2$, $CuCl_2$), 1:2:1 MeOH:water:ACN ($Zn(NO_3)_2$, $Cu(NO_3)_2$, $Zn(OAc)_2$), DEF ($Zn(NO_3)_2$, $Zn(OAc)_2$), EtOH ($Cu(NO_3)_2$, $Cu(OAc)_2$, $CuCl_2$), 2:1 DEF:water ($Zn(NO_3)_2$), 1:1 water:EtOH ($Zn(NO_3)_2$, $Zn(OAc)_2$, $Cu(NO_3)_2$, $Cu(OAc)_2$, $ZnCl_2$, $CuCl_2$), 1:1 water:DEF ($Cu(NO_3)_2$, $Zn(OAc)_2$), IPA ($Zn(NO_3)_2$, $Zn(OAc)_2$, $Cu(NO_3)_2$, $Cu(OAc)_2$, $ZnCl_2$, $CuCl_2$), 1:2 DEF:water ($Cu(NO_3)_2$, $Zn(OAc)_2$, $Cu(OAc)_2$, $ZnCl_2$, $CuCl_2$), 1:1 water:IPA ($Zn(NO_3)_2$, $Cu(NO_3)_2$, $Cu(OAc)_2$, $ZnCl_2$), 20mM HEPES pH 7 ($Zn(OAc)_2$, $Zn(NO_3)_2$, $Cu(NO_3)_2$, $Cu(OAc)_2$, $Co(NO_3)_2$), 20mM HEPES pH 8 ($Zn(OAc)_2$, $Zn(NO_3)_2$, $Cu(OAc)_2$, $Cu(NO_3)_2$, $FeCl_3$, $Co(NO_3)_2$)	water:MeOH ($CuCl_2$)	
28.6_2_cell032_peak 2	THR MET DCYS DHIS THR MET DCYS DHIS THR MET DCYS DHIS	MeOH ($Zn(OAc)_2$, 1:1 water:DMF ($Zn(NO_3)_2$, $Cu(NO_3)_2$, $ZnCl_2$), 2:1:1 DMF:water:ACN ($Cu(OAc)_2$, $ZnCl_2$), 2:1:1 DMF:water:ACN ($Zn(OAc)_2$), 1:2:1 DMF:water:ACN ($Cu(NO_3)_2$, $Cu(OAc)_2$, $ZnCl_2$), 1:2:1 MeOH:water:ACN ($Zn(NO_3)_2$, $CuCl_2$), DEF ($Zn(NO_3)_2$), EtOH ($Zn(NO_3)_2$, $Zn(OAc)_2$, $ZnCl_2$, $CuCl_2$), 1:1 water:EtOH ($Zn(NO_3)_2$, $ZnCl_2$), 1:1 water:DEF ($Zn(OAc)_2$), IPA ($Zn(NO_3)_2$, $Zn(OAc)_2$, $Cu(NO_3)_2$, $Cu(OAc)_2$, $ZnCl_2$, $CuCl_2$), 1:2 DEF:water ($Zn(NO_3)_2$, $Zn(OAc)_2$, $Cu(NO_3)_2$, $Cu(OAc)_2$, $ZnCl_2$), 1:1 water:IPA ($Zn(NO_3)_2$, $Zn(OAc)_2$, $CuCl_2$), 20mM HEPES pH 7 ($Zn(OAc)_2$, $Cu(OAc)_2$, $Cu(NO_3)_2$, $FeCl_3$, $Zn(NO_3)_2$,$CaCl_2$, $Co(NO_3)_2$), 20mM HEPES pH 8 ($Zn(NO_3)_2$, $Zn(OAc)_2$, $Cu(OAc)_2$, $Cu(NO_3)_2$, $FeCl_3$, $Co(NO_3)_2$, $CaCl_2$)	DMF ($CuCl_2$)	
34.3_0_cell032	MET DHIS DCYS PRO MET DHIS DCYS PRO MET DHIS DCYS PRO	MeOH ($CuNO_3)_2$), 1:! water:DMF ($Cu(NO_3)_2$, $CuCl_2$), 1:1 water:MeOH ($CuCl_2$), DEF ($Cu(OAc)_2$), 2:1 DEF:water ($Cu(OAc)_2$), 1:1 water:DEF ($Cu(NO_3)_2$, $Zn(OAc)_2$), IPA ($Zn(NO_3)_2$, $Zn(OAc)_2$, $Cu(NO_3)_2$, $Cu(OAc)_2$, $ZnCl_2$, $CuCl_2$), 1:2 DEF:water ($Zn(OAc)_2$, $Cu(OAc)_2$), 10mM MES pH 6 ($Zn(NO_3)_2$, $Cu(NO_3)_2$), 20mM HEPES pH 7 ($Zn(NO_3)_2$, $Zn(OAc)_2$, $Cu(NO_3)_2$, $Cu(OAc)_2$, $FeCl_3$), 20mM HEPES pH 8 ($Zn(NO_3)_2$, $Zn(OAc)_2$, $Cu(NO_3)_2$, $Cu(OAc)_2$, $FeCl_3$, $Co(NO_3)_2$)		
8.2_26_cell029	DCYS ALA PRO DASP DCYS ALA PRO DASP DCYS ALA PRO DASP	MeOH($Cu(OAc)_2$, $ZnCl_2$), 1:1 water:MeOH ($Cu(OAc)_2$), 2:1:1 MeOH:water:ACN ($ZnCl_2$), 1:2:1 DMF: water:ACN ($CuCl_2$), DEF ($Cu(NO_3)_2$), EtOH ($Cu(NO_3)_2$), 2:1 DEF:water ($Zn(NO_3)_2$), 1:1 water:EtOH ($Cu(OAc)_2$), IPA ($CuCl_2$), 1:1 water:IPA ($CuCl_2$)	2:1:1 DMF:water:ACN ($Cu(NO_3)_2$), 1:2:1 DMF:water:ACN ($Cu(NO_3)_2$), 1:1 water:DEF ($Cu(OAc)_2$), 1:2 DEF:water ($Cu(NO_3)_2$, $Cu(OAc)_2$), 1:1 water:IPA ($Cu(NO_3)_2$)	1:1 water:DMF ($Cu(OAc)_2$), 1:2:1 DMF:water:ACN ($Cu(OAc)_2$), 1:1 water:DEF ($Cu(NO_3)_2$)
1.3_470_cell032	HIS DASP DMET HIS DASP DMET HIS DASP DMET	DMF($Zn(NO_3)_2$, $Cu(NO_3)_2$, $Zn(OAc)_2$, $Cu(OAc)_2$), DEF ($Zn(NO_3)_2$), MeOH ($Zn(OAc)_2$, $Cu(OAc)_2$, $CuCl_2$), 1:1 water:DMF ($Zn(OAc)_2$), 1:1 water:MeOH ($Zn(NO_3)_2$, $Zn(OAc)_2$, $Cu(OAc)_2$), 2:1:1 DMF:water:ACN ($Zn(OAc)_2$), 2:1:1 MeOH:water:ACN ($Zn(OAc)_2$, $Cu(OAc)_2$), 1:2:1 MeOH:water:ACN ($Cu(NO_3)_2$, $Zn(OAc)_2$, $Cu(OAc)_2$), EtOH ($Zn(OAc)_2$, $Cu(OAc)_2$, $CuCl_2$), 2:1 DEF:water ($Zn(OAc)_2$, $Cu(OAc)_2$), 1:1 water:EtOH ($Zn(OAc)_2$, $Cu(OAc)_2$, $CuCl_2$), 1:1 water:DEF ($Cu(OAc)_2$, $CuCl_2$), IPA($Zn(OAc)_2$, $Cu(OAc)_2$), 1:2 DEF:water ($Cu(NO_3)_2$, $Zn(OAc)_2$, $CuCl_2$), 1:1 water:IPA ($Zn(OAc)_2$, $Cu(OAc)_2$)		
1.5_72_cell034	MET DASP CYS MET DASP CYS	DMF ($Zn(NO_3)_2$, $Cu(NO_3)_2$, $Zn(OAc)_2$, $Cu(OAc)_2$, $ZnCl_2$, $CuCl_2$), MeOH ($Zn(NO_3)_2$, $Cu(NO_3)_2$, $Zn(OAc)_2$, $Cu(OAc)_2$,		

	MET DASP CYS	$ZnCl_2$, $CuCl_2$), 1:1 water:DMF ($Zn(NO_3)_2$, $Cu(NO_3)_2$, $Zn(OAc)_2$, $Cu(OAc)_2$, $ZnCl_2$, $CuCl_2$), 1:1 water:MeOH ($Zn(NO_3)_2$, $Cu(NO_3)_2$, $Zn(OAc)_2$, $Cu(OAc)_2$, $ZnCl_2$, $CuCl_2$), 2:1:1 DMF:water:ACN ($Zn(NO_3)_2$, $Cu(NO_3)_2$, $Zn(OAc)_2$, $Cu(OAc)_2$, $ZnCl_2$, $CuCl_2$), 2:1:1 MeOH:water:ACN ($Zn(NO_3)_2$, $Cu(NO_3)_2$, $Zn(OAc)_2$, $Cu(OAc)_2$, $ZnCl_2$, $CuCl_2$), 1:2:1 DMF:water:ACN ($Zn(NO_3)_2$, $Cu(NO_3)_2$, $Zn(OAc)_2$, $Cu(OAc)_2$, $ZnCl_2$, $CuCl_2$), 1:2:1 MeOH:water:ACN ($Zn(NO_3)_2$, $Cu(NO_3)_2$, $Zn(OAc)_2$, $Cu(OAc)_2$, $ZnCl_2$, $CuCl_2$), DEF ($Zn(NO_3)_2$, $Cu(NO_3)_2$, $Zn(OAc)_2$, $Cu(OAc)_2$, $ZnCl_2$, $CuCl_2$), EtOH ($Zn(NO_3)_2$, $Cu(NO_3)_2$, $Zn(OAc)_2$, $Cu(OAc)_2$, $ZnCl_2$, $CuCl_2$), 2:1 DEF:water ($Zn(NO_3)_2$, $Cu(NO_3)_2$, $Zn(OAc)_2$, $Cu(OAc)_2$, $ZnCl_2$, $CuCl_2$), 1:1 water:EtOH ($Zn(NO_3)_2$, $Cu(NO_3)_2$, $Zn(OAc)_2$, $Cu(OAc)_2$, $ZnCl_2$, $CuCl_2$), 1:1 water:DEF ($Zn(NO_3)_2$, $Cu(NO_3)_2$, $Zn(OAc)_2$, Cu(OAc)2, $ZnCl_2$, $CuCl_2$), IPA ($Zn(NO_3)_2$, $Cu(NO_3)_2$, $Zn(OAc)_2$, $Cu(OAc)_2$, $ZnCl_2$, $CuCl_2$), 1:2 DEF:water ($Zn(NO_3)_2$, $Cu(NO_3)_2$, $Zn(OAc)_2$, $Cu(OAc)_2$, $ZnCl_2$, $CuCl_2$), 1:1 water:IPA ($Zn(NO_3)_2$, $Cu(NO_3)_2$, $Zn(OAc)_2$, $Cu(OAc)_2$, $ZnCl_2$, $CuCl_2$)		
10.1_32_cell030	HIS DVAL DASP HIS DVAL DASP HIS DVAL DASP	DMF ($Zn(NO_3)_2$, $Cu(NO_3)_2$, $Zn(OAc)_2$), MeOH ($Zn(NO_3)_2$, $Cu(NO_3)_2$, $Cu(OAc)_2$, $ZnCl_2$, $CuCl_2$), 1:1 water:DMF ($Zn(NO_3)_2$, $Cu(NO_3)_2$), 1:1 water:MeOH ($Zn(NO_3)_2$, $ZnCl_2$), 2:1:1 DMF:water:ACN ($Zn(NO_3)_2$, $Cu(NO_3)_2$), 2:1:1 MeOH:water:ACN ($Zn(NO_3)_2$, $CuCl_2$), 1:2:1 DMF:water:ACN ($CuCl_2$), 1:2:1 MeOH:water:ACN ($Zn(NO_3)_2$), DEF ($Cu(NO_3)_2$, $Zn(OAc)_2$), EtOH($Zn(NO_3)_2$, $Cu(NO_3)_2$), 2:1 DEF:water ($Zn(NO_3)_2$, $Cu(NO_3)_2$, $Zn(OAc)_2$), 1:1 water:DEF ($Zn(NO_3)_2$, $Cu(NO_3)_2$, $Zn(OAc)_2$), 1:2 DEF:water ($Zn(NO_3)_2$, $Cu(NO_3)_2$, $Zn(OAc)_2$), 1:1 water:IPA ($Zn(NO_3)_2$, $Cu(NO_3)_2$, $ZnCl_2$)		1:2:1 DMF:water:ACN ($Zn(NO_3)_2$, $Cu(OAc)_2$), DEF ($Zn(NO_3)_2$), 20mM HEPES pH 8 ($Co(NO_3)_2$)
10.5_9_I213_199	DASP PRO DASP DASP PRO DASP DASP PRO DASP	1:2 water:DEF ($Zn(OAc)_2$)	MeOH ($Cu(OAc)_2$, $CuCl_2$), 1:1 Water:DMF ($Cu(OAc)_2$, $ZnCl_2$),	
11.10_9_I213_16	ASP DPRO ASP ASP DPRO ASP ASP DPRO ASP	1:1 water:DMF ($Cu(NO_3)_2$), 1:1 water:MeOH ($CuOAc)_2$, 2:1 DEF:water ($Cu(OAc)_2$, 1:1 water:EtOH ($Zn(OAc)_2$), 29mM HEPES pH 7 ($FeCl_3$)	MeOH($Cu(OAc)_2$, $CuCl_2$)	2:1:1 DMF:water:ACN ($Cu(OAc)_2$)
2.2_9_I213_166	DASP DASP TYR DASP DASP TYR DASP DASP TYR	DMF ($Zn(OAc)_2$, $Cu(OAc)_2$), 20mM HEPES pH 8 ($FeCl_3$)	MeOH ($Cu(OAc)_2$, $Cu(NO_3)_2$), EtOH ($Cu(NO_3)_2$, $Cu(OAc)_2$)	DMF($CuCl_2$), MeOH ($ZnCl_2$), 2:1:1 DMF:water:ACN ($Zn(OAc)_2$, 1:2:1 DMF:water:ACN ($Zn(NO_3)_2$),
3.9_9_I213_260	VAL DASP DASP VAL DASP DASP VAL DASP DASP		MeOH($Cu(NO_3)_2$, $Cu(OAc)_2$), 1:1 water:MeOH ($Cu(NO_3)_2$),	
1.4_9_I213_51	ASP ASP DTYR ASP ASP DTYR ASP ASP DTYR	DMF ($ZnCl_2$)	DMF ($Cu(NO_3)_2$, $ZnCl_2$), MeOH ($Cu(NO_3)_2$, $Cu(OAc)_2$), 1:1 water:DMF ($CuNO_3)_2$), 1:1 water:MeOH ($Cu(NO_3)_2$, $Cu(OAc)_2$), 2:1:1 MeOH:water:ACN ($Zn(OAc)_2$), 1:2:1 DMF:water:ACN ($Zn(OAc)_2$), 1:2:1 MeOH:water:ACN ($Cu(OAc)_2$), DEF($Zn(OAc)_2$, EtOH ($Cu(OAc)_2$)	2:1:1 MeOH:water:ACN ($Zn(NO_3)_2$), 1:2:1 MeOH:water:ACN ($Cu(NO_3)_2$), EtOH ($Cu(NO_3)_2$)
3.9_9_I213_134	SER DASP DASP		MeOH ($Cu(NO_3)_2$,	

	SER DASP DASP SER DASP DASP		$Cu(OAc)_2$), 1:1 Water:MeOH ($Cu(NO_3)_2$),	
17.2_12_P4132_134	GLU DPRO GLU DASN GLU DPRO GLU DASN GLU DPRO GLU DASN	1:1 water:DMF ($Zn(NO_3)_2$, $Cu(NO_3)_2$, $ZnCl_2$, $CuCl_2$), 1:2:1 DMF:water:ACN ($Cu(NO_3)_2$), MeOH ($Cu(NO_3)_2$, $Cu(OAc)_2$), 1:1 water:MeOH ($Cu(OAc)_2$)	2:1 DEF:water ($CuCl_2$), 1:1 water:DEF ($Cu(OAc)_2$), DMF ($CaCl_2$), 1:1 water:MeOH ($Cu(NO_3)_2$)	DMF ($Cu(OAc)_2$), MeOH ($Cu(OAc)_2$), MeOH ($Cu(NO_3)_2$), 1:1 water:MeOH ($Cu(NO_3)_2$)
64.10_12_P4132_2659	SER GLU PRO GLU SER GLU PRO GLU SER GLU PRO GLU		DMF ($Cu(NO_3)_2$), 1:1 water:DMF ($Cu(NO_3)_2$), 2:1:1 DMF:water:ACN ($Cu(NO_3)_2$), 1:2:1 DMF:water:ACN ($Cu(NO_3)_2$), DEF ($Cu(NO_3)_2$), 1:1 water:DEF ($Cu(NO_3)_2$), 1:2 DEF:water ($Cu(NO_3)_2$, $Cu(OAc)_2$)	
28.1_12_P4132_164	ASP ASP DMET DPRO ASP ASP DMET DPRO ASP ASP DMET DPRO		MeOH ($Cu(OAc)_2$), 1:2 DEF:H2O ($Cu(OAc)_2$)	
90.1_12_I213_1099	DASP PRO DASP SER DASP PRO DASP SER DASP PRO DASP SER		DMF ($Zn(NO_3)_2$, $Cu(NO_3)_2$), 1:1 water:DMF ($Cu(NO_3)_2$), 1:2:1 DMF:water:ACN ($Cu(NO_3)_2$, $Cu(OAc)_2$), 1:2:1 MeOH:water:ACN ($Cu(NO_3)_2$, $Cu(OAc)_2$), DEF ($Cu(OAc)_2$), EtOH ($Cu(OAc)_2$), 2:1 DEF:water ($Cu(OAc)_2$), 1:1 water:DEF ($Cu(OAc)_2$), IPA ($Cu(NO_3)_2$), 1:2 DEF:water ($Cu(NO_3)_2$),	
34.4_12_I213_499	ASP DTYR ASP DPRO ASP DTYR ASP DPRO ASP DTYR ASP DPRO		MeOH ($Cu(NO_3)_2$), MeOH ($Cu(OAc)_2$)	
11.3_20_3_1in	dASP MET GLN dASP MET GLN dASP MET GLN	20mM HEPES pH 7 and 8 ($Cu(OAc)_2$), 20mM HEPES pH 7 and 8 ($Cu(NO_3)_2$), 20mM HEPES pH 7 and 8 ($FeCl_3$), 20mM HEPES pH 8 ($Zn(NO_3)_2$)		
7.7_22_13	SER GLU dSER SER GLU dSER SER GLU dSER	20mM HEPES pH 8 ($Cu(OAc)_2$), 20mM HEPES pH 7 and 8 ($Cu(NO_3)_2$), 20mM HEPES pH 7 and 8 ($FeCl_3$), 20mM HEPES pH 8 (($Zn(NO_3)_2$))		
11.9_20_0	ASP dMET THR ASP dMET THR ASP dMET THR	20mM HEPES pH 8 ($Cu(OAc)_2$), 20mM HEPES pH 8 ($Cu(NO_3)_2$), 20mM HEPES pH 7 and 8 ($FeCl_3$)		
33.5_12_P4132_3404	GLU DMET LYS GLU GLU DMET LYS GLU GLU DMET LYS GLU			
10.10_12_I213_193	THR MET DASP DASP THR MET DASP DASP THR MET DASP DASP			
12.4_12_I213_261	ASP DMET DSER ASP ASP DMET DSER ASP ASP DMET DSER ASP			

38.10_12_I213_155	DASP THR DASP DVAL DASP THR DASP DVAL DASP THR DASP DVAL			
57.1_12_I213_234	GLU GLU DTHR DMET GLU GLU DTHR DMET GLU GLU DTHR DMET			
80.1_12_I213_270	DASP MET DSER DASP DASP MET DSER DASP DASP MET DSER DASP			
3.9_18_0	dASP dALA SER dASP dALA SER dASP dALA SER			
11.3_20_3	dASP MET THR dASP MET THR dASP MET THR			
2.1_20_39	MET dSER GLU MET dSER GLU MET dSER GLU			
10.7_21_58	MET dSER dGLU MET dSER dGLU MET dSER dGLU			
10.7_20_2	ASP dVAL dSER ASP dVAL dSER ASP dVAL dSER			
1.2_18_18	GLU MET dALA GLU MET dALA GLU MET dALA			

Computational Methods

All scripts for metal crystal lattice design are available in the following repository: https://github.com/willsheffler/mof/tree/master/mof and shown in the following listings.

Listing S1: The following script samples rotamers of metal chelating residues such as aspartate, histidine, cystine, and glutamate around different metal geometries.

```
import numpy as np, rpxdock as rp, copy, os, mof
from mof import util
from mof.pyrosetta_init import make_1res_pose, get_sfxn, rVec, xform_pose, Pose
from abc import ABC, abstractmethod
"""
CONCERNS:
how to handle multiple metal binding sides not covered by rotamers, as in GLU
how to handle CYS chi2, which is based on HG being free-ish to rotate
"""
```

```
def get_rotclouds(**kw):
   kw = rp.Bunch(kw)

   chiresl_asp1 = kw.chiresl_asp1 / kw.scale_number_of_rotamers
   chiresl_asp2 = kw.chiresl_asp2 / kw.scale_number_of_rotamers
   chiresl_cys1 = kw.chiresl_cys1 / kw.scale_number_of_rotamers
   chiresl_cys2 = kw.chiresl_cys2 / kw.scale_number_of_rotamers
   chiresl_his1 = kw.chiresl_his1 / kw.scale_number_of_rotamers
   chiresl_his2 = kw.chiresl_his2 / kw.scale_number_of_rotamers
   chiresl_glu1 = kw.chiresl_glu1 / kw.scale_number_of_rotamers
   chiresl_glu2 = kw.chiresl_glu2 / kw.scale_number_of_rotamers
   chiresl_glu3 = kw.chiresl_glu3 / kw.scale_number_of_rotamers

   os.makedirs(kw.rotcloud_cache, exist_ok=True)

   params = (kw.chiresl_his1, kw.chiresl_his2, kw.chiresl_cys1, kw.chiresl_cys2,
kw.chiresl_asp1,
             kw.chiresl_asp2, kw.chiresl_glu1, kw.chiresl_glu2, kw.chiresl_glu3,
kw.maxdun_cys,
             kw.maxdun_asp, kw.maxdun_glu, kw.maxdun_his,
kw.scale_number_of_rotamers)
   ident = mof.util.hash_str_to_int(str(params))

   cache_file = kw.rotcloud_cache + '/%i.pickle' % ident
   if os.path.exists(cache_file):
      lC, lD, lE, lH, lJ, dC, dD, dE, dH, dJ, lB = rp.util.load(cache_file)
   else:
      print('building rotamer clouds')
      chi_range = lambda resl: np.arange(-180, 180, resl)
      chi_asp = [chi_range(x) for x in (chiresl_asp1, chiresl_asp2)]
      chi_cys = [chi_range(x) for x in (chiresl_cys1, chiresl_cys2)]
      chi_his = [chi_range(x) for x in (chiresl_his1, chiresl_his2)]
      chi_glu = [chi_range(x) for x in (chiresl_glu1, chiresl_glu2, chiresl_glu3)]


      lC = mof.rotamer_cloud.RotCloudCysZN(grid=chi_cys, max_dun_score=4.0 * 1.5)
      lD = mof.rotamer_cloud.RotCloudAspZN(grid=chi_asp, max_dun_score=5.0 * 1.5)
      lE = mof.rotamer_cloud.RotCloudGluZN(grid=chi_glu, max_dun_score=5.0 * 1.5)
      lH = mof.rotamer_cloud.RotCloudHisZN(grid=chi_his, max_dun_score=5.0 * 1.5)
      lJ = mof.rotamer_cloud.RotCloudHisdZN(grid=chi_his, max_dun_score=5.0 * 1.5)
      dC = mof.rotamer_cloud.RotCloudDCysZN(grid=chi_cys, max_dun_score=4.0 * 1.5)
      dD = mof.rotamer_cloud.RotCloudDAspZN(grid=chi_asp, max_dun_score=5.0 * 1.5)
      dE = mof.rotamer_cloud.RotCloudDGluZN(grid=chi_glu, max_dun_score=5.0 * 1.5)
      dH = mof.rotamer_cloud.RotCloudDHisZN(grid=chi_his, max_dun_score=5.0 * 1.5)
      dJ = mof.rotamer_cloud.RotCloudDHisdZN(grid=chi_his, max_dun_score=5.0 * 1.5)

      lB = mof.rotamer_cloud.RotCloudBPY(grid=chi_his, max_dun_score=3.0)

      rp.util.dump([lC, lD, lE, lH, lJ, dC, dD, dE, dH, dJ, lB], cache_file)

   return dict(lC=lC, lD=lD, lE=lE, lH=lH, lJ=lJ, dC=dC, dD=dD, dE=dE, dH=dH, dJ=dJ,
lB=lB)

class RotamerCloud(ABC):
   """holds transforms for a set of rotamers positioned at the origin"""
   def __init__(
      self,
      amino_acid,
      rotchi=None,
      max_dun_score=4.0,
      grid=None,
   ):
```

```
      super(RotamerCloud, self).__init__()
      self.amino_acid = amino_acid
      sfxn_rotamer = get_sfxn('rotamer')
      pose = make_1res_pose(amino_acid)
      if rotchi is None:
         if grid is None:
            rotchi = util.get_rotamers(pose.residue(1))
            rotchi = np.array([list(x) for x in rotchi])
         else:
            mesh = np.meshgrid(*grid, indexing='ij')
            rotchi = np.stack(mesh, axis=len(mesh))
            rotchi = rotchi.reshape(-1, len(mesh))

      assert len(rotchi), 'no chi angles specified'
      self.original_rotchi = rotchi
      self.original_origin = _get_stub_1res(pose)
      xform_pose(pose, np.linalg.inv(self.original_origin))
      self.rotchi = list()
      self.rotbin = list()
      self.rotscore = list()
      self.frameidx = list()
      self.rotframes = list()
      for irot, chis in enumerate(rotchi):
         for ichi, chi in enumerate(chis):
            pose.set_chi(ichi + 1, 1, chi)
         dun = sfxn_rotamer(pose)
         if dun > max_dun_score: continue

         for iframe, frame in enumerate(self.get_effector_frame(pose.residue(1))):
            print(irot, iframe, chis)
            self.rotbin.append(irot)
            self.rotchi.append(chis)
            self.rotscore.append(dun)
            self.frameidx.append(iframe)
            self.rotframes.append(frame)

      assert self.rotbin, 'no chi angles pass dun cut'

      self.rotbin = np.array(self.rotbin)
      self.rotscore = np.array(self.rotscore)
      self.rotchi = np.stack(self.rotchi)
      self.frameidx = np.stack(self.frameidx)
      self.rotframes = np.stack(self.rotframes)

      print(
         f'created RotamerCloud {self.amino_acid} nrots:
{len(np.unique(self.rotbin))} nframes: {len(self.rotbin)}'
      )

   def make_pose1res(self):
      pose = make_1res_pose(self.amino_acid)
      xform_pose(pose, np.linalg.inv(self.original_origin))
      return pose

   def subset(self, which):
      new_one = copy.copy(self)
      new_one.rotchi = self.rotchi[which]
      new_one.rotbin = self.rotbin[which]
      new_one.rotscore = self.rotscore[which]
      new_one.rotframes = self.rotframes[which]
      return new_one
```

```
    @abstractmethod
    def get_effector_frame(self, residue):
        pass

    def dump_pdb(self, path=None, position=np.eye(4), which=None, append=False):
        if path is None: path = self.amino_acid + '.pdb'
        res = self.make_pose1res().residue(1)
        natm = res.natoms()
        F = rp.io.pdb_format_atom
        with open(path, 'a' if append else 'w') as out:
            loopey_doodle = enumerate(self.rotchi)
            if which is not None:
                loopey_doodle = ((which, self.rotchi[which]), )
            for irot, chis in loopey_doodle:
                out.write('MODEL %i\n' % irot)
                for ichi, chi in enumerate(chis):
                    res.set_chi(ichi + 1, chi)
                for ia in range(1, natm + 1):
                    xyz = res.xyz(ia)
                    xyz = position @ np.array([xyz[0], xyz[1], xyz[2], 1])
                    line = F(ia=ia, ir=1, an=res.atom_name(ia), rn=res.name3(), c='A',
xyz=xyz)
                    out.write(line)
                orig = self.rotframes[irot, :, 3]
                x = orig + 0.5 * self.rotframes[irot, :, 0]
                y = orig + 0.5 * self.rotframes[irot, :, 1]
                z = orig + 0.5 * self.rotframes[irot, :, 2]
                orig = position @ orig
                x = position @ x
                y = position @ y
                z = position @ z
                out.write(F(ia=natm + 1, ir=1, an='ORIG', rn='END', c='B', xyz=orig))
                out.write(F(ia=natm + 2, ir=1, an='XDIR', rn='END', c='B', xyz=x,
elem='O'))
                out.write(F(ia=natm + 3, ir=1, an='YDIR', rn='END', c='B', xyz=y,
elem='CL'))
                out.write(F(ia=natm + 4, ir=1, an='ZDIR', rn='END', c='B', xyz=z,
elem='N'))
                out.write('ENDMDL\n')

    def __len__(self):
        return len(self.rotchi)



class RotCloudHisZN(RotamerCloud):
    def __init__(self, *args, **kw):
        super().__init__('HIS', *args, **kw)

    def get_effector_frame(self, residue):
        cd = residue.xyz('CD2')
        ce = residue.xyz('CE1')
        ne = residue.xyz('NE2')

        zn = rVec(0, 0, 0)
        for i in range(3):
            zn[i] = ne[i] - (cd[i] + ce[i]) / 2
        zn.normalize()
        for i in range(3):
            zn[i] = ne[i] + 2.2 * zn[i]

        return [rp.motif.frames.stub_from_points(zn, ne, ce).squeeze()]
```

```
class RotCloudDHisZN(RotCloudHisZN):
   def __init__(self, *args, **kw):
      RotamerCloud.__init__(self, 'DHIS', *args, **kw)

class RotCloudHisdZN(RotamerCloud):
   def __init__(self, *args, **kw):
      super(RotCloudHisdZN, self).__init__('HIS_D', *args, **kw)

   def get_effector_frame(self, residue):
      cg = residue.xyz('CG')
      ce = residue.xyz('CE1')
      nd = residue.xyz('ND1')

      zn = rVec(0, 0, 0)
      for i in range(3):
         zn[i] = nd[i] - (cg[i] + ce[i]) / 2
      zn /= np.linalg.norm(zn)
      for i in range(3):
         zn[i] = nd[i] + 2.2 * zn[i]

      return [rp.motif.frames.stub_from_points(zn, nd, ce).squeeze()]

class RotCloudDHisdZN(RotCloudHisdZN):
   def __init__(self, *args, **kw):
      RotamerCloud.__init__(self, 'DHIS_D', *args, **kw)

class RotCloudCysZN(RotamerCloud):
   def __init__(self, *args, **kw):
      super(RotCloudCysZN, self).__init__('CYS', *args, **kw)

   def get_effector_frame(self, residue):
      hg = residue.xyz('HG')
      sg = residue.xyz('SG')
      cb = residue.xyz('CB')
      orig = (hg - sg).normalized()
      for i in range(3):
         orig[i] = orig[i] * 2.32 + sg[i]
      frame = rp.motif.frames.stub_from_points(orig, sg, cb).squeeze()
      return [frame]

class RotCloudBPY(RotamerCloud):
   def __init__(self, *args, **kw):
      RotamerCloud.__init__(self, 'BPY', *args, **kw)

   def get_effector_frame(self, residue):
      ne1 = residue.xyz('NE1')
      nn1 = residue.xyz('NN1')
      fe = residue.xyz('FE')

      x = rp.homog.align_vectors([1, 0, 0], [0, 1, 0], ne1 - fe, nn1 - fe)
      x[:3, 3] = fe.x, fe.y, fe.z
      x1 = x @ rp.homog.align_vector([1, -1, 1], [0, 0, 1])
      x2 = x @ rp.homog.align_vector([1, -1, -1], [0, 0, 1])


      return [x1]  #, x2]

class RotCloudDCysZN(RotCloudCysZN):
   def __init__(self, *args, **kw):
      RotamerCloud.__init__(self, 'DCYS', *args, **kw)
```

```
class RotCloudAspZN(RotamerCloud):
   def __init__(self, *args, **kw):
      super(RotCloudAspZN, self).__init__('ASP', *args, **kw)

   def get_effector_frame(self, residue):
      return _asp_glu_effectors(residue)

class RotCloudDAspZN(RotCloudAspZN):
   def __init__(self, *args, **kw):
      RotamerCloud.__init__(self, 'DASP', *args, **kw)

class RotCloudGluZN(RotamerCloud):
   def __init__(self, *args, **kw):
      super(RotCloudGluZN, self).__init__('GLU', *args, **kw)

   def get_effector_frame(self, residue):
      return _asp_glu_effectors(residue)

class RotCloudDGluZN(RotCloudGluZN):
   def __init__(self, *args, **kw):
      RotamerCloud.__init__(self, 'DGLU', *args, **kw)

def _asp_glu_effectors(residue):
   if residue.name() in ('ASP', 'DASP'):
      names = 'CG', 'OD1', 'OD2'
   elif residue.name() in ('GLU', 'DGLU'):
      names = 'CD', 'OE1', 'OE2'
   else:
      raise NotImplementedError

   c = np.array(residue.xyz(names[0])).reshape(1, 3)
   o1 = np.array(residue.xyz(names[1])).reshape(1, 3)
   o2 = np.array(residue.xyz(names[2])).reshape(1, 3)



   orig = (c - o2) / np.linalg.norm(c - o2)
   orig = o1 + orig * 2.1

   c = rp.homog.hpoint(c)
   o1 = rp.homog.hpoint(o1)
   o2 = rp.homog.hpoint(o2)
   orig = rp.homog.hpoint(orig)
   rotaxis = rp.homog.hcross(o1 - c, o2 - c)
   rot = rp.homog.hrot(rotaxis, 10, o1, degrees=True).squeeze()
   frames = list()
   for irot in range(13):
      frame = rp.motif.frames.stub_from_points(orig, o1, c).squeeze()
      frames.append(frame)
      orig = (rot @ orig.squeeze()).reshape(1, 4)
   return frames

def _get_stub_1res(pose):
   res = pose.residue(1)
   n = res.xyz('N')
   ca = res.xyz('CA')
   c = res.xyz('C')
   return rp.motif.frames.bb_stubs(
      np.array([[n[0], n[1], n[2]]]),
      np.array([[ca[0], ca[1], ca[2]]]),
      np.array([[c[0], c[1], c[2]]]),
   ).squeeze()
```

```
# for pickle test file compatibility... this is very lazy... should regen
RotamerCloudAspZN = RotCloudAspZN
RotamerCloudCysZN = RotCloudCysZN
RotamerCloudGluZN = RotCloudGluZN
RotamerCloudHisZN = RotCloudHisZN
```

Listing S2: We define geometric parameters for each space group such as the axes for the symmetry components.

```
import numpy as np, rpxdock as rp
from rpxdock import homog as hm
from mof.data import data_dir

class XtalSpec:
   pass

class XtalSpecCC(XtalSpec):
   def __init__(
      self,
      spacegroup,
      nfold1,
      axis1,
      orig1,
      nfold2,
      axis2,
      orig2,
      nsubs,
   ):

      self.spacegroup = spacegroup
      self.nfold1 = int(nfold1)
      self.sym1 = 'C%i' % nfold1
      self.axis1 = hm.hnormalized(hm.hvec(axis1))
      self.orig1 = hm.hpoint(orig1)
      self.nfold2 = int(nfold2)
      self.sym2 = 'C%i' % nfold2
      self.axis2 = hm.hnormalized(hm.hvec(axis2))
      if hm.angle(self.axis1, self.axis2) > np.pi / 2:
         self.axis2[:3] = -self.axis2[:3]
      assert 90 > hm.angle_degrees(self.axis1, self.axis2)
      self.orig2 = hm.hpoint(orig2)
      self.nsubs = nsubs
      self.dihedral = np.degrees(hm.angle(axis1, axis2))
      self.axis1d = None
      self.axis2d = None
      try:
         self.frames = rp.load(_frames_files[spacegroup])
      except:
         self.frames = None

class XtalSpecCD(XtalSpec):
   def __init__(
      self,
      spacegroup,
      nfold1,
      axis1,
```

```
      orig1,
      nfold2,
      axis2,
      orig2,
      axis2d,
      nsubs,
   ):
      self.spacegroup = spacegroup
      self.nfold1 = int(nfold1)
      self.sym1 = 'C%i' % nfold1
      self.axis1 = hm.hnormalized(hm.hvec(axis1))
      self.orig1 = hm.hpoint(orig1)
      self.nfold2 = int(nfold2)
      self.sym2 = 'C%i' % nfold2
      self.axis2 = hm.hnormalized(hm.hvec(axis2))
      if hm.angle(self.axis1, self.axis2) > np.pi / 2:
         self.axis2[:3] = -self.axis2[:3]
      assert 90 > hm.angle_degrees(self.axis1, self.axis2)

      self.orig2 = hm.hpoint(orig2)
      self.nsubs = nsubs
      self.dihedral = np.degrees(hm.angle(axis1, axis2))
      self.axis1d = None
      self.axis2d = hm.hnormalized(hm.hvec(axis2d))
      try:
         self.frames = rp.load(_frames_files[spacegroup])
      except:
         self.frames = None

_frames_files = {
   'I 21 3': data_dir + '/i213_redundant111_n16_maxrad2.pickle',
   'P 41 3 2': data_dir + '/p4132_trionly_n12_maxrad3.pickle',
   'P 43 3 2': data_dir + '/p4132_trionly_n12_maxrad3.pickle',
}

def get_xtal_spec(name):
   try:
      return _xspec[name.lower()]
   except KeyError:
      print(f'spacegroup {name} not implemented')


_xspec = dict(
   f432=XtalSpecCC(
      'F 4 3 2',
      3,
      [1, 1, 1],
      [0.5, 0.5, 0],
      4,
      [1, 0, 0],
      [0, 0, 0],
      24,
   ),
   p213=XtalSpecCC(
      'P 21 3',
      3,
      [+1, +1, +1],
      [0, 0, 0],
      3,
      [-1, +1, +1],
      [+0., +0, +0.5],
      12,
```

```
    ),
    i213=XtalSpecCC(
        'I 21 3',
        3,
        [1, 1, 1],
        [0, 0, 0],
        2,
        [0, 0, 1],
        [0, -0.25, 0],
        12,
    ),

    p4132=XtalSpecCC(
        'P 41 3 2',
        3,
        [-1, -1, 1],
        [-0.5, 0, -0.5],
        2,
        [0, -1, 1],
        [-0.125, -0.125, -0.125],
        24,
    ),
    p4332=XtalSpecCC(
        'P 43 3 2',
        3,
        [-1, -1, 1],
        [-0.5, 0, -0.5],
        2,
        [0, -1, 1],
        [-0.375, -0.125, -0.125],
        24,
    ),
    p23=XtalSpecCD(
        'P 2 3',
        3,
        [1, -1, 1],
        [-2 / 3, -1 / 3, 1 / 3],
        2,
        [0, 1, 0],
        [-1 / 2, -1 / 2, 0],
        [1, 0, 0],
        12,
    ))
```

Listing S3: We dock C3 peptides and C3 octahedral metal sites into space group $P2_13$.

```
import mof, os, numpy as np, rpxdock as rp, rpxdock.homog as hm
from mof.pyrosetta_init import (rosetta, makelattice, get_sfxn, xform_pose,
make_residue)
from mof.util import align_cx_pose_to_z, variant_remove
from pyrosetta.rosetta.numeric import xyzVector_double_t as xyzVec
from pyrosetta import AtomID, get_score_function

def main_loop():

    kw = mof.app.options_setup(get_test_kw, verbose=False)

    if kw.postprocess:
```

```
      return mof.app.postprocess(kw)

   pept_axis = np.array([0, 0, 1, 0])
   pept_orig = np.array([0, 0, 0, 1])

   results = list()
   rfname = f'{kw.output_prefix}results.pickle'
   print('fname prefix:', kw.output_prefix)
   print('result fname:', rfname)

   sfxn = get_score_function()

   results = list()

   for ipdbpath, pdbpath in enumerate(kw.inputs):

      pose = rosetta.core.import_pose.pose_from_file(pdbpath)
      if not align_cx_pose_to_z(pose, pdbpath):
         print(f"WARNING failed align_cx_pose_to_z: {pdbpath}")
      variant_remove(pose)
      rpxbody = rp.Body(pose)

      rotclouds = mof.rotamer_cloud.get_rotclouds(**kw)

      minscore = 9e9

      print()
      print(f'{" %i of %i "%(ipdbpath+1 , len(kw.inputs)):#^80}')
      print(pdbpath)
      print(f'{"":#^80}')

      for spacegroup in kw.spacegroups:

         search_spec = mof.xtal_search.XtalSearchSpec(
            spacegroup=spacegroup,
            pept_orig=np.array([0, 0, 0, 1]),
            pept_axis=np.array([0, 0, 1, 0]),
            # are these necessary:
            sym_of_ligand=dict(HZ3='C3', DHZ3='C3', HZ4='C4', DHZ4='C4', HZD='D2',
DHZD='D2',
                               BPY='C3'),
            ligands=['HZ3', 'DHZ3'],
            **kw,
         )
         xspec = search_spec.xtal_spec
         target_angle = hm.line_angle_degrees(xspec.axis1, xspec.axis2)

         sym_num = xspec.nfold1
         nresasym = len(pose.residues) // sym_num

         for iaa, aa in enumerate(kw.aa_labels):

            rotcloud = rotclouds[mof.app.lblmap[aa]]

            for ires in range(nresasym):

               print(f'{f" LOOP {spacegroup} {aa} {ires} ":*^80}')
               stub = rpxbody.stub[ires]

               rotframes = stub @ rotcloud.rotframes

               for irot, rotframe in enumerate(rotframes):
```

```
                  ligsymaxis = rotframe[:, 2]  # z axis

                  if np.pi / 2 < hm.angle(pept_axis, ligsymaxis):
                     ligsymaxis *= -1

                  angle = hm.angle_degrees(pept_axis, ligsymaxis)
                  if abs(angle - target_angle) > kw.angle_err_tolerance:
                     continue


                  orig_metal_pos = rotframe[:, 3]


                  xalign, delta = hm.align_lines_isect_axis2(
                     pept_orig, pept_axis, orig_metal_pos, ligsymaxis, xspec.axis1,
xspec.orig1,
                     xspec.axis2, xspec.orig2 - xspec.orig1, strict=False)


                  aligned_pept_axis = xalign @ pept_axis
                  aligned_ligsym_axis = xalign @ ligsymaxis
                  aligned_metal_pos = xalign @ orig_metal_pos
                  assert np.allclose(aligned_pept_axis, xspec.axis1,
atol=kw.angle_err_tolerance)
                  assert np.allclose(aligned_ligsym_axis, xspec.axis2,
                                     atol=kw.angle_err_tolerance)

                  _, isect = hm.line_line_closest_points_pa(aligned_metal_pos,
                                                            aligned_ligsym_axis, [0,
0, 0, 1],
                                                            xspec.orig2 -
xspec.orig1)
                  cell_spacing = abs(isect[2] / xspec.orig2[2])
                  if cell_spacing < 10 or cell_spacing > 25:
                     continue


                  outpose0 = mof.util.mutate_one_res(pose, ires + 1, aa,
rotcloud.rotchi[irot],
                                                     sym_num)
                  xform_pose(outpose0, xalign)
                  outasym = rosetta.protocols.grafting.return_region(outpose0, 1,
nresasym)
                  ci = rosetta.core.io.CrystInfo()
                  ci.A(cell_spacing)  # cell dimensions
                  ci.B(cell_spacing)
                  ci.C(cell_spacing)
                  ci.alpha(90)  # cell angles n
                  ci.beta(90)
                  ci.gamma(90)
                  ci.spacegroup(xspec.spacegroup)  # sace group
                  pi = rosetta.core.pose.PDBInfo(outasym)
                  pi.set_crystinfo(ci)
                  outasym.pdb_info(pi)

                  sympose = outasym.clone()
                  rosetta.protocols.cryst.MakeLatticeMover().apply(sympose)
                  conf = sympose.conformation().clone()
                  assert rosetta.core.conformation.symmetry.is_symmetric(conf)
                  syminfo = rosetta.core.pose.symmetry.symmetry_info(sympose)

                  nasym = outasym.size()
```

```
                  conf.declare_chemical_bond(nasym, 'C', 1, 'N')
                  sympose.set_new_conformation(conf)
                  sympose.set_new_energies_object(
                     rosetta.core.scoring.symmetry.SymmetricEnergies())

                  sc = sfxn.score(sympose)
                  minscore = min(minscore, sc)
                  if sc > 7000:
                     continue

                  syminfo = rosetta.core.pose.symmetry.symmetry_info(sympose)
                  surfvol = rosetta.core.scoring.packing.get_surf_vol(sympose, 1.4)
                  peptvol = 0.0
                  for ir in range(1, syminfo.get_nres_subunit() + 1):
                     res = sympose.residue(ir)
                     ir = ir + 1  # rosetta numbering
                     for ia in range(1, res.natoms() + 1):
                        v = surfvol.vol[AtomID(ia, ir)]
                        if not np.isnan(v): peptvol += v
                  peptvol *= xspec.nsubs
                  print(f'peptvol {peptvol} {peptvol / cell_spacing**3}')
                  solv_frac = max(0.0, 1.0 - peptvol / cell_spacing**3)

                  tag = ''.join([
                     f'_cell{int(cell_spacing):03}_',
                     f'_sc{int(sc):05}_',
                     f'_solv{int(solv_frac*100):02}_',
                     f'{os.path.basename(pdbpath)}',
                     f'_nres{nresasym}',
                     f'{aa}_',
                     f'{len(results):06}',
                  ])
                  fn = kw.output_prefix + tag + '.pdb'

                  print('HIT %7.3f' % sc, outasym)
                  results.append([sc, cell_spacing, sympose])
                  if True:
                     outasym.dump_pdb(fn)

      print('minscore', minscore)

   results.sort

   if not results:
      print(f'{"":!^100}')
      print('NO RESULTS!!!')
      print(f'{"":!^100}')
      print('DONE')

   return results

validated_c3 = [
   '<path_to_validated_peptides/peptide.pdb>',
]

def get_test_kw(kw):
   if not kw.inputs:
      kw.inputs = ['mof/data/peptides/c.2.6_0001.pdb']
      # kw.inputs = validated_c3
      print(f'{"":!^80}')
      print(f'{"no pdb list input, using test only_one":!^80}')
      print(f'{str(kw.inputs):!^80}')
```

```
      print(f'{"":!^80}')

   kw.spacegroups = ['p213']
   kw.aa_labels = ['BPY']
   kw.output_prefix = '_mof_test_c3c3' + '_'.join(kw.spacegroups) + '/'
   kw.angle_err_tolerance = 3
   kw.scale_number_of_rotamers = 0.25
   kw.max_bb_redundancy = 2.0
   kw.max_dun_score = 4.0
   kw.clash_dis = 3.3
   kw.contact_dis = 7.0
   kw.min_contacts = 0
   kw.max_score_minimized = 40.0
   kw.min_cell_size = 0
   kw.max_cell_size = 50
   kw.max_solv_frac = 0.80
   kw.debug = True
   # kw.continue_from_checkpoints = False
   return kw
```

Listing S4: We dock C3 peptides and D2 tetrahedral metal sites into *P*23 space group.

```
import mof, os, numpy as np, rpxdock as rp, rpxdock.homog as hm
from mof.pyrosetta_init import (rosetta, makelattice, get_sfxn, xform_pose,
make_residue)
from mof.util import align_cx_pose_to_z, variant_remove
from pyrosetta.rosetta.numeric import xyzVector_double_t as xyzVec
from pyrosetta import AtomID, get_score_function

def main_loop():

   kw = mof.app.options_setup(get_test_kw, verbose=False)

   if kw.postprocess:
      return mof.app.postprocess(kw)

   pept_axis = np.array([0, 0, 1, 0])
   pept_orig = np.array([0, 0, 0, 1])

   results = list()
   rfname = f'{kw.output_prefix}results.pickle'
   print('fname prefix:', kw.output_prefix)
   print('result fname:', rfname)

   sfxn = get_score_function()

   results = list()

   for ipdbpath, pdbpath in enumerate(kw.inputs):

      pose = rosetta.core.import_pose.pose_from_file(pdbpath)
      if not align_cx_pose_to_z(pose, pdbpath):
         print(f"WARNING failed align_cx_pose_to_z: {pdbpath}")
      variant_remove(pose)
      rpxbody = rp.Body(pose)

      rotclouds = mof.rotamer_cloud.get_rotclouds(**kw)
```

```
    minscore = 9e9

    print()
    print(f'{" %i of %i "%(ipdbpath+1 , len(kw.inputs)):#^80}')
    print(pdbpath)
    print(f'{"":#^80}')

    for spacegroup in kw.spacegroups:

       search_spec = mof.xtal_search.XtalSearchSpec(
          spacegroup=spacegroup,
          pept_orig=np.array([0, 0, 0, 1]),
          pept_axis=np.array([0, 0, 1, 0]),
          # are these necessary:
          sym_of_ligand=dict(HZ3='C3', DHZ3='C3', HZ4='C4', DHZ4='C4', HZD='D2',
DHZD='D2',
                             BPY='C3'),
          ligands=['HZ3', 'DHZ3'],
          **kw,
       )
       xspec = search_spec.xtal_spec
       target_angle = hm.line_angle_degrees(xspec.axis1, xspec.axis2)

       sym_num = xspec.nfold1
       nresasym = len(pose.residues) // sym_num

       for iaa, aa in enumerate(kw.aa_labels):

          rotcloud = rotclouds[mof.app.lblmap[aa]]

          for ires in range(nresasym):

             print(f'{f" LOOP {spacegroup} {aa} {ires} ":*^80}')
             stub = rpxbody.stub[ires]

             rotframes = stub @ rotcloud.rotframes

             for irot, rotframe in enumerate(rotframes):
                ligsymaxis = rotframe[:, 2]  # z axis

                if np.pi / 2 < hm.angle(pept_axis, ligsymaxis):
                   ligsymaxis *= -1

                angle = hm.angle_degrees(pept_axis, ligsymaxis)
                if abs(angle - target_angle) > kw.angle_err_tolerance:
                   continue


                xalign, delta = hm.align_lines_isect_axis2(
                   pept_orig, pept_axis, orig_metal_pos, ligsymaxis, xspec.axis1,
xspec.orig1,
                   xspec.axis2, xspec.orig2 - xspec.orig1, strict=False)

                aligned_pept_axis = xalign @ pept_axis
                aligned_ligsym_axis = xalign @ ligsymaxis
                aligned_metal_pos = xalign @ orig_metal_pos
                assert np.allclose(aligned_pept_axis, xspec.axis1,
atol=kw.angle_err_tolerance)
                assert np.allclose(aligned_ligsym_axis, xspec.axis2,
                                   atol=kw.angle_err_tolerance)

                _, isect = hm.line_line_closest_points_pa(aligned_metal_pos,
```

```
                                                           aligned_ligsym_axis, [0,
0, 0, 1],
                                                           xspec.orig2 -
xspec.orig1)
                  cell_spacing = abs(isect[2] / xspec.orig2[2])
                  if cell_spacing < 10 or cell_spacing > 25:
                     continue

                  outpose0 = mof.util.mutate_one_res(pose, ires + 1, aa,
rotcloud.rotchi[irot],
                                                    sym_num)
                  xform_pose(outpose0, xalign)
                  outasym = rosetta.protocols.grafting.return_region(outpose0, 1,
nresasym)
                  ci = rosetta.core.io.CrystInfo()
                  ci.A(cell_spacing)  # cell dimensions
                  ci.B(cell_spacing)
                  ci.C(cell_spacing)
                  ci.alpha(90)  # cell angles n
                  ci.beta(90)
                  ci.gamma(90)
                  ci.spacegroup(xspec.spacegroup)  # sace group
                  pi = rosetta.core.pose.PDBInfo(outasym)
                  pi.set_crystinfo(ci)
                  outasym.pdb_info(pi)

                  sympose = outasym.clone()
                  rosetta.protocols.cryst.MakeLatticeMover().apply(sympose)
                  conf = sympose.conformation().clone()
                  assert rosetta.core.conformation.symmetry.is_symmetric(conf)
                  syminfo = rosetta.core.pose.symmetry.symmetry_info(sympose)

                  nasym = outasym.size()
                  conf.declare_chemical_bond(nasym, 'C', 1, 'N')
                  sympose.set_new_conformation(conf)
                  sympose.set_new_energies_object(
                     rosetta.core.scoring.symmetry.SymmetricEnergies())

                  sc = sfxn.score(sympose)
                  minscore = min(minscore, sc)
                  if sc > 7000:
                     continue

                  syminfo = rosetta.core.pose.symmetry.symmetry_info(sympose)
                  surfvol = rosetta.core.scoring.packing.get_surf_vol(sympose, 1.4)
                  peptvol = 0.0
                  for ir in range(1, syminfo.get_nres_subunit() + 1):
                     res = sympose.residue(ir)
                     ir = ir + 1  # rosetta numbering
                     for ia in range(1, res.natoms() + 1):
                        v = surfvol.vol[AtomID(ia, ir)]
                        if not np.isnan(v): peptvol += v
                  peptvol *= xspec.nsubs
                  print(f'peptvol {peptvol} {peptvol / cell_spacing**3}')
                  solv_frac = max(0.0, 1.0 - peptvol / cell_spacing**3)

                  tag = ''.join([
                     f'_cell{int(cell_spacing):03}_',
                     f'_sc{int(sc):05}_',
                     f'_solv{int(solv_frac*100):02}_',
                     f'{os.path.basename(pdbpath)}',
                     f'_nres{nresasym}',
```

```
                     f'{aa}_',
                     f'{len(results):06}',
                  ])
                  fn = kw.output_prefix + tag + '.pdb'

                  print('HIT %7.3f' % sc, outasym)
                  results.append([sc, cell_spacing, sympose])
                  if True:
                     outasym.dump_pdb(fn)

      print('minscore', minscore)

   results.sort

   if not results:
      print(f'{"":!^100}')
      print('NO RESULTS!!!')
      print(f'{"":!^100}')
      print('DONE')

   return results

validated_c3 = [
'<path_to_validated_pdbs/peptide.pdb>',
]

def get_test_kw(kw):
   if not kw.inputs:
      kw.inputs = ['mof/data/peptides/c.2.6_0001.pdb']
      print(f'{"":!^80}')
      print(f'{"no pdb list input, using test only_one":!^80}')
      print(f'{str(kw.inputs):!^80}')
      print(f'{"":!^80}')

   kw.spacegroups = ['p213']
   kw.aa_labels = ['BPY']
   kw.output_prefix = '_mof_test_c3c3' + '_'.join(kw.spacegroups) + '/'
   kw.angle_err_tolerance = 3
   kw.scale_number_of_rotamers = 0.25
   kw.max_bb_redundancy = 2.0
   kw.max_dun_score = 4.0
   kw.clash_dis = 3.3
   kw.contact_dis = 7.0
   kw.min_contacts = 0
   kw.max_score_minimized = 40.0
   kw.min_cell_size = 0
   kw.max_cell_size = 50
   kw.max_solv_frac = 0.80
   kw.debug = True
   # kw.continue_from_checkpoints = False
   return kw
```

Listing S5: We dock C3 peptides and C2 metal sites into three space groups ($I2_13$, $P4_132$ and $P4_332$).

```
import mof, rpxdock as rp, numpy as np
from rpxdock import homog as hm

from mof.pyrosetta_init import get_sfxn
```

```
from pyrosetta import rosetta as rosetta
from pyrosetta.rosetta.core.pose import Pose
from pyrosetta.rosetta.core.id import AtomID
from pyrosetta.rosetta.numeric import xyzVector_double_t as rVec
from pyrosetta import rosetta as rt, init as pyrosetta_init

class XtalSearchSpec(object):
   """stuff needed for pepdide xtal search"""
   def __init__(
         self,
         spacegroup,
         pept_axis,
         pept_orig,
         ligands,
         sym_of_ligand,
         max_dun_score,
         **kw,
   ):
      super(XtalSearchSpec, self).__init__()
      kw = rp.Bunch(kw)
      self.spacegroup = spacegroup
      self.pept_axis = pept_axis
      self.pept_orig = pept_orig
      self.ligands = ligands
      self.sym_of_ligand = sym_of_ligand
      self.max_dun_score = max_dun_score
      self.xtal_spec = mof.xtal_spec.get_xtal_spec(self.spacegroup)
      self.chm = rt.core.chemical.ChemicalManager.get_instance()
      self.rts = self.chm.residue_type_set('fa_standard')

      self.sfxn_rotamer = get_sfxn('rotamer')

      self.sfxn_sterics = get_sfxn('sterics')

      self.sfxn_minimize = get_sfxn('minimize')


def xtal_search_two_residues(
      search_spec,
      pose,
      rotcloud1base,
      rotcloud2base,
      err_tolerance,
      dist_err_tolerance,
      angle_err_tolerance,
      min_dist_to_z_axis,
      sym_axes_angle_tolerance,
      angle_to_cart_err_ratio,
      debug=False,
      **kw,
):
   kw = rp.Bunch(kw)
   if not kw.timer: kw.timer = rp.Timer().start()
   kw.timer.checkpoint()

   spec = search_spec
   xspec = spec.xtal_spec
   aa1 = rotcloud1base.amino_acid
   aa2 = rotcloud2base.amino_acid

   results = list()
```

```
   dont_replace_these_aas = [spec.rts.name_map(aa) for aa in
kw.dont_replace_these_aas]

   farep_orig = search_spec.sfxn_sterics(pose)

   p_n = pose.pdb_info().name().split('/')[-1]
   # gets rid of the ".pdb" at the end of the pdb name
   pdb_name = p_n[:-4]

   print(f'  {pdb_name} searching', aa1, aa2)

   # check the symmetry type of the pdb
   last_res = rt.core.pose.chain_end_res(pose).pop()
   total_res = int(last_res)

   sym_num = 3
   sym = 3
   if sym_num < 2:
      print('bad pdb', p_n)
      return list()
   asym_nres = int(total_res / sym)
   peptide_sym = "C%i" % sym_num

   rpxbody = rp.Body(pose)

   for ires1 in range(1, asym_nres + 1):
      if pose.residue_type(ires1) in dont_replace_these_aas: continue
      stub1 = rpxbody.stub[ires1 - 1]

      kw.timer.checkpoint('xtal_search')
      rots1ok = min_dist_to_z_axis < np.linalg.norm(
         (stub1 @ rotcloud1base.rotframes)[:, :2, 3], axis=1)
      if 0 == np.sum(rots1ok): continue
      rotcloud1 = rotcloud1base.subset(rots1ok)
      rotframes1 = stub1 @ rotcloud1.rotframes

      kw.timer.checkpoint('position rotcloud')

      range2 = range(1, int(total_res) + 1)
      if rotcloud1base is rotcloud2base: range2 = range(ires1 + 1, int(total_res) +
1)
      for ires2 in range2:
         if ires1 == ((ires2 - 1) % asym_nres + 1): continue
         if pose.residue_type(ires2) in dont_replace_these_aas:
            continue
         stub2 = rpxbody.stub[ires2 - 1]

         kw.timer.checkpoint('xtal_search')
         rots2ok = min_dist_to_z_axis < np.linalg.norm(
            (stub2 @ rotcloud2base.rotframes)[:, :2, 3], axis=1)
         if 0 == np.sum(rots2ok): continue
         rotcloud2 = rotcloud2base.subset(rots2ok)
         rotframes2 = stub2 @ rotcloud2.rotframes

         kw.timer.checkpoint('rotcloud positioning')

         dist = rotframes1[:, :, 3].reshape(-1, 1, 4) - rotframes2[:, :,
3].reshape(1, -1, 4)
         dist = np.linalg.norm(dist, axis=2)

         kw.timer.checkpoint('rotcloud dist')
```

```
        dot = np.sum(
           rotframes1[:, :, 0].reshape(-1, 1, 4) * rotframes2[:, :, 0].reshape(1,
-1, 4), axis=2)
        ang = np.degrees(np.arccos(np.clip(dot, -1, 1)))
        ang_delta = np.abs(ang - 109.4712206)

        kw.timer.checkpoint('rotcloud ang')

        err = np.sqrt((ang_delta / angle_to_cart_err_ratio)**2 + dist**2)
        rot1err2 = np.min(err, axis=1)
        bestrot2 = np.argmin(err, axis=1)
        disterr = dist[np.arange(len(bestrot2)), bestrot2]
        angerr = ang_delta[np.arange(len(bestrot2)), bestrot2]
        ok = (rot1err2 < err_tolerance)
        ok *= (angerr < angle_err_tolerance)
        ok *= (disterr < dist_err_tolerance)

        hits1 = np.argwhere(ok).reshape(-1)

        kw.timer.checkpoint('rotcloud match check')

        if len(hits1):
           hits2 = bestrot2[hits1]
           hits = np.stack([hits1, hits2], axis=1)
           for ihit, hit in enumerate(hits):
              frame1 = rotframes1[hit[0]]
              frame2 = rotframes2[hit[1]]

              kw.timer.checkpoint('xtal_search')

              parl = (frame1[:, 0] + frame2[:, 0]) / 2.0
              perp = rp.homog.hcross(frame1[:, 0], frame2[:, 0])
              metalaxis1 = rp.homog.hrot(parl, +45) @ perp
              metalaxis2 = rp.homog.hrot(parl, -45) @ perp
              symang1 = rp.homog.line_angle(metalaxis1, spec.pept_axis)
              symang2 = rp.homog.line_angle(metalaxis2, spec.pept_axis)
              match1 = np.abs(np.degrees(symang1) - xspec.dihedral) <
sym_axes_angle_tolerance
              match2 = np.abs(np.degrees(symang2) - xspec.dihedral) <
sym_axes_angle_tolerance
              if not (match1 or match2): continue
              matchsymang = symang1 if match1 else symang2
              metal_axis = metalaxis1 if match1 else metalaxis2
              if rp.homog.angle(metal_axis, spec.pept_axis) > np.pi / 2:
                 metal_axis[:3] = -metal_axis[:3]
              metal_pos = (rotframes1[hit[0], :, 3] + rotframes2[hit[1], :, 3]) /
2.0

              correction_axis = rp.homog.hcross(metal_axis, spec.pept_axis)
              correction_angle = np.abs(matchsymang - np.radians(xspec.dihedral))


              for why_do_i_need_this in (-correction_angle, correction_angle):
                 metal_axis_try = rp.homog.hrot(correction_axis,
why_do_i_need_this) @ metal_axis
                 if np.allclose(rp.homog.angle_degrees(metal_axis_try,
spec.pept_axis),
                              xspec.dihedral, atol=0.001):
                    metal_axis = metal_axis_try
                    break

              assert np.allclose(rp.homog.angle_degrees(metal_axis,
```

```
spec.pept_axis),
                                  xspec.dihedral, atol=0.001)

               kw.timer.checkpoint('axes geom checks')


               pose2mut = mof.util.mutate_two_res(pose, ires1, aa1,
rotcloud1.rotchi[hit[0]],
                                                  ires2, aa2,
rotcloud2.rotchi[hit[1]], sym_num)

               search_spec.sfxn_sterics(pose2mut)
               sc_2res = (pose2mut.energies().residue_total_energy(ires1) +
                          pose2mut.energies().residue_total_energy(ires2))
               sc_2res_orig = (pose.energies().residue_total_energy(ires1) +
                               pose.energies().residue_total_energy(ires2))

               kw.timer.checkpoint('mut_two_res')

               if sc_2res - sc_2res_orig > kw.max_2res_score: continue

               tag = ('hit_%s_%s_%i_%i_%i' % (aa1, aa2, ires1, ires2, ihit))

               kw.timer.checkpoint('xtal_search')

               xtal_poses = mof.xtal_build.xtal_build(
                  pdb_name,
                  xspec,
                  aa1,
                  aa2,
                  pose2mut,
                  peptide_sym,
                  spec.pept_orig,
                  spec.pept_axis,
                  'C2',
                  metal_pos,
                  metal_axis,
                  rpxbody,
                  tag,
                  **kw,
               )
               if not xtal_poses: continue


               kw.timer.checkpoint('xtal_search')

               for ixtal, (xalign, xtal_pose, body_pdb, ncontact, enonbonded,
                           solv_frac) in enumerate(xtal_poses):

                  xtal_pose_min, mininfo = mof.minimize.minimize_mof_xtal(
                     spec.sfxn_minimize,
                     xspec,
                     xtal_pose,
                     **kw,
                  )
                  if not xtal_pose_min:
                     continue
                  if kw.max_score_minimized < mininfo.score:
                     continue
                     print('     ', xspec.spacegroup, pdb_name, aa1, aa2, 'a on
minimzied score',
                           mininfo.score)
```

```
                  continue
               celldim = xtal_pose.pdb_info().crystinfo().A()
               label = f"{pdb_name}_{xspec.spacegroup.replace('
','_')}_{tag}_cell{int(celldim):03}_ncontact{ncontact:02}_score{int(enonbonded):03}"

               info = mininfo.sub(  # adding to mininfo
                  label=label,
                  xalign=xalign,
                  ncontact=ncontact,
                  enonbonded=enonbonded,
                  sequence=','.join(r.name() for r in xtal_pose.residues),
                  solv_frac=solv_frac,
                  celldim=celldim,
                  spacegroup=xspec.spacegroup,
                  nsubunits=xspec.nsubs,
                  nres=xtal_pose_min.size() - 1,
               )
               bbcoords = np.array(
                  [(v[0], v[1], v[2]) for v in [[r.xyz(n)
                                                 for n in ('N', 'CA', 'C')]
                                                for r in
xtal_pose_min.residues[:-1]]]])
               bbpad = np.zeros(shape=(kw.max_pept_size - xtal_pose_min.size() +
1, 3, 3))
               info['bbcoords'] = np.concatenate([bbcoords, bbpad])

               results.append(
                  rp.Bunch(
                     xspec=xspec,
                     asym_pose_min=xtal_pose_min,
                     info=info,
                  ))
               ### debug crap
               if results:
                  print('  * HIT %10s %7s %7s %3i %3i %9s %-7.3f %5.3f %s' % (
                     xspec.spacegroup.replace(' ', '_'),
                     aa1,
                     aa2,
                     ires1,
                     ires2,
                     xtal_pose_min.sequence(),
                     info.score,
                     info.solv_frac,
                     pdb_name,
                  ))
               ### end debug crap

            kw.timer.checkpoint('build_result')

   kw.timer.checkpoint('xtal_search')

   return results

def xtal_search_single_residue(search_spec, pose, **kw):
   raise NotImplemntedError('xtal_search_single_residue needs updating')
   kw = rp.Bunch(kw)

   spec = search_spec
   xspec = spec.xtal_spec

   results = list()
```

```
   p_n = pose.pdb_info().name().split('/')[-1]
   pdb_name = p_n[:-4]

   print(f'{pdb_name} searching')

   # check the symmetry type of the pdb
   last_res = rt.core.pose.chain_end_res(pose).pop()
   total_res = int(last_res)
   sym_num = pose.chain(last_res)
   if sym_num < 2:
      print('bad pdb', p_n)
      return list()
   sym = int(sym_num)
   peptide_sym = "C%i" % sym_num

   for ires in range(1, int(total_res / sym) + 1):
      if pose.residue_type(ires) not in (spec.rts.name_map('GLY'),
spec.rts.name_map('ALA'),
                                         spec.rts.name_map('DALA')):
         continue
      lig_poses = util.mut_to_ligand(pose, ires, spec.ligands, spec.sym_of_ligand)
      bad_rots = 0
      for ilig, lig_pose in enumerate(lig_poses):
         mut_res_name, lig_sym = lig_poses[lig_pose]

         rotamers = lig_pose.residue(ires).get_rotamers()
         rotamers = util.extra_rotamers(rotamers, lb=-20, ub=21, bs=20)

         pose_num = 1
         for irot, rotamer in enumerate(rotamers):
            for i in range(len(rotamer)):
               lig_pose.residue(ires).set_chi(i + 1, rotamer[i])
            rot_pose = rt.protocols.grafting.return_region(lig_pose, 1,
lig_pose.size())

            if kw.debug:
               rot_pose.set_xyz(AtomID(rot_pose.residue(ires).atom_index('1HB'),
ires),
                                rVec(0, 0, -2))
               rot_pose.set_xyz(AtomID(rot_pose.residue(ires).atom_index('CB'),
ires),
                                rVec(0, 0, +0.0))
               rot_pose.set_xyz(AtomID(rot_pose.residue(ires).atom_index('2HB'),
ires),
                                rVec(0, 0, +2))

            spec.sfxn_rotamer(rot_pose)
            dun_score = rot_pose.energies().residue_total_energy(ires)
            if dun_score >= spec.max_dun_score:
               bad_rots += 1
               continue
            rpxbody = rp.Body(rot_pose)


            metal_orig = hm.hpoint(util.coord_find(rot_pose, ires, 'VZN'))
            hz = hm.hpoint(util.coord_find(rot_pose, ires, 'HZ'))
            ne = hm.hpoint(util.coord_find(rot_pose, ires, 'VNE'))
            metal_his_bond = hm.hnormalized(metal_orig - ne)
            metal_sym_axis0 = hm.hnormalized(hz - metal_orig)
            dihedral = xspec.dihedral
```

```
            rots_around_nezn = hm.xform_around_dof_for_vector_target_angle(
               fix=spec.pept_axis, mov=metal_sym_axis0, dof=metal_his_bond,
               target_angle=np.radians(dihedral))

            for idof, rot_around_nezn in enumerate(rots_around_nezn):
               metal_sym_axis = rot_around_nezn @ metal_sym_axis0
               assert np.allclose(hm.line_angle(metal_sym_axis, spec.pept_axis),
                                  np.radians(dihedral))

               newhz = util.coord_find(rot_pose, ires, 'VZN') + 2 *
metal_sym_axis[:3]

               aid = rt.core.id.AtomID(rot_pose.residue(ires).atom_index('HZ'),
ires)
               xyz = rVec(newhz[0], newhz[1], newhz[2])
               rot_pose.set_xyz(aid, xyz)

               tag = f'{pdb_name}_{ires}_{lig_poses[lig_pose][0]}_{idof}_{pose_num}'
               xtal_poses = mof.xtal_build.xtal_build(
                  pdb_name,
                  xspec,
                  aa1,
                  aa2,
                  rot_pose,
                  peptide_sym,
                  spec.pept_orig,
                  spec.pept_axis,
                  lig_sym,
                  metal_orig,
                  metal_sym_axis,
                  rpxbody,
                  tag,
               )

               if False and xtal_poses:
                  print('hoaktolfhtoia')
                  print(rot_pose)
                  print(pdb_name)
                  print(xspec)
                  rot_pose.dump_pdb('test_xtal_build_p213.pdb')
                  print(ires)
                  print(peptide_sym)
                  print(spec.pept_orig)
                  print(spec.pept_axis)
                  print(lig_sym)
                  print(metal_orig)
                  print(metal_sym_axis)
                  print('rp.Body(pose)')

                  xalign, xpose, bodypdb = xtal_poses[0]
                  print(xalign)
                  xpose.dump_pdb('xtal_pose.pdb')

                  assert 0

               for ixtal, (xalign, xtal_pose, body_pdb) in enumerate(xtal_poses):
                  celldim = xtal_pose.pdb_info().crystinfo().A()
                  fname = f"{xspec.spacegroup.replace('
','_')}_cell{int(celldim):03}_{tag}"
                  results.append(
                     mof.result.Result(
                        xspec,
```

```
                        fname,
                        xalign,
                        rpxbody,
                        xtal_pose,
                        body_pdb,
                     ))

            pose_num += 1

   return results

def hokey_position_atoms(pose, ires1, ires2, metal_pos, metalaxispos):
   znres1, znres2 = None, None
   znres1 = ires1
   znres2 = ires2
   znatom1 = '1HB'
   znatom2 = '1HB'
   axisatom1 = '2HB'
   axisatom2 = '2HB'
   for znres, znatom, axisatom in [(znres1, znatom1, axisatom1), (znres2, znatom2, 
axisatom2)]:
      pose.set_xyz(AtomID(pose.residue(znres).atom_index(znatom), znres),
                   rVec(metal_pos[0], metal_pos[1], metal_pos[2]))
      pose.set_xyz(AtomID(pose.residue(znres).atom_index(axisatom), znres),
                   rVec(metalaxispos[0], metalaxispos[1], metalaxispos[2]))
```

Listing S6: Crystal parameters such as resolution of amino acid rotamer sampling and deviation from ideal metal coordination geometry are specified.

```
import sys, argparse, rpxdock as rp

def default_cli_parser(parent=None, **kw):
  parser = parent if parent else argparse.ArgumentParser(allow_abbrev=False)
  addarg = rp.app.options.add_argument_unless_exists(parser)


  addarg("inputs", nargs="*", type=str, default=[], help='input structures')

  addarg('--aa_labels', type=str, nargs='*',                  default='CYS DCYS ASP 
DASP GLU DGLU HIS DHIS HISD DHISD'.split(),
         help='choices: CYS DCYS ASP DASP GLU DGLU HIS DHIS HISD DHISD',)
  addarg('--aa_pair_labels', type=str, nargs='*',             default=['ALL'], 
help='give in pairs (--aa_pair_labels A B C D yields A-B and C-B pairs) choices: CYS 
DCYS ASP DASP GLU DGLU HIS DHIS HISD DHISD')
  addarg('--angle_err_tolerance', type=float,                 default=15, 
help='max allowed angular deviation from ideal metal binding. applied early, so ok 
to be generous')
  addarg('--angle_to_cart_err_ratio', type=float,             default=20.0, 
help='lever distance to equate angular and cartesian errors. probably no reason to 
change, unless you know why')
  addarg('--chiresl_asp1', type=float,                        default=8.0, 
help='resolution of scanning for asp chi1')
  addarg('--chiresl_asp2', type=float,                        default=5.0, 
help='resolution of scanning for asp chi2')
  addarg('--chiresl_cys1', type=float,                        default=6.0, 
help='resolution of scanning for cys chi1')
  addarg('--chiresl_cys2', type=float,                        default=8.0,
```

```
help='resolution of scanning for cys chi2')
  addarg('--chiresl_glu1', type=float,                         default=6.0,
help='resolution of scanning for glu chi1')
  addarg('--chiresl_glu2', type=float,                         default=12.0,
help='resolution of scanning for glu chi2')
  addarg('--chiresl_glu3', type=float,                         default=6.0,
help='resolution of scanning for glu chi3')
  addarg('--chiresl_his1', type=float,                         default=3.0,
help='resolution of scanning for his chi1')
  addarg('--chiresl_his2', type=float,                         default=8.0,
help='resolution of scanning for his chi2')
  addarg('--clash_dis', type=float,                            default=3.3,
help='distance below which atoms "clash"')
  addarg('--cluster', action="store_true",                   default=False,
help='')
  addarg('--contact_dis', type=float,                          default=7.0,
help='max CB-CB distance between residue "neighbors"')
  addarg('--cst_ang_metal', type=float,                        default=109.47,
help='desired angle between metal liganding atoms')
  addarg('--cst_dis_metal', type=float,                        default=2.2,
help='desired distance from metal to liganding atoms')

  addarg('--cst_sd_cut_ang', type=float,                       default=0.01,
help='std dev of angular cutpoint constraint (lower is stronger constraint)')
  addarg('--cst_sd_cut_dih', type=float,                       default=0.1,
help='std dev of dihedral cutpoint constraint (lower is stronger constraint)')
  addarg('--cst_sd_cut_dis', type=float,                       default=0.01,
help='std dev of distance cutpoint constraint (lower is stronger constraint)')

  addarg('--cst_sd_metal_coo', type=float,                     default=0.5,
help='std dev of metal-O-C-O dihedral constraint (lower is stronger constraint)')
  addarg('--cst_sd_metal_dir', type=float,                     default=0.4,
help='std dev of ligand "orbital points at metal" angle constraint (lower is
stronger constraint)')
  addarg('--cst_sd_metal_lig_ang', type=float,                 default=0.4,
help='std dev of lig-metal-lig angle constraint (lower is stronger constraint)')
  addarg('--cst_sd_metal_lig_dist', type=float,                default=0.2,
help='std dev of metal-lig distance constraint (lower is stronger constraint)')
  addarg('--cst_sd_metal_olap', type=float,                    default=0.03,
help='std dev of distance between symmetric copies of metal constraint (lower is
stronger constraint)')

  addarg('--test_run', action="store_true",                       default=False,
help='ignores most flags and inputs, set to test values')
  addarg('--debug', action="store_true",                       default=False,
help='extra output and maybe modified behavior')
  addarg('--dist_err_tolerance', type=float,                   default=1.0,
help='max allowed deviation of symmetric metal overlap from 0. applied early, so ok
to be generous')
  addarg("--dont_replace_these_aas", nargs="*", type=str,      default=['PRO'],
help='AAs which should not be changed')
  addarg('--err_tolerance', type=float,                        default=2.0,
help='max allowed combination of dist_err and angle_err. applied early, so ok to be
generous')
  addarg('--max_2res_score', type=float,                       default=10.0,
help='max score delta upon placing rotamers')
  addarg('--max_bb_redundancy', type=float,                    default=0.1,
help='max non-aligned rms distance between any outputs')
  addarg('--max_cell_size', type=float,                        default=50,
help='maximum cell size (will be correlated to peptide size, maybe use
--max_solv_frac if you have mixed size peptides')
  addarg('--max_dun_score', type=float,                        default=6.0,
```

```
help='overall maximum DUN score ever allowed for consideration')
  addarg('--max_pept_size', type=int,                          default=10,
help='reserve output space for this many residues (keep same to match run results
together)')
  addarg('--max_score_minimized', type=float,                  default=50.0,
help='maximum score after minimization (including cst and all)')
  addarg('--max_solv_frac', type=float,                        default=0.8,
help='maximum solvent fraction accepted. this is highly approximate... recommend
spot-checking and adjusting as needed')
  addarg('--max_sym_score', type=float,                        default=100.0,
help='max nonbonded energy across xtal contacts (induding clash)')
  addarg('--maxdun_asp', type=float,                           default=5.0,
help='max dunbrak score for asp')
  addarg('--maxdun_cys', type=float,                           default=4.0,
help='max dunbrak score for cys')
  addarg('--maxdun_glu', type=float,                           default=5.0,
help='max dunbrak score for glu')
  addarg('--maxdun_his', type=float,                           default=5.0,
help='max dunbrak score for his')
  addarg('--min_cell_size', type=float,                        default=0,
help='minimum cell size')
  addarg('--min_contacts', type=float,                         default=0,
help='minimun number of res-res contacts across symmetric units')
  addarg('--min_dist_to_z_axis', type=float,                   default=5.0,
help='maybe dont change this')
  addarg('--output_prefix', type=str,
default='results/mofdock_',  help='prefix to output filenames')
  addarg('--overwrite', action="store_true",                   default=False,
help='')
  addarg('--rotcloud_cache', type=str,
default='.rotcloud_cache',   help='cache file location')
  addarg('--scale_number_of_rotamers', type=float,             default=1.0,
help='modify resolution of rotamers')
  addarg('--sfxn_minimize_weights', type=str,
default='minimize.wts',      help='minimization score func wts file')
  addarg('--sfxn_rotamer_weights', type=str,
default='rotamer.wts',       help='rotamer scanning score func wts file')
  addarg('--sfxn_sterics_weights', type=str,
default='sterics.wts',       help='clash checking score func wts file')
  addarg('--spacegroups', nargs='*', type=str,                 default=[],
help='list of spacegroups')
  addarg('--sym_axes_angle_tolerance', type=float,             default=5.0,
help='max deviation from crystal "magic angle"')
  addarg('--bb_break_dist', type=float,             default=3.0)

  addarg('--postprocess', action="store_true",                 default=False,
help='')
  addarg('--strip_rosetta_content_from_results', action="store_true",
default=False,               help='')


  parser.has_mof_args = True
  return parser

def get_cli_args(argv=None, parent=None, **kw):
  parser = default_cli_parser(parent, **kw)
  argv = sys.argv[1:] if argv is None else argv
  argv = rp.app.options.make_argv_with_atfiles(argv, **kw)
  options = parser.parse_args(argv)
  return rp.Bunch(options)

def defaults():
```

```
  return get_cli_args([])
```

Listing S6: Rosetta minimizes the resulting modeled lattices.

```
import os, mof, numpy as np, rpxdock as rp
from mof.pyrosetta_init import (rosetta as r, rts, makelattice, addcst_dis,
addcst_ang,
                                addcst_dih, name2aid, printscores)
from pyrosetta import AtomID
from pyrosetta.rosetta.numeric import xyzVector_double_t as xyzVec

def print_nonzero_energies(sfxn, pose):
   for st in sfxn.get_nonzero_weighted_scoretypes():
      print(st)

def minimize_mof_xtal(sfxn, xspec, pose, debug=False, **kw):
   kw = rp.Bunch(kw)

   nresasym = pose.size()
   beg = 1
   end = nresasym - 1
   metalres = rts.name_map('ZN')
   metalname = 'ZN'
   metalresnos = [nresasym, 2 * nresasym]  # TODO.. make not stupid
   metalnbonds = 4

   metalaid = AtomID(1, metalresnos[0])

   pose = pose.clone()
   r.core.pose.remove_lower_terminus_type_from_pose_residue(pose, beg)
   r.core.pose.remove_upper_terminus_type_from_pose_residue(pose, end)
   for ir in range(1, pose.size() + 1):
      if 'HIS' in pose.residue(ir).name():
         newname = pose.residue(ir).name().replace('HIS', 'HIS_D')
         newname = newname.replace('_D_D', '')
         r.core.pose.replace_pose_residue_copying_existing_coordinates(
            pose, ir, rts.name_map(newname))

   if False:
      tmp = pose.clone()
      r.core.pose.replace_pose_residue_copying_existing_coordinates(tmp,
metalresnos[0], metalres)
      makelattice(tmp)
      tmp.dump_pdb('before.pdb')
   makelattice(pose)
   if debug: print(f'minimize.py score initial...................
{sfxn(pose):10.3f}')

   syminfo = r.core.pose.symmetry.symmetry_info(pose)
   symdofs = syminfo.get_dofs()
   allowed_jumps = list()

   nxyz = pose.residue(beg).xyz('N')
   cxyz = pose.residue(end).xyz('C')
   nac, cac = None, None  # (N/C)-(a)djacent (c)hain
   for isub in range(1, syminfo.subunits()):
```

```
      othern = pose.residue((isub + 0) * nresasym + 1).xyz('N')
      otherc = pose.residue((isub + 1) * nresasym - 1).xyz('C')
      if nxyz.distance(otherc) < 2.0: cac = (isub + 1) * nresasym - 1
      if cxyz.distance(othern) < 2.0: nac = (isub + 0) * nresasym + 1
   assert nac and cac, 'backbone is weird?'
   if debug: print('peptide connection 1:', cac, beg)
   if debug: print('peptide_connection 2:', end, nac)


   f_metal_lig_dist = r.core.scoring.func.HarmonicFunc(kw.cst_dis_metal,
kw.cst_sd_metal_lig_dist)
   f_metal_lig_ang = r.core.scoring.func.HarmonicFunc(np.radians(kw.cst_ang_metal),
                                                      kw.cst_sd_metal_lig_ang)
   f_metal_olap = r.core.scoring.func.HarmonicFunc(0.0, kw.cst_sd_metal_olap)
   f_point_at_metal = r.core.scoring.func.HarmonicFunc(0.0, kw.cst_sd_metal_dir)
   f_metal_coo = r.core.scoring.func.CircularHarmonicFunc(0.0, kw.cst_sd_metal_coo)
   f_cut_dis = r.core.scoring.func.HarmonicFunc(1.328685, kw.cst_sd_cut_dis)
   f_cut_ang_cacn = r.core.scoring.func.HarmonicFunc(2.028, kw.cst_sd_cut_ang)
   f_cut_ang_cnca = r.core.scoring.func.HarmonicFunc(2.124, kw.cst_sd_cut_ang)
   f_cut_dih = r.core.scoring.func.CircularHarmonicFunc(np.pi, kw.cst_sd_cut_dih)
   f_cut_dihO = r.core.scoring.func.CircularHarmonicFunc(0.00, kw.cst_sd_cut_dih)

   ################### check cutpoint ##################

   conf = pose.conformation().clone()
   assert r.core.conformation.symmetry.is_symmetric(conf)
   pi = pose.pdb_info()
   conf.declare_chemical_bond(cac, 'C', beg, 'N')
   pose.set_new_conformation(conf)
   pose.set_new_energies_object(r.core.scoring.symmetry.SymmetricEnergies())
   pose.pdb_info(pi)
   if debug: print(f'minimize.py: score after chem bonds..........
{sfxn(pose):10.3f}')

   #############################################

   cst_cut, cst_lig_dis, cst_lig_ang, cst_lig_ori = list(), list(), list(), list()

   ############### chainbreaks ################

   cst_cut.append(addcst_dis(pose, cac, 'C ', beg, 'N', f_cut_dis))
   cst_cut.append(addcst_dis(pose, end, 'C ', nac, 'N', f_cut_dis))
   if debug: print(f'minimize.py: score after chainbreak dis......
{sfxn(pose):10.3f}')
   cst_cut.append(addcst_ang(pose, cac, 'CA', cac, 'C', beg, 'N ', f_cut_ang_cacn))
   cst_cut.append(addcst_ang(pose, cac, 'C ', beg, 'N', beg, 'CA', f_cut_ang_cnca))
   cst_cut.append(addcst_ang(pose, end, 'CA', end, 'C', nac, 'N ', f_cut_ang_cacn))
   cst_cut.append(addcst_ang(pose, end, 'C ', nac, 'N', nac, 'CA', f_cut_ang_cnca))
   if debug: print(f'minimize.py: score after chainbreak ang......
{sfxn(pose):10.3f}')

   cst_cut.append(addcst_dih(pose, cac, 'CA', cac, 'C', beg, 'N ', beg, 'CA',
f_cut_dih))
   cst_cut.append(addcst_dih(pose, end, 'CA', end, 'C', nac, 'N ', nac, 'CA',
f_cut_dih))
   cst_cut.append(addcst_dih(pose, cac, 'O ', cac, 'C', beg, 'N ', beg, 'CA',
f_cut_dihO))
   cst_cut.append(addcst_dih(pose, end, 'O ', end, 'C', nac, 'N ', nac, 'CA',
f_cut_dihO))
   if debug: print(f'minimize.py: score after chainbreak dihedral.
{sfxn(pose):10.3f}')
```

```
   ############## metal constraints ################

   for i, j in [(i, j) for i in metalresnos for j in metalresnos if i < j]:
      addcst_dis(pose, i, metalname, j, metalname, f_metal_olap)
   if debug:
      print(f'minimize.py: score after metal olap ......... {sfxn(pose):10.3f}')

   allowed_elems = 'NOS'
   znpos = pose.residue(metalresnos[0]).xyz(1)
   znbonded = list()
   for ir in range(1, len(pose.residues) + 1):
      res = pose.residue(ir)
      if not res.is_protein(): continue
      for ia in range(5, res.nheavyatoms() + 1):
         aid = AtomID(ia, ir)
         elem = res.atom_name(ia).strip()[0]
         if elem in allowed_elems:
            xyz = pose.xyz(aid)
            dist = xyz.distance(znpos)
            if dist < 3.5:
               if res.atom_name(ia) in (' OD2', ' OE2'):  # other COO O sometimes
closeish
                  continue
               znbonded.append(aid)
   if len(znbonded) != metalnbonds:
      print('WRONG NO OF LIGANDING ATOMS', len(znbonded))
      if debug:
         for aid in znbonded:
            print(pose.residue(aid.rsd()).name(),
pose.residue(aid.rsd()).atom_name(aid.atomno()))
            pose.dump_pdb('WRONG_NO_OF_LIGANDING_ATOMS.pdb')
            return None, None

   for i, aid in enumerate(znbonded):
      cst = r.core.scoring.constraints.AtomPairConstraint(metalaid, aid,
f_metal_lig_dist)
      cst_lig_dis.append(cst)
      pose.add_constraint(cst)

   if debug: print(f'minimize.py: score after metal dist .........
{sfxn(pose):10.3f}')

   for i, aid in enumerate(znbonded):
      ir, res = aid.rsd(), pose.residue(aid.rsd())
      if all(_ not in res.name() for _ in 'ASP CYS HIS GLU'.split()):
         assert 0, f'unrecognized res {res.name()}'
      if any(_ in res.name() for _ in 'ASP GLU'.split()):
         # metal comes off of OD1/OE1
         ir, coo = aid.rsd(), ('OD1 CG OD2' if 'ASP' in res.name() else 'OE1 CD
OE2').split()
         cst_lig_ori.append(
            addcst_dih(pose, ir, coo[0], ir, coo[1], ir, coo[2], metalaid.rsd(),
metalname,
                       f_metal_coo))
      else:
         if 'HIS' in res.name(): aname = 'HD1' if res.has('HD1') else 'HE2'
         if 'CYS' in res.name(): aname = 'HG'
         cst_lig_ori.append(
            addcst_ang(pose, ir, res.atom_name(aid.atomno()), metalaid.rsd(),
metalname, ir,
                       aname, f_point_at_metal))
```

```
   if debug: print(f'minimize.py: score after metal dir..........
{sfxn(pose):10.3f}')

   for i, iaid in enumerate(znbonded):
      for j, jaid in enumerate(znbonded[:i]):
         cst = r.core.scoring.constraints.AngleConstraint(iaid, metalaid, jaid,
f_metal_lig_ang)
         cst_lig_ang.append(cst)
         pose.add_constraint(cst)

   if debug: print(f'minimize.py: score after lig angle added.....
{sfxn(pose):10.3f}')

   ################ minimization ########################

   movemap = r.core.kinematics.MoveMap()
   movemap.set_bb(True)
   movemap.set_chi(True)
   movemap.set_jump(False)
   for i in allowed_jumps:
      movemap.set_jump(True, i)
   minimizer = r.protocols.minimization_packing.symmetry.SymMinMover(
      movemap, sfxn, 'lbfgs_armijo_nonmonotone', 0.01, True)  # tol, nblist
   if sfxn.has_nonzero_weight(r.core.scoring.ScoreType.cart_bonded):
      minimizer.cartesian(True)
   minimizer.apply(pose)
   if debug: print(f'minimize.py: score after min no scale........
{sfxn(pose):10.3f}')


   kw.timer.checkpoint(f'min scale 1.0')

   asym = r.core.pose.Pose()
   r.core.pose.symmetry.extract_asymmetric_unit(pose, asym, False)
   r.core.pose.replace_pose_residue_copying_existing_coordinates(asym,
metalresnos[0], metalres)

   if debug: print(kw.timer)

   info = rp.Bunch()
   info.score = sfxn(pose)

   ############### score component stuff ################
   st = r.core.scoring.ScoreType
   etot = pose.energies().total_energies()
   info.score_fa_atr = (etot[st.fa_atr])
   info.score_fa_rep = (etot[st.fa_rep])
   info.score_fa_sol = (etot[st.fa_sol])
   info.score_lk_ball = (etot[st.lk_ball] + etot[st.lk_ball_iso] +
etot[st.lk_ball_bridge] +
                         etot[st.lk_ball_bridge_uncpl])
   info.score_fa_elec = (etot[st.fa_elec] + etot[st.fa_intra_elec])
   info.score_hbond_sr_bb = (etot[st.hbond_sr_bb] + etot[st.hbond_lr_bb] +
etot[st.hbond_bb_sc] +
                             etot[st.hbond_sc])
   info.score_dslf_fa13 = (etot[st.dslf_fa13])
   info.score_atom_pair_constraint = (etot[st.atom_pair_constraint])
   info.score_angle_constraint = (etot[st.angle_constraint])
   info.score_dihedral_constraint = (etot[st.dihedral_constraint])
   info.score_omega = (etot[st.omega])
   info.score_rotamer = (etot[st.fa_dun] + etot[st.fa_dun_dev] + etot[st.fa_dun_rot]
+
```

```
                         etot[st.fa_dun_semi] + etot[st.fa_intra_elec] +
etot[st.fa_intra_rep] +
                         etot[st.fa_intra_atr_xover4] + etot[st.fa_intra_rep_xover4]
+
                         etot[st.fa_intra_sol_xover4])
    info.score_ref = (etot[st.ref])
    info.score_rama_prepro = (etot[st.rama_prepro])
    info.score_cart_bonded = (etot[st.cart_bonded])
    info.score_gen_bonded = (etot[st.gen_bonded])

    pose.remove_constraints()
    info.score_wo_cst = sfxn(pose)

    [pose.add_constraint(cst) for cst in cst_cut]
    info.score_cst_cut = sfxn(pose) - info.score_wo_cst
    pose.remove_constraints()

    [pose.add_constraint(cst) for cst in cst_lig_dis]
    [pose.add_constraint(cst) for cst in cst_lig_ang]
    [pose.add_constraint(cst) for cst in cst_lig_ori]
    info.score_cst_lig_ori = sfxn(pose) - info.score_wo_cst
    pose.remove_constraints()

    [pose.add_constraint(cst) for cst in cst_lig_dis]
    info.score_cst_lig_dis = sfxn(pose) - info.score_wo_cst
    pose.remove_constraints()

    [pose.add_constraint(cst) for cst in cst_lig_ang]
    info.score_cst_lig_ang = sfxn(pose) - info.score_wo_cst
    pose.remove_constraints()

    [pose.add_constraint(cst) for cst in cst_lig_ori]
    info.score_cst_lig_ori = sfxn(pose) - info.score_wo_cst
    pose.remove_constraints()

    return asym, info
```

Designing non-coordinating residues: Once the crystals are modeled and filtered for clashing, amino acids not involved in the metal coordination are designed using Rosetta. This optimizes the sequence for the best packing in the context of the crystal lattice while constraining the metal coordinating residues.

Listing S7: The following xml protocol is used to design macrocycles in the context of the 3D lattices.

```
<ROSETTASCRIPTS>
    <SCOREFXNS>
        <ScoreFunction name="beta" weights="ref2015" symmetric="1" />

        <ScoreFunction name="beta_ncs" weights="ref2015" symmetric="1" >
            <Reweight scoretype="dihedral_constraint" weight="1.0" />
        </ScoreFunction>

        <ScoreFunction name="beta_soft" weights="ref2015" symmetric="1" >
            <Reweight scoretype="fa_rep" weight="0.05" />
        </ScoreFunction>
```

```
        <ScoreFunction name="beta_cst_highhbond" weights="ref2015_cst.wts"
symmetric="true" >
            <Reweight scoretype="chainbreak" weight="25.0" />
            <Reweight scoretype="hbond_sr_bb" weight="10.0" />
            <Reweight scoretype="hbond_lr_bb" weight="10.0" />
        </ScoreFunction>

        <ScoreFunction name="beta_cst_highhbond_comp" weights="ref2015_cst.wts"
symmetric="true" >
            <Reweight scoretype="chainbreak" weight="25.0" />
            <Reweight scoretype="hbond_sr_bb" weight="10.0" />
            <Reweight scoretype="hbond_lr_bb" weight="10.0" />
            <Reweight scoretype="aa_composition" weight="1.0" />
            <Reweight scoretype="aspartimide_penalty" weight="1.0" />
        </ScoreFunction>
    </SCOREFXNS>

    <RESIDUE_SELECTORS>
        <ResidueName name="metal_binding"
residue_name3="ASP,GLU,HIS,CYS,DAS,DGU,DHI,DCS" />
        <Not name="designable" selector="metal_binding" />
        <Phi name="pos_phi" select_positive_phi="true"
ignore_unconnected_upper="false"/>
        <Not name="neg_phi" selector="pos_phi" />
        <And name="designable_posphi" selectors="designable,pos_phi" />
        <And name="designable_negphi" selectors="designable,neg_phi" />
        <Index name="asym_unit" resnums="1-3" />
        <Index name="select_first_res" resnums="1" />
        <Index name="select_last_res" resnums="3" />
        <And name="designable_asym_unit" selectors="designable,asym_unit" />
    </RESIDUE_SELECTORS>

    <PACKER_PALETTES>
        <CustomBaseTypePackerPalette name="palette"
additional_residue_types="AIB,DALA,DPHE,DILE,DLYS,DLEU,DMET,DASN,DPRO,DGLN,DARG,DSER
,DTHR,DVAL,DTRP,DTYR"/>
    </PACKER_PALETTES>

    <TASKOPERATIONS>

        <ExtraRotamersGeneric name="extrarot" ex1="true" ex2="true" ex3="false"
ex4="false" extrachi_cutoff="3" />
        <ReadResfile name="laa" filename="inputs/laa.resfile"
selector="designable_negphi" />
        <ReadResfile name="daa" filename="inputs/daa.resfile"
selector="designable_posphi" />
        <OperateOnResidueSubset name="no_design_metal_binding_positions"
selector="metal_binding" >
            <PreventRepackingRLT/>
        </OperateOnResidueSubset>
    </TASKOPERATIONS>

    <FILTERS>
        <ShapeComplementarity name="sc_filt" jump="1" verbose="1" min_sc="0.2"
write_int_area="1" confidence="0"/>
        <ScoreType name="fa_rep" score_type="fa_rep" threshold="4000"
scorefxn="beta" confidence="1" />
        <ScoreType name="fa_rep_reporter" score_type="fa_rep" threshold="2000"
scorefxn="beta" confidence="0" />
        <ScoreType name="total_score_reporter" score_type="total_score"
threshold="2000" scorefxn="beta" confidence="0" />
```

```
        <OversaturatedHbondAcceptorFilter name="oversat"
max_allowed_oversaturated="0" hbond_energy_cutoff="-0.1"
consider_mainchain_only="true" scorefxn="beta" />
    </FILTERS>
    <MOVERS>
        <MakeLatticeMover name="make_lattice" contact_dist="20" />
        TaskAwareSymMinMover name="min" scorefxn="beta" bb="0" chi="1" rb="1"
symdofs="A,B" task_operations="design_task" />

        <ModifyVariantType name="upper_cutpoints" add_type="CUTPOINT_UPPER"
residue_selector="select_first_res" />
       <ModifyVariantType name="lower_cutpoints" add_type="CUTPOINT_LOWER"
residue_selector="select_last_res" />

        <DeclareBond name="bond1" res1="9" res2="1" atom1="C" atom2="N"
add_termini="false" />

       <Small name="small_perturbation" angle_max="5.0" scorefxn="beta" />
       <FastRelax name="frlx1" repeats="1" scorefxn="beta_cst_highhbond"
min_type="dfpmin">
           <MoveMap name="frlx_mm1" >
              <Span begin="1" end="999" bb="false" chi="false" />
           </MoveMap>
       </FastRelax>
       <FastRelax name="frlx2" repeats="1" scorefxn="beta_cst_highhbond"
min_type="dfpmin">
           <MoveMap name="frlx_mm2" >
              <Span begin="1" end="999" bb="true" chi="true" />
           </MoveMap>
       </FastRelax>
       <AddCompositionConstraintMover name="aacomp_all" filename="inputs/all.comp"
selector="designable_asym_unit" />

       <PackRotamersMover name="fdes1" scorefxn="beta_cst_highhbond_comp"
task_operations="laa,daa,no_design_metal_binding_positions" packer_palette="palette"
/>
        <FastRelax name="frlx3" repeats="1" scorefxn="beta" min_type="dfpmin"
task_operations="extrarot" >
           <MoveMap name="frlx_mm3" >
              <Span begin="1" end="999" bb="true" chi="true" />
           </MoveMap>
        </FastRelax>


    </MOVERS>
    <APPLY_TO_POSE>
    </APPLY_TO_POSE>
    <PROTOCOLS>
        <Add mover="make_lattice" />
        <Add filter="sc_filt" />
        <Add filter="fa_rep" />
        <Add filter="fa_rep_reporter" />
        <Add filter="total_score_reporter" />
        <Add mover="small_perturbation" />
       <Add mover="aacomp_all" />
       <Add mover="fdes1" />
        <Add filter="sc_filt" />
        <Add filter="fa_rep" />
    </PROTOCOLS>
</ROSETTASCRIPTS>
```

Listing S8: The following flags file specifies the options used while designing the lattices.

```
-l inputs/all.list
-nstruct 1
-in:file:extra_res_fa HZD.params
-parser:protocol inputs/working_peptide_xtal_design.xml
-crystal_refine
-ex1
-ex2
-out:file:scorefile score.sc
-matdes::num_subs_building_block 2
-no_his_his_pairE
-per_chain_renumbering
-out:path:all output/
-detect_disulf false
-symmetry_definition CRYST1
-overwrite
-out:path:pdb outputs
```

Filtering modeled lattices: Modeled crystal lattices were filtered based on density and energy calculations. The density of each crystal model was calculated using the following script. Here the peptides are split into asymmetric units (i.e. a C3 peptide has three repeating asymmetric units). The mass of the asymmetric unit is calculated and multiplied by the number of asymmetric units in a unit cell. Then, the volume of the unit cell is also calculated and density is determined by dividing the mass by the volume and saved in a list.

Listing S9: Density calculations for designed crystal lattices.

```
## This script takes a list of pdb names without a path or a '.pdb' at the end.
## The mass of an asymmetric unit (aka one chain) is calculated.
## The unit cell volume is calculated based on CRYST1 line


from pyrosetta import *
import re
import argparse
import glob
import sys
import math
from pathlib import Path
from astropy.table import Table
from astropy.io import ascii

#takes a list of pdb names
def parse_arguments(argv):
    parser = argparse.ArgumentParser(description="takes list of peptide names")
    parser.add_argument('PEP_LIST', nargs = 1, type = str, help = 'List of pdb 
names')
    args = parser.parse_args()
    return args


#calculates the cubic volume of the unit cell. Takes in the file path to a pdb
def unit_cell_volume (pdb_file_path):
```

```
    file_path = Path(pdb_file_path)
    with open (file_path) as pdb:
        #read Cryst1 line
        cryst1 = []
        for line in pdb:
            if line.startswith("CRYST1"):
                cryst1 = (line).split()
        print("CRYST! LINE IS: ", cryst1)
        #extract x,y,z, and angles from cryst1 based on digit patterns
        x_y_z = [float(i) for i in cryst1[1:7]]

        #determine crystal system
        if x_y_z[0] == x_y_z[1] == x_y_z[2] and x_y_z[3] == x_y_z[4] == x_y_z[5] ==
90.00:
            crystal_system = "cubic"
        elif x_y_z[0] == x_y_z[1] != x_y_z[2] and x_y_z[3] == x_y_z[4] == 90.00 and
x_y_z[5] == 120.00:
            crystal_system = "hexagonal"
        elif x_y_z[0] == x_y_z[1] != x_y_z[2] and x_y_z[3] == x_y_z[4] == x_y_z[5]
== 90.00:
            crystal_system = "tetragonal"
        elif x_y_z[0] == x_y_z[1] == x_y_z[2] and x_y_z[3] == x_y_z[4] == x_y_z[5]
!= 90.00:
            crystal_system = "rhombohedral"
        elif x_y_z[0] != x_y_z[1] != x_y_z[2] and x_y_z[3] == x_y_z[4] == x_y_z[5]
== 90.00:
            crystal_system = "orthorhombic"
        elif x_y_z[0] != x_y_z[2] and x_y_z[3] == x_y_z[4] == 90.00 and x_y_z[5] !=
90.00:
            crystal_system = "monoclinic"
        elif x_y_z[0] != x_y_z[1] != x_y_z[2] and x_y_z[3] != x_y_z[4] != x_y_z[5]:
            crystal_system = "triclinic"

        #determine lattice centering and number of asu in a unit cell
        if cryst1[7] == "P":
            lattice_centering = "primitive"
            N_asu = "1"
        elif cryst1[7] == "I":
            lattice_centering = "body centered"
            N_asu = "2"
        elif cryst1[7] == "F":
            lattice_centering = "face centered"
            N_asu = "4"
        elif cryst1[7] == "A" or cryst1[7] == "B" or cryst1[7]== "C":
            lattice_centering = "base centered"
            N_asu = "2"
        elif cryst1[7] == "R":
            lattice_centering = "D centered"
            N_asu = "1"

        #calculate cubic volume of unit cell
        if crystal_system == "cubic":
            volume = x_y_z[0] * x_y_z[1] * x_y_z[2]
            return volume
        elif crystal_system == "hexagonal":
            volume = 0.866 * x_y_z[0] * x_y_z[0] * x_y_z[2]
            return volume
        elif crystal_system == "tetragonal":
            volume = x_y_z[0] * x_y_z[0] * x_y_z[2]
            return volume
        elif crystal_system == "rhombohedral":
            volume = [sqrt(1- 3 * (math.cos(x_y_z[3]))**2 + 2 *
```

```
(math.cos(x_y_z[3]))**3)] * (x_y_z[0] ** 3)
            return volume
        elif crystal_system == "orthorhombic":
            volume = x_y_z[0] * x_y_z[1] * x_y_z[2]
            return volume
        elif crystal_system == "monoclinic":
            volume = x_y_z[0] * x_y_z[1] * x_y_z[2] * math.sin(x_y_z[4])
            return volume
        elif crystal_system == "triclinic":
            volume = x_y_z[0] * x_y_z[1] * x_y_z[2]


        return volume

#calculates the mass of one chain. Takes in the file path to a pdb and the length of
the asymmetric unit.
def get_mass_asym_unit (pdb_file_path):
    file_path = Path(pdb_file_path)

    with open (file_path) as pdb:

        #read Cryst1 line
        cryst1=[]
        for line in pdb:
            if line.startswith("CRYST1"):
                cryst1 = (line).split()

        #extract x,y,z, and angles from cryst1 based on digit patterns
        x_y_z = cryst1[1]

    #use ref 2015 score function
    sfxn = get_score_function()

    #creates pose from one chain as the asymmetric unit
    pose = pose_from_pdb(pdb_file_path)


    #make a list of atom types in a pose and count the number of atoms in that list.
    Atoms = []
    for res in pose:
        for i in range(1, len(res.atoms())+1):
            Atoms.append(res.atom_name(i))


    #calculate mass of asym unit pose in grams/pose not moles
    mass = pyrosetta.rosetta.core.pose.mass(1,3,pose) / (6.022*10**23)

    #returns the mass and energy of the unit cell
    return [mass, sfxn(pose)]




if __name__ == '__main__':
    pyrosetta.init('-crystal_refine -in:file:extra_res_fa
/projects/peptides/mofs/params_files/HDZ.params' )

    args = parse_arguments(sys.argv)

    #takes in a list of pdb names. ex tmp.list
    input_file = args.PEP_LIST[0]
```

```
    data_rows = []

    with open (input_file) as f:
        for lines in f:

            designed_name = 'input/' + lines.rstrip()
            print('THIS IS THE PATH!!!!!' + designed_name)

            vol = (unit_cell_volume(designed_name))


 ##################REMEMBER TO CHANGE BASED ON SPACE GROUP######################
 ####density of P23 space group using multi = 12  ###############################
 ################################################################################
            density = round((get_mass_asym_unit(designed_name)[0]*12)/(vol*10**-24),
6)

            data_rows.append((lines.rstrip(), vol,  density) )

    #saves data in a table format
    data = Table(rows=data_rows, names= ('PDB', 'UNIT_CELL_VOLUME',
'Density_g/cm^3'))

    #export data as energy_data.list so it can be sorted and filtered later.
    ascii.write(data, 'energy_data_g_cm.list', overwrite=True, format='fixed_width'
)
```

Listing S10: The lattice energy landscape is estimated by draping the peptide sequence on all modeled lattices, then scoring the results using Rosetta's energy function.

```
import pyrosetta
from pyrosetta import rosetta

import numpy as np

pyrosetta.init('-extra_res_fa /home/csykang/scripts/Params/HZD.params
-in:file:fullatom -symmetry_definition CRYST1')
path_to_scaffolds =
str("/projects/peptides/mofs/mof_design/xtal_matches_WS/P4332_c3_9res_20200608/")

# This script will grab the sequences from the designed pdbs and determine the
extent to which a given sequence is favored by a given peptide xtal lattice
# This will be done by draping each sequence and circular permutations of the
sequence over a series of lattices, then scoring each draped sequence to see which
peptide + scaffold combinations work the best.

_DEBUG = False


#takes a list of pdb names
def read_in_pdbs():
    # Reads a pdbs.list file to get the paths to all of the pdbs I want to test,
    # then opens the pdbs with import_pose.pose_from_file
    # Returns a list, raw_pose_list, that contains all of the poses
    raw_pose_path_list = []
```

```
    raw_pose_list = []
    pdbslist = open("pdbs.list", "r")
    lines = pdbslist.read().splitlines()
    for line in lines:
        raw_pose_path_list.append(line)
    for path in raw_pose_path_list:
        raw_pose = rosetta.core.import_pose.pose_from_file(path)
        raw_pose_list.append(raw_pose)
    return raw_pose_list

def cyc_align(pose):
    # Takes in a pose, cyclizes it, then aligns it to the z-axis
    # Returns a pose
    pcm = rosetta.protocols.cyclic_peptide.PeptideCyclizeMover()
    ata=rosetta.protocols.cyclic_peptide.SymmetricCycpepAlign()
    pcm.apply(pose)
    ata.apply(pose)
    return pose

def mutate_residues(t,r,p):
    mut = rosetta.protocols.simple_moves.MutateResidue()
    mut.set_target(t)
    mut.set_res_name(r)
    mut.set_preserve_atom_coords(False)
    mut.apply(p)
    return p

def fix_HZD_termini(s):
    substring = ":"
    if s == "HZD":
        return "HIS"
    elif substring in s:
        return s.split(':')[0]
    else:
        return s



def output_data(seq_pdb, scaff_score_dictionary):
    seq_name = seq_pdb.split(".pdb")[0]

    file_name = 'drape_seq_summaries/sequence_{}_scores.txt'.format(seq_name)
    o = open(file_name, "w")
    o.write("DESIGNED SEQ PDB NAME: %s\n" % seq_pdb)

    for scaff in scaff_score_dictionary:
        o.write("SCAFFOLD: %s\t\t" % scaff)
        o.write("SEQ_PERMUTATION, SCORE: %s\n" % scaff_score_dictionary[scaff])

def minimize(p,sfxn):
    movemap = rosetta.core.kinematics.MoveMap()
    movemap.set_chi(True)
    movemap.set_bb(False)
    movemap.set_jump(False)
    minmover = pyrosetta.rosetta.protocols.minimization_packing.MinMover(movemap,
sfxn, 'linmin', 0.001, True)
    for i in range(5):
        minmover.apply(p)

def cyclic_perm(list_of_seq):
    n = len(list_of_seq)
    result = []
```

```
    for j in range(n):
        def f(l, k=j):
            return list(map(lambda i: l[i - k], range(n)))
        result.append(f)
    return result


def check_and_remove_variant(input_pose):
    ''' util function to remove the terminal variant
        in order to correctly calculate the distances between fragment pairs
        and find the correct atom (for example, "N", "C") to declare bond and
determine connecting direction
    '''
    for in_idx in range(1, input_pose.size()+1):
        upper =
input_pose.residue(in_idx).has_variant_type(pyrosetta.rosetta.core.chemical.VariantT
ype.UPPER_TERMINUS_VARIANT)
        if upper:
            pyrosetta.rosetta.core.pose.remove_variant_type_from_pose_residue(
input_pose, pyrosetta.rosetta.core.chemical.UPPER_TERMINUS_VARIANT, in_idx)

        lower =
input_pose.residue(in_idx).has_variant_type(pyrosetta.rosetta.core.chemical.VariantT
ype.LOWER_TERMINUS_VARIANT)
        if lower:
            pyrosetta.rosetta.core.pose.remove_variant_type_from_pose_residue(
input_pose, pyrosetta.rosetta.core.chemical.LOWER_TERMINUS_VARIANT, in_idx)

def create_xyz_array(input_pose):
    ''' go through all the heavyatoms of the pose
        extract the xyz coordinate, and put it into a hugh xyz numpy array
        and use a dictionary to keep track the residue and atom index for each numpy
array entry
    '''
    xyz_list = []
    idx_info_dict = {}
    count = 0
    for res_i in range(1, input_pose.size()+1):
        this_res = input_pose.residue(res_i)
        if this_res.is_virtual_residue(): continue
        this_aa3  = this_res.name3().strip()
        if this_aa3 == "ZN":continue
        for atom_i in range(1, 5):
            this_atom_name = this_res.atom_name(atom_i).strip()
            this_xyz = this_res.atom(this_atom_name).xyz()
            this_array = [this_xyz.x, this_xyz.y, this_xyz.z]
            xyz_list.append(this_array)
            idx_info_dict[count] = [res_i, atom_i, this_atom_name]
            count += 1
    return np.array(xyz_list), idx_info_dict

def connect_downstream_frag(input_lattice, ref_frag_key = 1, dist_cutoff = 2.0):
    from scipy.spatial.distance import cdist
    ''' This function will find the downstream fragment of a specified fragment in a
lattice
        And declare a bond between the fragment and the found downstream one.
        The purpose of the bond declaration is for correctly energy calculation
    '''
    ## ------- step1 extract all the fragments, and clean up by removing all the
terminal variants --------- ##
    fragments = input_lattice.split_by_chain()
    for frag_key, each_frag in enumerate(fragments, start=1):
```

```
        check_and_remove_variant(each_frag)

    ## go through fragments, create a dictionary storing the fragment information
    ## fragment key is actually also the chain index of the lattice, 1-based index
    frag_dict = {}
    for frag_key, each_frag in enumerate(fragments, start=1):
        xyz_array, idx_info_dict = create_xyz_array(each_frag)
        frag_dict[frag_key] = (each_frag, xyz_array, idx_info_dict)

    ## go through fragment,
    ## and find the fragment corresponding to the specified fragment (reference
fragment)
    ## and find its neighbor fragments
    ## it only need to connect the downstream one, due to the symmetry
    connections = {}
    for frag_key, frag_value in frag_dict.items():
        ## skip the specified fragment
        if frag_key == ref_frag_key: continue

        ## collect the query and ref information
        query_xyz       = frag_value[1]
        query_info_dict = frag_value[2]

        ref_xyz       = frag_dict[ref_frag_key][1]
        ref_info_dict = frag_dict[ref_frag_key][2]

        if query_xyz.size > 0:
            pair_dist = cdist(query_xyz, ref_xyz)
            query_indices, ref_indices = np.where(pair_dist < dist_cutoff)
            for query_idx, ref_idx in zip(query_indices, ref_indices):
                query_atom = query_info_dict[query_idx][2]
                ref_atom   =   ref_info_dict[  ref_idx][2]

                query_resnum = query_info_dict[query_idx][0]
                ref_resnum   =   ref_info_dict[  ref_idx][0]


                if f"{ref_atom}{query_atom}" == "CN":
                    if ref_frag_key == 1:
                        res1 = ref_resnum
                    else:
                        res1 = input_lattice.chain_end(ref_frag_key-1) + ref_resnum
                    atom1 = ref_atom
                    if frag_key == 1:
                        res2 = query_resnum
                    else:
                        res2 = input_lattice.chain_end(frag_key-1) + query_resnum
                    atom2 = query_atom
                    connections["down"] = ((ref_frag_key, frag_key), (res1, atom1,
res2, atom2))


    declare_bond = pyrosetta.rosetta.protocols.cyclic_peptide.DeclareBond()
    print(f'Declaring bond of the specified fragment {connections["down"][0][0]} to
the downstream fragment: {connections["down"][0][1]}')
    declare_bond.set(connections["down"][1][0], connections["down"][1][1],
connections["down"][1][2], connections["down"][1][3], add_termini = False)
    declare_bond.apply(input_lattice)
    return None

def connect_downstream_frag_simplified(input_lattice, ref_frag_key = 1, dist_cutoff
= 2.0):
```

```
    from scipy.spatial.distance import cdist
    ''' This function will find the downstream fragment of a specified fragment in a
lattice
        And declare a bond between this fragment and the found downstream one.
        The purpose of the bond declaration is for correctly energy calculation
    '''
    ## ------- step1 extract all the fragments --------- ##
    fragments = input_lattice.split_by_chain()

    ref_frag  = fragments[ref_frag_key]
    ref_indices = [x for x in range(1, ref_frag.size()+1) if
ref_frag.residue(x).is_protein()]
    if not ref_indices:
        print("please choose another ref fragment")
        return None
    ref_frag.sequence()
    ref_C_res = ref_frag.residue(ref_indices[-1])
    ref_C_xyz = ref_C_res.atom("C").xyz()
    ## go through fragments, create a list storing the connections between N and C
    connections = []
    for frag_key, each_frag in enumerate(fragments, start=1):
        if frag_key == ref_frag_key: continue
        frag_indices = [x for x in range(1, each_frag.size()+1) if
each_frag.residue(x).is_protein()]
        if not frag_indices: continue
        N_res = each_frag.residue(frag_indices[0])
        N_xyz = N_res.atom("N").xyz()
        CN_dist = np.linalg.norm(ref_C_xyz - N_xyz)
        if CN_dist < dist_cutoff:
            if ref_frag_key == 1:
                res1 = ref_indices[-1]
            else:
                res1 = input_lattice.chain_end(ref_frag_key-1) + ref_indices[-1]
            atom1 = "C"
            res2 = input_lattice.chain_end(frag_key-1) + frag_indices[0]
            atom2 = "N"
            connections.append( ((ref_frag_key, frag_key), (res1, atom1, res2,
atom2)) )

    declare_bond = pyrosetta.rosetta.protocols.cyclic_peptide.DeclareBond()
    for keys, connection in connections:
        print(f'Declaring bond of the specified fragment {keys[0]} to the downstream
fragment: {keys[1]}')
        declare_bond.set(connection[0], connection[1], connection[2], connection[3],
add_termini = False)
        declare_bond.apply(input_lattice)
    return None
##----------------------------------------------------------------------------------
---------##

# Read in designed pdbs
designed_pdbs = read_in_pdbs() # imports poses - requires a "pdbs.list" file
containing paths
sfxn = rosetta.core.scoring.ScoreFunctionFactory.create_score_function('ref2015')

# Make xml objs for specific Movers
xml = rosetta.protocols.rosetta_scripts.XmlObjects.create_from_string("""
<RESIDUE_SELECTORS>
    <Index name="peptide" resnums="1-9" />
</RESIDUE_SELECTORS>
<MOVERS>
    <MakeLatticeMover name="mlm" contact_dist="20" />
```

```
</MOVERS>
""")
mlm = xml.get_mover("mlm")

# Put designed sequences into a dictionary
dictionary_designed = {}
for pdb in designed_pdbs:
    pdb_name = pdb.pdb_info().name().split('/')[-1] # gets the pdb name for outputs
later
    res_index = list(range(1,rosetta.core.pose.chain_end_res(pdb,1)+1))

    seq_list = []
    for res in range(1, rosetta.core.pose.chain_end_res(pdb, 1)+1):
        seq_list.append(fix_HZD_termini(pdb.residue(res).name()))

    print("SEQ_LIST:", seq_list)

    seq_nozn_list = [x for x in seq_list if x != "ZN"]

    print("SEQ_LIST_NOZN:", seq_nozn_list)

    cp_seq_list = []
    for seq in cyclic_perm(seq_nozn_list): # cyclic permutations
        cp_seq_list.append(seq(seq_nozn_list))
        print(seq)

    dictionary_designed.update(dict({pdb_name : cp_seq_list}))

# depending on what you want, this could be either all of the scaffolds, or just the
scaffolds that are comparable (same metal binding ligands)
ext = str(".pdb")
dictionary_scaff = {}

for key in dictionary_designed.keys():
    list_of_tuples = []
    k = key.split('_0001.pdb')[0] # Needs to be checked to make sure the names still
work
    k_n = "".join((k,ext))
    s_l = "".join((path_to_scaffolds,k_n))
    scaff = rosetta.core.import_pose.pose_from_file(str(s_l))

    for resnum in range(1, int(rosetta.core.pose.chain_end_res(scaff,1))):
        print(resnum, scaff)
        mutate_residues(resnum,'GLY',scaff)

    mlm.apply(scaff) # apply MakeLatticeMover
    # This would be the best place to declare bonds on the poly gly lattices so that
they're fixed before being put into my dictionary of scaffolds, dictionary_scaff.

    connect_downstream_frag_simplified(scaff, ref_frag_key = 1, dist_cutoff = 2.0)

    polygly_score = round(sfxn(scaff), 2)

    list_of_tuples.append((scaff, polygly_score))
    dictionary_scaff.update(dict({k_n : list_of_tuples})) # key = name of the
scaffold, value = list of tuples containing (poly gly lattice pose (called "scaff")
and the score of "scaff")

    # Mutate each scaffold to the sequence saved in the dictionary
dictionary_scores = {}
for pep_seq in dictionary_designed:
    dictionary_seq_scaff = {}
```

```
    for scaff, info in dictionary_scaff.items():
        for scaff_pdb in info:
            permutation_counter = 1
            list_of_tuples = []
            for seq_permutation in dictionary_designed[pep_seq]:
                for resnum in range(1,
int(rosetta.core.pose.chain_end_res(scaff_pdb[0],1))):
                    mutated_pose =
mutate_residues(resnum,seq_permutation[resnum-1],scaff_pdb[0])

                #dbm.apply(mutated_pose)
                minimize(mutated_pose,sfxn) # to fix the wonky side chains

                mut_score = round(sfxn(mutated_pose), 2) # score the peptide, round
makes it output to 2 decimal places

                # Subtracting the poly_gly score from the "total_score" value so
that we're only looking at the effects of the designed residues.
                total_score = mut_score - scaff_pdb[1]

                if _DEBUG:
                    total_energies = mutated_pose.energies().total_energies()
                    fa_rep =
mutated_pose.energies().total_energies()[pyrosetta.rosetta.core.scoring.ScoreType.fa
_rep]
                    print("POLY_GLY_only_score: ", scaff_pdb[1])
                    print("TOTAL:", total_score)

mutated_pose.dump_pdb('sequence_{}_permutation_number_{}_in_scaffold_{}'.format(pep_
seq.split(".pdb")[0].split("mofdock_asym_")[1],permutation_counter, scaff))

                list_of_tuples.append((permutation_counter, total_score))
                dictionary_seq_scaff.update(dict({scaff : list_of_tuples}))

                permutation_counter +=1

    output_data(pep_seq, dictionary_seq_scaff) # output data to a .txt file.
```

Supplementary Data

Analytical UPLC and LCMS spectra for each peptide

Percent purity is calculated based on area integration of the analytical plot.

Peptide C2-1: (3-(4-Pyridyl)-alanine - β-Homoproline - α-Aminobutyric acid - 3-(4-Pyridyl)-alanine - β-Homoproline - α-Aminobutyric acid)

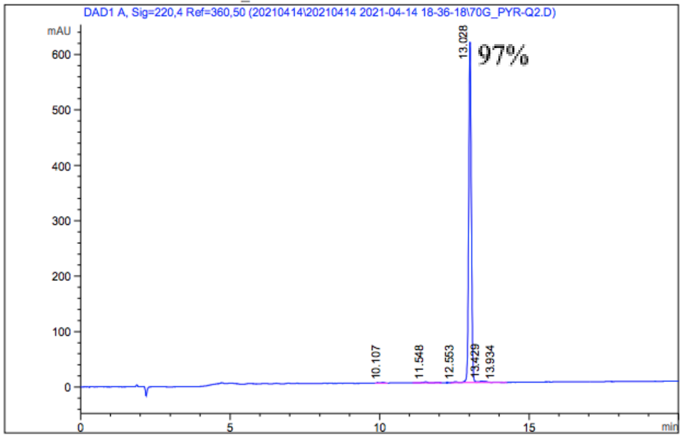

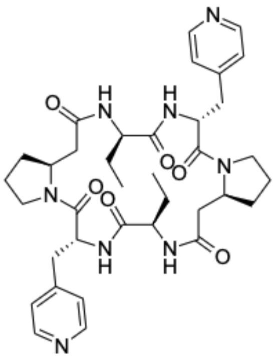

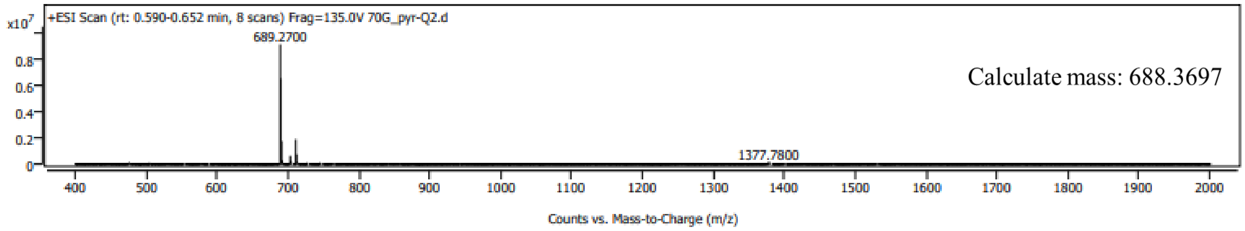

Peptide C2-2: (3-(4-Pyridyl)-alanine -1,2,3,4-tetrahydroisoquinoline-3-carboxylic acid - 3-Aminobutanoic acid -3-(4-Pyridyl)-alanine - 1,2,3,4-tetrahydroisoquinoline-3-carboxylic acid - 3-Aminobutanoic acid)

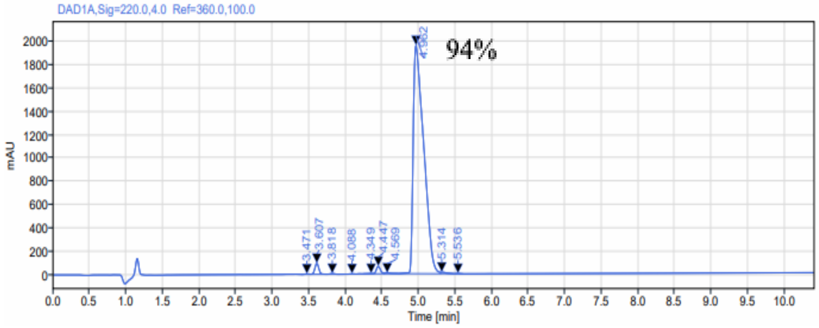

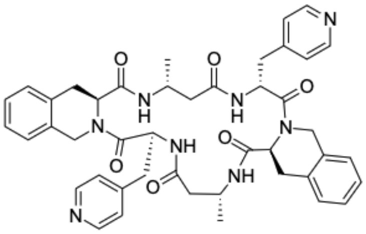

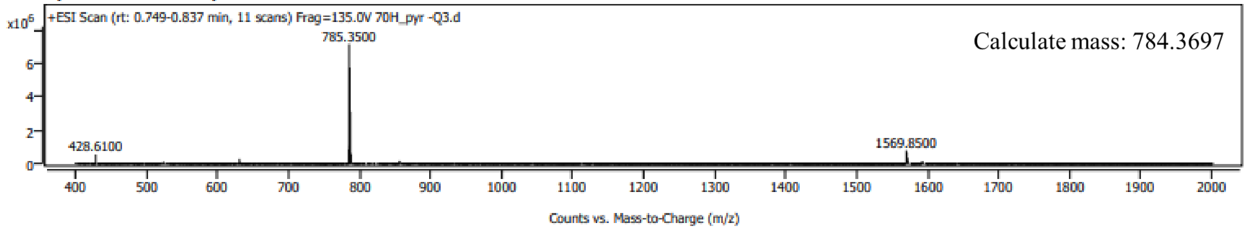

Peptide C3-1: (EhPEhPEhP)

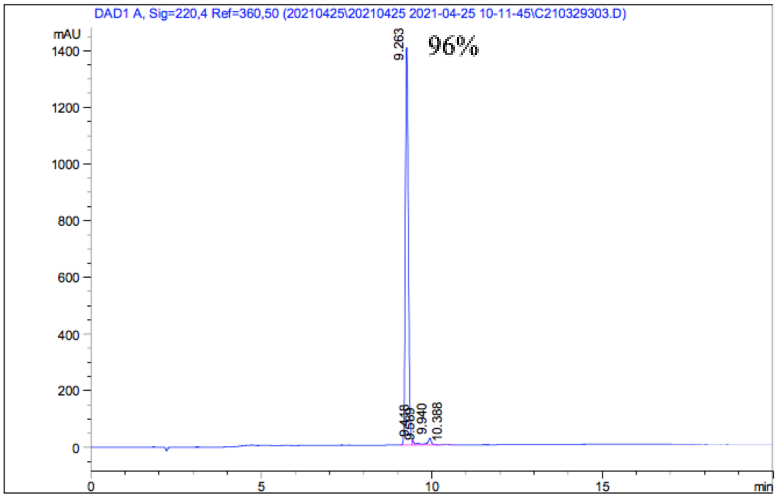

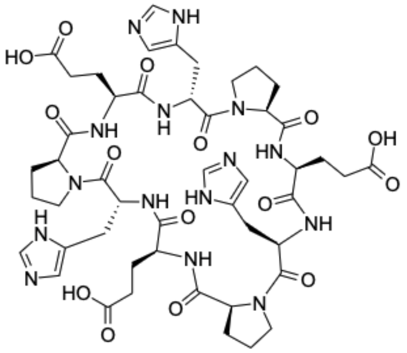

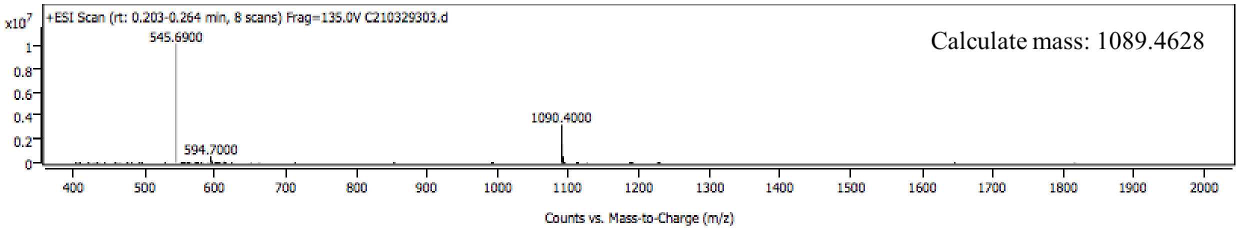

Peptide C3-2: (DhmDhmDhm)

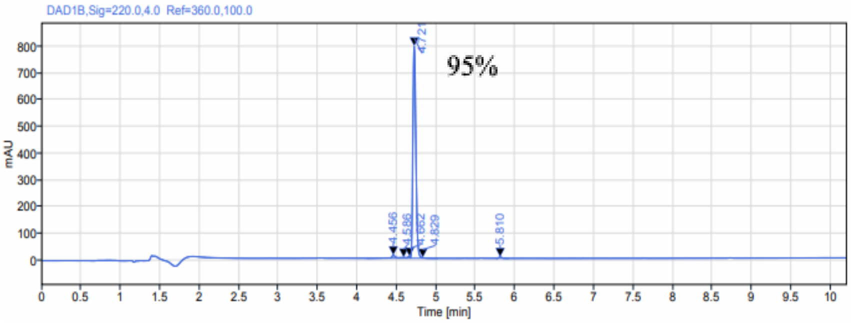

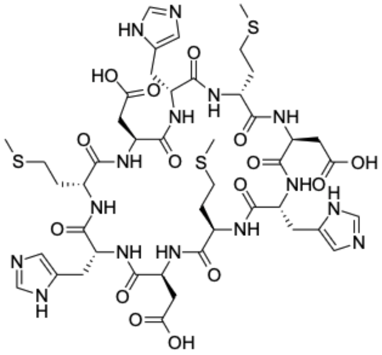

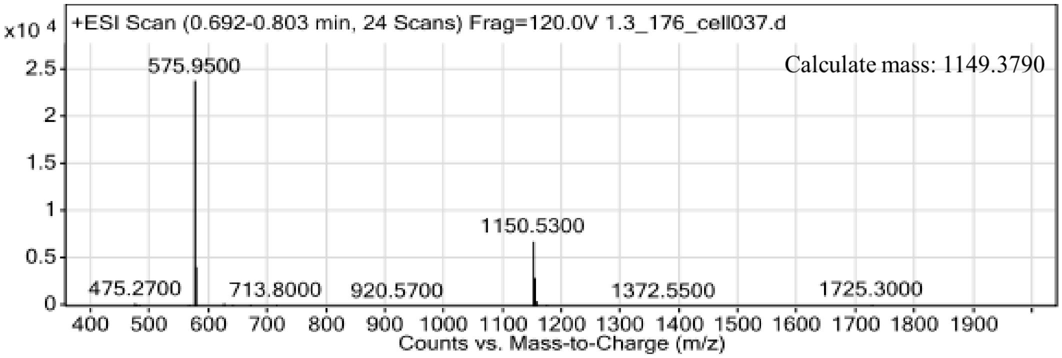

Peptide S2-1: (ppKvEPPkVe)

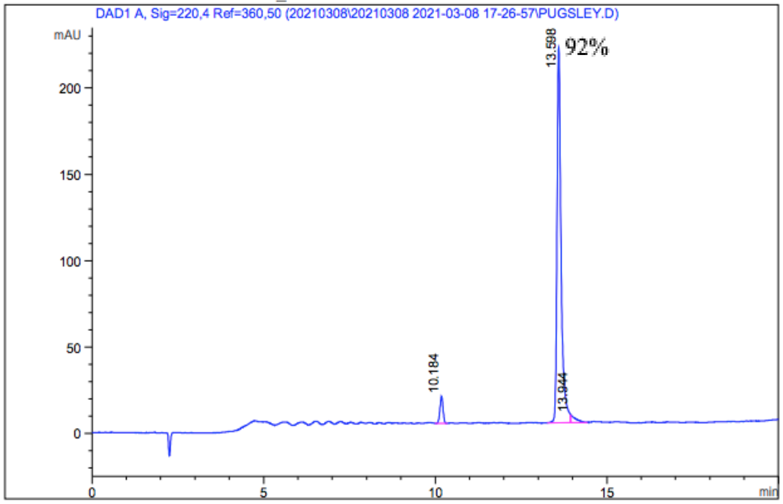

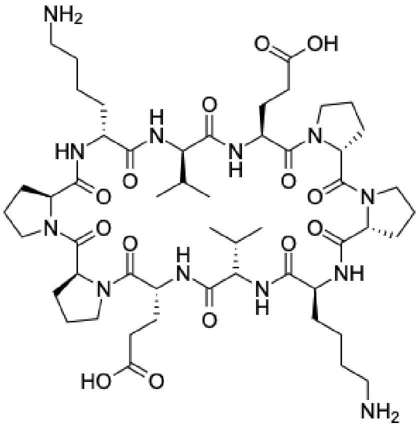

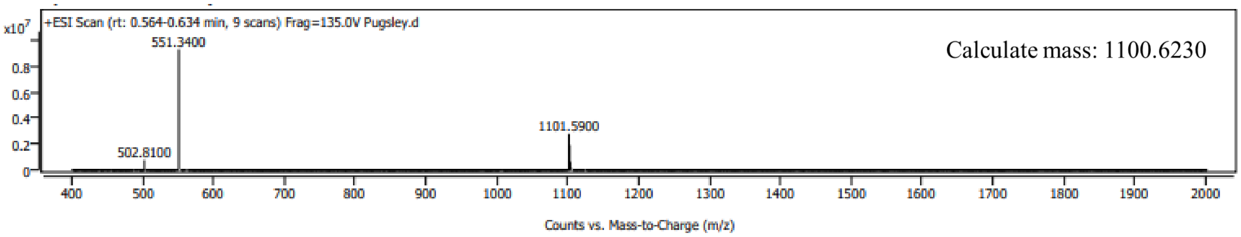

Peptide S2-2: (aNkhPeAnKHpE)

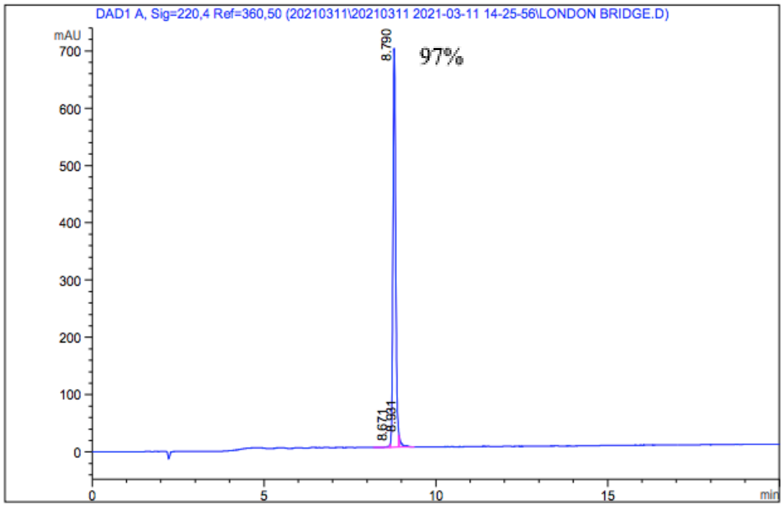

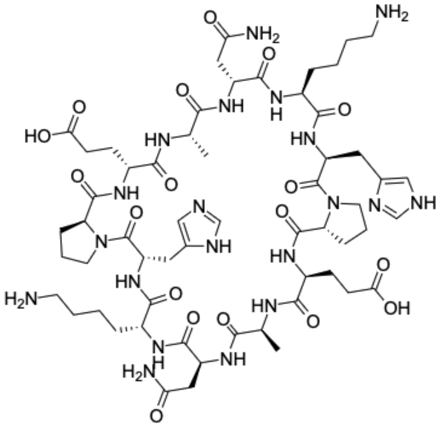

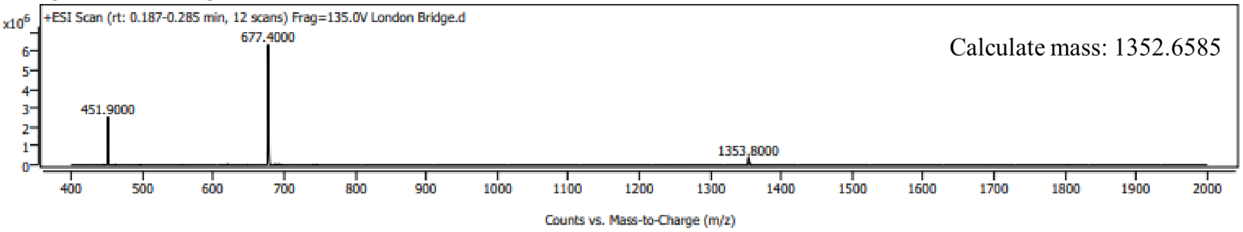

Supplement: Supplementary file 1 — cm2c02597_si_001.pdf [file cm2c02597_si_001.pdf]
